# The statistical building blocks of animal movement simulations

**DOI:** 10.1186/s40462-024-00507-4

**Published:** 2024-09-30

**Authors:** Wayne M. Getz, Richard Salter, Varun Sethi, Shlomo Cain, Orr Spiegel, Sivan Toledo

**Affiliations:** 1 Department Environmental Science, Policy and Management, University of California, Berkeley, CA 94720 USA; 2 School of Mathematics, Statistics & Computer Science, University of KwaZulu-Natal, Durban, South Africa; 3 Numerus Inc., 850 Iron Point Road, Folsom, CA 95630 USA; 4 Department of Computer Science, Oberlin College, Oberlin, OH 44074 USA; 5 School of Zoology, Faculty of Life Sciences, Tel Aviv University, 69978 Tel Aviv, Israel; 6 Blavatnik School of Computer Science, Tel Aviv University, 69978 Tel Aviv, Israel

**Keywords:** Multiscale movement analysis, Movement path segmentation, Individual-based models (IBM), Numerus ANIMOVER, Barn owl *Tyto alba*, Hierarchical cluster analysis

## Abstract

Animal movement plays a key role in many ecological processes and has a direct influence on an individual’s fitness at several scales of analysis (i.e., next-step, subdiel, day-by-day, seasonal). This highlights the need to dissect movement behavior at different spatio-temporal scales and develop hierarchical movement tools for generating realistic tracks to supplement existing single-temporal-scale simulators. In reality, animal movement paths are a concatenation of fundamental movement elements (FuMEs: e.g., a step or wing flap), but these are not generally extractable from a relocation time-series track (e.g., sequential GPS fixes) from which step-length (SL, aka velocity) and turning-angle (TA) time series can be extracted. For short, fixed-length segments of track, we generate their SL and TA statistics (e.g., means, standard deviations, correlations) to obtain segment-specific vectors that can be cluster into different types. We use the centroids of these clusters to obtain a set of statistical movement elements (StaMEs; e.g.,directed fast movement versus random slow movement elements) that we use as a basis for analyzing and simulating movement tracks. Our novel concept is that sequences of StaMEs provide a basis for constructing and fitting step-selection kernels at the scale of fixed-length canonical activity modes: short fixed-length sequences of interpretable activity such as dithering, ambling, directed walking, or running. Beyond this, variable length pure or characteristic mixtures of CAMs can be interpreted as behavioral activity modes (BAMs), such as gathering resources (a sequence of dithering and walking StaMEs) or beelining (a sequence of fast directed-walk StaMEs interspersed with vigilance and navigation stops). Here we formulate a multi-modal, step-selection kernel simulation framework, and construct a 2-mode movement simulator (Numerus ANIMOVER_1), using Numerus RAMP technology. These RAMPs run as stand alone applications: they require no coding but only the input of selected parameter values. They can also be used in R programming environments as virtual R packages. We illustrate our methods for extracting StaMEs from both ANIMOVER_1 simulated data and empirical data from two barn owls (*Tyto alba*) in the Harod Valley, Israel. Overall, our new bottom-up approach to path segmentation allows us to both dissect real movement tracks and generate realistic synthetic ones, thereby providing a general tool for testing hypothesis in movement ecology and simulating animal movement in diverse contexts such as evaluating an individual’s response to landscape changes, release of an individual into a novel environment, or identifying when individuals are sick or unusually stressed.

## Introduction

One of the major challenges common to several subfields of ecology (e.g., conservation biology, disease ecology, resource ecology) is predicting how the movement of animals changes in response to landscape factors and the state or health of the individual [1–3]. Examples of change include the spatio-temporal distribution of resources vital to the existence of individuals [3, 4], the movement of animals released into new surroundings for the purposes of conservation [2, 5], or the movement of individuals under stress or with infections [6, 7]. The dynamic resource example has led to the concept of resource tracking, which has become an active field of research in movement ecology [8–10]. Quantification of this process through models that link animal movement to resources will enable us to better predict the consequences of global change on animal populations [11]. The stressed individual example may be critical to the continued existence of endangered species [12]. The third example may help us identify and selectively remove individuals that are sick, and hence reduce the risk of pandemic outbreaks [13].

Movement, whether simulated or real, generates complex patterns that require various approaches to classify and comprehend. The primary approach to deconstructing this complexity, which has been ongoing for at least 30 years [14–17], has been to organize the movement track of animals into two or more different movement modes, and to parse the movement tracks of animals into consecutive segments each representing a different mode of movement from the previous segment. The primary quantitative methods that have been developed to carry out this type of path segmentation have been behavioral change point analysis (BCPA) [18–23] and hidden Markov methods (HMM) [24–28].

**Fig. 1:**
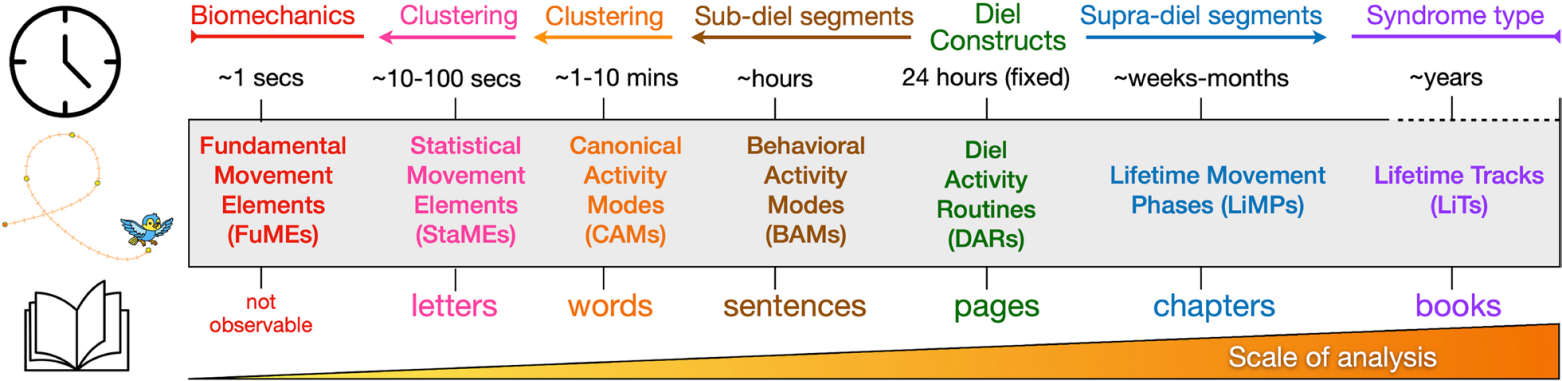
A facile comparison of a hierarchical movement track segmentation scheme (second ribbon from bottom) with hierarchical text elements (bottom ribbon) suggestive of the scheme’s utility to provide a movement track narrative of an animal’s life history. The listed time scale (second ribbon from the top) roughly applies to medium to large vertebrates. The top ribbon indicates that fixed-time DARs provide an anchor for the segmentation scheme, below and above which clustering and change-point methods of analysis can be used to respectively identify sub and supra diel segments (also see Appendix A.2, SOF). The StaME approach explored in this paper, provides a basic set of elements that can be used to hierarchically construct higher order elements, such as CAMs, BAMs, and DARs [34]

An alternative approach to path segmentation is to view movement tracks as a hierarchical organization of segments with levels that have relevance at different spatiotemporal scales of analysis [29–32] (Fig 1; also see Appendix A.1, SOF = supplementary online file). The value of an hierarchical approach is abundantly evident as an epistemological tool for deconstructing complexity (e.g., the construction of texts and genomes), but requires some building-block basis (e.g., letters for texts, codons for genetic coding of proteins), whether real or imagined, for the hierarchical construction [33].

In the context of movement tracks, the real building blocks are fundamental movement elements (FuMEs; [30]—e.g., for horses this may be walk, trot, cantor orand gallop elements that when strung in sequence constitute, walking trotting, cantering and galloping, etc.), but these cannot be extracted from relocation data, even when such data have a resolution on the order of seconds. Identification of individual FuMEs as a animal moves through space is likely to require either analyses of videos of the movements, inferences using accelerometers data collected from particular locations on the animals body [35], or other types of data collected from sufficiently fast sensors to identify the start and end of each type of movement element (e.g.,a wing beat of a bird [36]). In the absence of being able to identify actual FuMEs, we propose the identification of statistical movement elements (StaMEs) as the smallest achievable building block elements for the hierarchical construction of animal movement tracks (Fig 1).

The purpose of this paper is to meet the following three goals:

- Explore the potential of StaMEs as substitutes for FuMEs in providing a set of basic building block upon which next-level CAM segments of fixed size (number of steps), can be constructed and used to generate a further-level of variable-length behavioral activity modes (BAMs) (Fig 1)
- Formulate a multi-mode canonical activity movement (CAM) framework, based on the implementation of step-selection kernels, with switching among kernels influenced by landscape structure (cellular arrays of resources and topographic measures), environmental variables (e.g., temperature, precipitation), and internal variables (e.g., surrogates for hunger, thirst, or diel schedules)
- Provide a highly flexible, user friendly, freely available 2-movement-mode simulator in the form of a computer application package (Numerus Studio platform plus simulator application, both downloadable for free at links provided in Appendix C (SOF) and demonstrate its utility for movement ecologists to generate multi-modal movement tracks using step-selection methods and test hypothesis regarding mechanisms producing emergent patterns of movement.

This paper should be seen as part of a larger body of work that includes the formulation of a general framework for hierarchical track segmentation, as summarized in Fig 1 and discussed more fully in [30, 37]. In parallel, we are also generating measures that can be used to rigorously analyze bottom-up path segmentation methods using information theory measures of coding efficiency [34, 38].

Empirically, the movement track of an individual over a landscape is generally represented by a sequence of locations that is recorded using GPS technology [39], ATLAS reverse GPS technology [40], acoustic receivers or other technologies [41]. From such sets of relocation points, also referred to as a “walk,” step-length (SL; also velocities when the sampling frequency is fixed) and turning-angle (TA) time series can be extracted [30] (Appendix A.2, SOF). These time series can then be used to compute various derived quantities, such as radial and tangential velocities at each relocation point, and auto correlations of variables along segments of the movement track [18]. The statistics of such variables, computed for fixed short segments of track (e.g., 10–30 points), can then be used to categorize such segments into statistical movement elements (i.e., StaMEs previously called referred to as metaFuMEs in [30]).

These StaMEs can then be classified into a limited number of categories, as demonstrated in this paper (e.g.,short elements underpinning direct fast flight, brisk walking, meandering, and so on). A string of same category StaMEs then constitutes a track segment that can be classified as a homogeneous or canonical activity mode (CAM) of a type defined by the underlying category of StaME (e.g., brisk walking might translate into bee-lining and meandering into searching behavior). Characteristic mixtures of CAMs, in turn, can be strung together into identifiable behavioral activity modes (BAMs; e.g., resting, foraging, heading to a known location while being vigilant), with several BAMs coming together each day to form a diel activity routine (DAR) [42, 43] (Fig 1. The DAR itself is a hierarchical segment that can be understood in terms of an invariant 24-hour period for most animals, apart from some deep dwelling marine or cave-dwelling species, because for most species it is a fundamental biological rhythm honed by evolution [44]. The periods of various exogenous environmental cycles around or beyond the diel period (e.g., lunar, seasonal, or tidal), though, can be quite variable in their effects on species, depending on the latitude [45], elevation [46] and the trophic levels (e.g., herbivore, predator, scavenger) at which they function.

If the relocation sampling frequency is relatively high (i.e., approximately 5 or more relocation points per min), then the statistical properties of a segment of, say, 10–30 consecutive points (e.g., the means of the velocities and others) can serve to construct a set of StaMEs, which may then be classified into a relatively small set of StaME categories and associated canonical activity modes. From our presentation here, it will become clear that StaMEs are dependent, firstly, on the resolution (i.e., frequency) of the relocation data and, secondly, on the number of consecutive points used to derive the statistics of our StaMEs (i.e., its duration). Since some of the measures and features used to characterize movement track segments using relocation data are noticeably frequency dependent [15, 47], these will be influenced by the scale at which we define the underlying set of StaMEs used to reconstruct movement track segments of various lengths.

Movement, of course, does not occur in a vacuum and the statistics of the movement elements are going to be affected by landscape factors (e.g., slope and roughness of the terrain) and various environmental conditions (e.g., resources, temperature, wind, ocean currents, etc.) [48–50]. The effects of such factors will induce additional variation or noise in the statistics of StaMEs of different types. If all we have is a movement relocation time series without the benefit of covariate variables to provide context, then ignoring such covariates will add some noise to the process of identifying an underlying set of movement track StaMEs. If such data are available, then different sets of StaMEs can be identified for segments occurring for particular ranges of covariate values. Otherwise, modifications to the StaME statistics can be made under the assumptions that for each type of StaME identified for the track as a whole, modifications are needed when particular covariate conditions exist (e.g.,terrains that exceed a steepness threshold or when prevailing winds exceed a wind velocity threshold).

Elaborating on goal c.), our application is called ANIMOVER_1 (**ANI**mal **MOVE**ment **R**AMP; the “1” anticipates future elaborated versions) and the acronym RAMP refers to Numerus’ highly flexible **R**untime **A**lterable **M**odeling **P**latform technology [37]. We stress that this application platform is not a general programming environment and does not require any coding experience. However, it allows the user to set parameter values and, if desired, to overwrite critical lines of default code pertaining to landscape generating algorithms and agent movement rules to meet the user’s specific needs. As in integral part of this paper, we thus discuss why the construction of our ANIMOVER_1 RAMP facilitates analyses of movement pathways by ecologists compared with the effort needed to code up movement simulations from scratch using current programming platforms, such as Netlogo [51]. In addition, we focus on issues relating to parameter selection and running the model, as well as classifying StaMEs derived from both simulated and real data into a limited number of types (in our case 8 categories using hierarchical clustering methods). The real data relate to the movement data of barn owls (*Tyto alba*) in the Harod Valley, Israel, as more fully discussed in other contexts elsewhere [43, 52, 53].

## Model framework

### Arrays and movement kernels

**Fig. 2:**
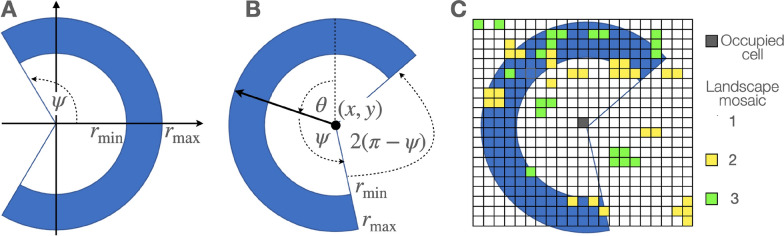
Kernels (**A** area in blue) are specified in terms of the rims of sectors of circles with subtending angle $2 \psi$ and inner and outer rim radii $r^{\min }$ and $r^{\max }$ respectively. Kernels become step-selection functions when located at a point (*x*, *y*), provided an angle of heading $\theta$ (**B**), placed on a landscape (**C**) with cells of different levels of attraction or repulsion (green, yellow, and white squares) and admissibility (all cells that overlap with the kernel), and associated with a step selection rule ${{\mathcal {R}}}$

In this paper, we focus on two dimensional models, which of course is only appropriate for some species (e.g., most terrestrial species) but not others (e.g., deep diving marine species or birds use thermals to gain height). For some birds, such as the barn owl data analyzed in Sect. 5.3 of this paper, accurate tracking in 3 dimension is infeasible and the height component of their flight is much smaller than the surface two dimensional components of their flight. Our model is thus implemented on a landscape represented by an $n^{\text{row}} \times n^{\text{col}}$ cellular array such that cell(*a*, *b*) has value $c_{ab,t}$ at time *t*: i.e.,

1 $$\begin{aligned} {{\mathcal {A}}}(t)=\big \{ c_{ab,t} \big | a=1,\cdots ,n^{\text{row}}, \ b=1,\cdots ,n^{\text{col}} \big \} \end{aligned}$$

Also, the topology of this array can be selected to be topology = torus (top-bottom and left-right continuity identification) or plane (top, bottom, left and right boundaries).

Each cell is identified both by its location (*a*, *b*) in the array, and by Euclidean coordinates $(x_a^{\text{cell}},y_b^{\text{cell}})$ at the lower left corner of cell(*a*, *b*). Depending on the units used to measure our Euclidean landscape, we define increments $\Delta x$ and $\Delta y$ such that $n^{\text{row}} \Delta x$ and $n^{\text{col}} \Delta y$ provide the desired dimensions for our landscape. Scaling is important when considering how far individuals are likely to move in one unit of time when in different movement modes and thus the scaling of time is linked to the scaling of space in real applications. In theoretical studies not linked to empirical data, however, it will be convenient to set $\Delta x=\Delta y=1$ and to set a parameter $\Omega _{\text{scale}}=10$ to scale space with respect to time such that the greatest distance an individual is likely to travel in one time step is given by $\Omega _{\text{scale}} \Delta x.$

The model simulates movement of an individual over this landscape, with one step executed at each tick *t* of the simulation clock for $t=0,\cdots ,n^{\text{time}}$. If an individual is at a point $(x_{t}^{\text{id}},y_t^{\text{id}})$ at time *t* and moves to cell(*a*, *b*), where its new location is now $(x_{\text{next}}^{\text{id}},y_{\text{next}}^{\text{id}}) = (x_a^{\text{cell}},y_b^{\text{cell}})$, then the distance moved, denoted as $\rho _{ab}(x_{t}^{\text{id}},y_t^{\text{id}})$ is defined as

2 $$\begin{aligned} \rho _{ab}(x_{t}^{\text{id}},y_t^{\text{id}})= \sqrt{ (x_a^{\text{cell}}-x_{t}^{\text{id}})^2 + (y_b^{\text{cell}}-y_t^{\text{id}})^2} \end{aligned}$$

The angle of heading, denoted as $\theta _{ab}(x_{t}^\text{id},y_t^{\text{id}}) \in [-\pi ,\pi ]$, as measured from the positive horizontal (i.e., the axis $x \ge 0$), is defined in terms of the so-called atan2 function as

3 $$\begin{aligned} \theta _{ab}(x_{t}^{\text{id}},y_t^{\text{id}})= \left\{ \begin{array}{cc} \arctan\, \Big (\frac{y_b^{\text{cell}}-y_t^{\text{id}}}{x_a^{\text{cell}}-x_{t}^{\text{id}}}\Big ) & \hbox {if }x_a^{\text{cell}}>x_{t}^{\text{id}} \\ \frac{\pi }{2} - \arctan\, \Big (\frac{x_a^{\text{cell}}-x_{t}^{\text{id}}}{y_b^{\text{cell}}-y_t^{\text{id}}}\Big ) & \hbox {if }y_b^{\text{cell}}>y_t^{\text{id}}\\ - \frac{\pi }{2} - \arctan\, \Big (\frac{x_a^{\text{cell}}-x_{t}^{\text{id}}}{y_b^{\text{cell}}-y_t^{\text{id}}}\Big ) & \hbox {if }y_b^{\text{cell}}<y_t^{\text{id}} \\ \arctan \Big (\frac{y_b^{\text{cell}}-y_t^{\text{id}}}{x_a^{\text{cell}}-x_{t}^{\text{id}}}\Big )\pm \pi & \hbox {if } x_a^{\text{cell}}<x_{t}^{\text{id}} \\ \hbox {undefined} & \hbox {if } x_a^{\text{cell}}=x_{t}^{\text{id}}\, \hbox {and}\, y_b^{\text{cell}}-y_t^{\text{id}} \end{array} \right. \end{aligned}$$

Whenever the angle of heading is reported as ranging on $[0,2\pi ]$, it should be transformed to range over $[\pi ,\pi ]$ to ensure that computations of the turning angle and its absolute values are computed correctly (see Eq. A.2 in the Appendix A.2, SOF).

Movement from current locations $(x_{t}^{\text{id}},y_t^{\text{id}})$ to a set of neighboring cells(*a*, *b*) is computed in terms of kernels $K_\alpha$, $\alpha =1,\cdots ,n^{\text{stame}}$ belong to a set ${{\mathcal {K}}}$, where each kernel $K_\alpha$ is defined as the rim of the sector of a circle centered on the origin, with rim dimensions $r^{\min }_\alpha$ and $r^{\max }_\alpha$ and sector angle $2 \psi _\alpha$ (Fig 2A) and includes an additional parameter $t^{\text{s}_{\alpha }}$ that influences the amount of time spent consecutively using kernel $K_\alpha$:

4 $$\begin{aligned} {{\mathcal {K}}}=\left\{ K_{\alpha }=K\left( r^{\min }_\alpha ,r^{\max }_\alpha , \psi _\alpha \right) \Big | \alpha =1,\cdots ,n^{\text{stame}} \right\} \end{aligned}$$

Of course, if $r^{\min }_\alpha =0$, then the rim is actually a sector (slice) of a circle of radius $r^{\max }$.

The kernels $K_{\alpha }$ become step-selection functions $\tilde{K}^{\alpha }_t$ when anchored at time *t* at a point $(x_{t}^\text{id},y_t^{\text{id}})$ with heading angle $\theta _t$ and ‘time spent in current movement mode’ variable $t^{\text{s}}_t$ (Fig 2B). They are associated with a set of movement rules ${{\mathcal {R}}}_{\lambda _\alpha }(\text{params})$, $\lambda _\alpha \in \{1,\cdots ,n^{\text{rule}}\}$, where “params” are any parameters in the procedure that it is convenient to highlight. In particular, params includes a switching parameter ${\hat{t}}^{\text{s}_\alpha }$ that controls the probability of switching out of the current kernel as a function of $t^{\text{s}_\alpha }$ and neighborhood cell value parameter ${\hat{c}}^{\text{nbh}}_\alpha$: i.e.,

5 $$\begin{aligned} \begin{aligned} {\tilde{K}}^{\alpha }_t\left( r^{\min }_\alpha ,r^{\max }_\alpha , \psi _{\alpha }; x,y,\theta , {{\mathcal {R}}}_{\lambda _\alpha }({\hat{t}}^{\text{s}_\alpha },{\hat{c}}^{\text{nbh}}_\alpha ) \right) = K_\alpha \hbox { at time }t, \hbox {anchored at} (x,y,\theta ) \hbox {and associated with } \\ \hbox {step-selection rule} {{\mathcal {R}}}_{\lambda _{\alpha }}({\hat{t}}^{\text{s}_\alpha },{\hat{c}}^{\text{nbh}}_\alpha ) \hbox {for some } \ \lambda _\alpha \in \{ 1,\cdots ,n^{\text{rule}} \}, \ \ \alpha =1,\cdots ,n^{\text{stame}} \end{aligned} \end{aligned}$$

### Landscape and individual dynamics

The initial set of landscape values ${{\mathcal {A}}}(0)$ (Eq. 1) can either be read in or generated using an algorithm that, for example, either assigns cell values at random or generates patches of high valued cells in a matrix of low-value cells and even barrier cells in more complex versions of the model than presented here. We have implemented an algorithm for constructing either of these two cases using three parameters and default starting values of $c_{ab,0}=0$ or 1: a parameter value $p^{\text{seed}}$ for laying down the first cells in patches at random, a parameter $p^{\text{cont}}$ for building patches into neighborhoods, and a parameter $n^{\text{cont}}$ for controlling the expected sizes of these patches. At the start of a simulation an individual animal has a state $h_0$, which is a representation of, for example, stored energy.

#### Patchy landscape generation

To initialize the landscape either read in an initial landscape file ${{\mathcal {A}}}(0)$ (Eq. 1) or select one of the following two algorithms designated as $\texttt {RAM}^{\text{patch}}_0$ and $\texttt {RAM}^{\text{patch}}_1$, following procedures laid out in the Section 4 (RAM is an acronym for Runtime Alternative Module and will be described in more detail later).

- *Randomly located regular patches* ($\texttt {RAM}^{\text{patch}}_0$)
  1. Default state: Set all cell values $c_{ab,0}=0$ for $a=1,\cdots ,n^{\text{rows}}$, $b=1,\cdots ,n^{\text{cols}}$
  2. Lay down an initial set of patch seeds by switching the value of cell(*a*, *b*) from $c_{ab,0}=0$ to $c_{ab,0}=1$ with probability $p^{\text{seed}}$ for $a=1,\cdots ,n^{\text{rows}}$, $b=1,\cdots ,n^{\text{cols}}$.
  3. For $a=1,\cdots ,n^{\text{rows}}$, $b=1,\cdots ,n^{\text{cols}}$ if $c_{ab,0}=1$ then switch all cells that lie within a Moore neighborhood of radius $n^{\text{cont}}$ to cell(*a*, *b*) to 1. This includes cells that may already have been switched to 1 because of their proximity to some other cell that has already been switched to 1.
- *Randomly located irregular patches* ($\texttt {RAM}^{\text{patch}}_1$)
  L.4 Default state: Set all cell values $c_{ab,0}=0$ for $a=1,\cdots ,n^{\text{rows}}$, $b=1,\cdots ,n^{\text{cols}}$
  L.5 First pass: Lay down an initial set of patch seeds by switching the value of cell(*a*, *b*) from $c_{ab,0}=0$ to $c_{ab,0}=1$ with probability $p^{\text{seed}}$ for $a=1,\cdots ,n^{\text{rows}}$, $b=1,\cdots ,n^{\text{cols}}$.
  L.6 Second pass: For $a=1,\cdots ,n^{\text{rows}}$, $b=1,\cdots ,n^{\text{cols}}$ if $c_{ab,0}=0$, and the sum of the values of cell(*a*, *b*)’s 4 neighbors (von Neumann neighborhood) is $\text{sum}_4\left( c_{ab}\right)$ then switch cell(*a*, *b*) from $c_{ab,0}=0$ to $c_{ab,0}=1$ with probability $1-\left( 1-p^{\text{cont}}\right) ^{{\text{sum}_4\left( c_{ab}\right) }}$
  L.7 Additional passes: Repeat step (c) $n^{\text{cont}}$ times (with the second pass corresponding to $n^{\text{cont}}=1$).

The the default for the initial value $h_0$ of an individual is set 10. Other values can be entered as discussed in the simulation parameter setup below.

We note that if $p^{\text{cont}}=0$, then cells in the array are randomly assigned a value 1 with probability $p^{\text{seed}}$

#### Dynamic updating

The cell array values $c_{ab,t}$ and individual value $h_{t}$ for $t=0,\cdots ,n^{\text{time}}$ are updated to account for the possibility that the individual gathers or extracts resources from cells as it moves over the landscape. This extraction may only take place during the implementation of some movement modes but not others.

Here we account for changes in these values as follows. The individual’s value $h_{t}$ changes over time as it acquires resources when occupying cells of value $c_{ab,t} > 0$. It also incurs a cost $\kappa ^{\text{sub}}$ per unit distance moved at each time step. Each time an individual occupies a cell(*a*, *b*) it removes some resources $f^{\text{remove}}$. If we assume that removal is a resource density independent process, with $f^\text{remove}=\min \{\kappa ^{\text{add}},c_{ab,t}\}$ then the following updating rules for the value-state of individuals (*h*) and cells (*c*) and parameters $\kappa ^{\text{add}}\in [0,1]$ and $\kappa ^\text{sub}$ apply: 

In moving from $(x_{t}^{\text{id}},y_t^\text{id})$at time* t* to cell we have the resource density-independent process

6 $$\begin{aligned} h_{\text{next}}= & \max \Big \{0,\ h_{t} + \min \big \{\kappa ^\text{add},\ c_{ab,t}\big \}- \kappa ^{\text{sub}} \rho _{ab}(x_{t}^\text{id},y_t^{\text{id}})\Big \} \nonumber \\ c_{ab,\text{next}}= & c_{ab,t} - \min \big \{\kappa ^{\text{add}},\ c_{ab,t}\big \} \end{aligned}$$

We can make this extraction dependent on the density of resource using the form of $f^{\text{remove}}=\frac{\kappa ^\text{add}c_{ab,t}}{\kappa ^{\text{add}}+c_{ab,t}}$, which is familiar to those who model consumer-resource interactions [54]. We can also allow for growth of the resource back to its carrying capacity of 1 at a a rate $\kappa ^{\text{grw}}$ when completely removed (e.g., for grasses this represents regrowth from an intact root-stock, etc). Finally we can also make the cost of travel size (energy value) dependent on a suitable scaling constant $\kappa ^{\text{scl}} \ge 1$ and multiplying $\kappa ^{\text{sub}} \rho _{ab}(x_{t}^{\text{id}},y_t^{\text{id}})$ by, for example, $\frac{1+h_t}{\kappa ^{\text{scl}}+h_t}$. In this case, as $\lim _{h_t \rightarrow \infty }\frac{h_t+1}{h_t + \kappa ^{\text{scl}}} \rightarrow 1$ and at $h_t=0$ we obtain the factor $\frac{1}{\kappa ^{\text{scl}}}<1$. In this case, we obtain the equations:

In moving from $(x_{t}^{\text{id}},y_t^\text{id})$at time* t* to cell (a,b), we have the resource density-independent process

7 $$\begin{aligned} h_{\text{next}}= & \max \Big \{0,\ h_{t} + \frac{\kappa ^{\text{add}}c_{ab,t}}{\kappa ^{\text{add}}+c_{ab,t}}- \left( \frac{h_t+1}{h_t + \kappa ^{\text{scl}}}\right) \kappa ^{\text{sub}} \rho _{ab}(x_{t}^{\text{id}},y_t^{\text{id}})\Big \} \nonumber \\ c_{ab,\text{next}}= & \min \left\{ 1,c_{ab,t} + \kappa ^{\text{grw}}\left( 1-c_{ab,t}\right) \right\} - \frac{\kappa ^{\text{add}}c_{ab,t}}{\kappa ^{\text{add}}+c_{ab,t}} \quad \hbox {for a currently occupied patch cell} (a,b) \nonumber \\ c_{ab,\text{next}}= & \min \left\{ 1,c_{ab,t} + \kappa ^{\text{grw}}\left( 1-c_{ab,t}\right) \right\} \hspace{2 cm} \quad \hbox {for all unoccupied patch cell} (a,b) \end{aligned}$$

We note that the simulation will stop either at $t=n^{\text{time}}$ or at $t^{\text{stop}}$ if $h(t^{\text{stop}})=0.$

In addition to the state value *h* of the individual, we will also keep track of the time $t^{\text{s}}_t$ it has spent in its current movement mode. Thus, we will update $t^{\text{s}}_t$ as follows

8 $$\begin{aligned} t^{\text{s}_\alpha }_{t+1} = \left\{ \begin{array}{cc} 0 & \qquad \hbox {every time the individual switches its movement mode from }\alpha ' { to}\alpha \\ t^{\text{s}_\alpha }_t + 1 & \hspace{-1.1cm} \text{ if } \text{ the } \text{ individual } \text{ remains } \text{ in } \text{ the } \text{ same } \text{ movement } \text{ mode } \alpha \end{array} \right. \end{aligned}$$

Thus at the start of the simulation, no matter the starting kernel, we initialize $t^{\text{s}}(0)=0$.

### Movement kernels

In our Numerus ANIMOVER_1 RAMP, we limit the implementation to at most two movement modes and hence two StaME kernels $K_{\alpha }$, $\alpha =\text{wp,bp}$. More specifically, we use $\alpha =\text {wp}$ to produce within-patch movement tracks and $\alpha = \text {bp}$ to produce between patch movement tracks. In addition, when $\alpha = \text{bp}$ we employ a vision kernel $K_\text{vis}(0,r^{\max }_{wp},\pi /2)$ which allows an individual to see patches peripherally and in front of it to the maximum radius of its next $\alpha =\text{bp}$ movement-mode step.

#### Kernel parameters

In general we expect within patch steps to be smaller than between patch steps, and within patch turning to be larger than between patch turning, although we have the flexibility to set up contrary scenarios. For each of the two movement modes at the start of a simulation, values for the triplets $\left( r^{\min }_\text{wp},r^{\max }_{\text{wp}}, \psi _{\text{bp}} \right)$ and $\left( r^{\min }_{\text{bp}},r^{\max }_{\text{bp}}, \psi _{\text{bp}} \right)$ are set up. If our expectation is followed, then we $r^{\max }_{\text{wp}}< r^{\max }_{\text{bp}}$ and $\psi _{\text{bp}} < \psi _{\text{wp}}$ will hold. We may also expect $r^{\min }_{\text{wp}}=0$ if the individual may choose to remain in the same cell for more than 1 time period and $r^{\max }_{\text{bp}}=r^{\min }+1\ \hbox {or}\ 2$ if within patch movements are considerably less than between patch movement where we suggest setting $r^{\max }_{\text{bp}}=10$. We again stress that the user has the flexibility to set and scale this values arbitrarily.

**Fig. 3:**
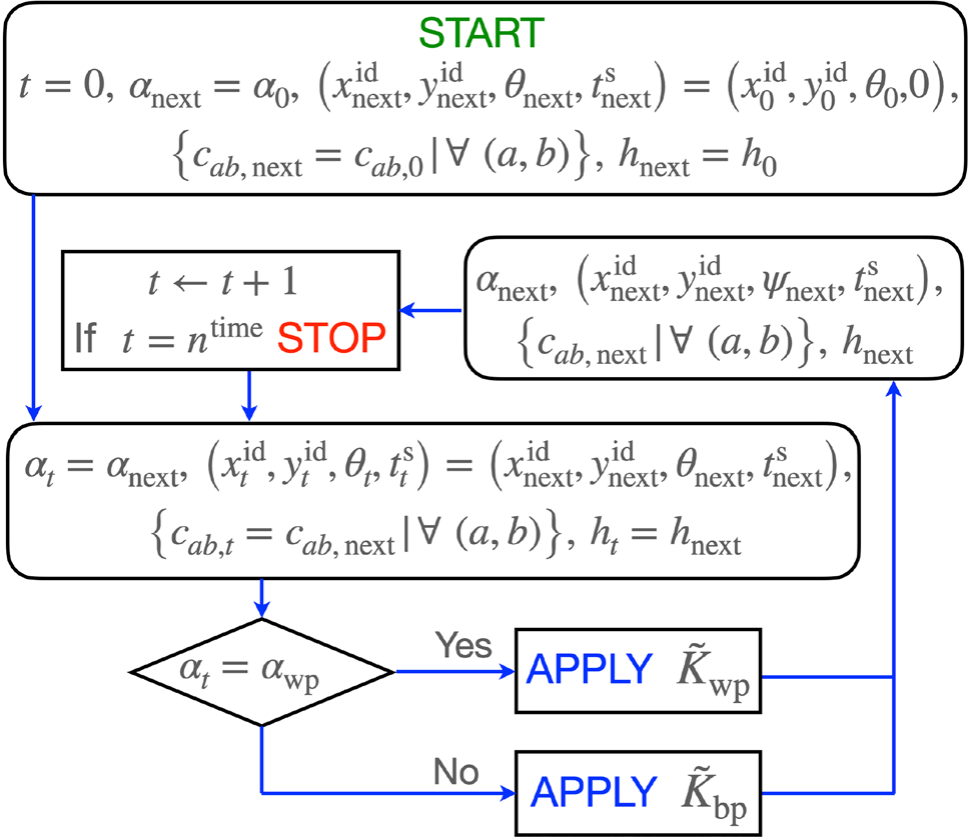
Algorithm used to simulate movement after the structural parameters $n^{\text{time}}$ (index *t*), $n^{\text{row}}$ (index *a*) and $n^{{{\text{col}}}}$ (index *b*) been entered, along with the topology of the landscape, the initial landscape values $\big \{c_{ab,0} | \forall (a,b) \big \}$, the initial state value $h_0$ of the individual, and the parameter triplets $\left( r^{\min }_{\text{wp}},r^{\max }_{\text{wp}}, \psi _{\text{wp}} \right)$ and $\left( r^{\min }_{\text{bp}},r^{\max }_\text{bp}, \psi _{\text{bp}} \right)$ that define the two kernels $K_{\text{wp}}$ and $K_{\text{bp}}$ respectively. Implementations ${\tilde{K}}^{\text{wp}}$ and ${\tilde{K}}^{\text{bp}}$ require the specification of step-selection procedures ${{\mathcal {R}}}_{\text{wp}}({\hat{t}}^{\text{s}_{\text{wp}}},{\hat{c}}^{\text{nbh}}_{\text{wp}})$ and ${{\mathcal {R}}}_{\text{bp}}({\hat{t}}^{\text{s}_{\text{bp}}},{\hat{c}}^{\text{nbh}}_{\text{bp}})$ respectively. The latter employs an additional kernel $K_{\text{vis}}$ that controls how the individual scans the landscape when it is moving between patches. The START, STOP, and APPLY commands, along with the flow arrows are show in different colors for additional clarity. The details of step selection procedures ${{\mathcal {R}}}_{\text{wp}}({\hat{t}}^{\text{s}_\text{wp}},{\hat{c}}^{\text{nbh}}_{\text{wp}})$ and ${{\mathcal {R}}}_{\text{bp}}({\hat{t}}^{\text{s}_{\text{bp}}},{\hat{c}}^{\text{nbh}}_{\text{bp}})$ can be found in Appendix A.3, SOF

All that remains in implementing the algorithm depicted in Fig 3, is specification of the parameters needed to implement our step-selection functions (SSFs, [55–57]) ${\tilde{K}}^{\text{wp}}$ and ${\tilde{K}}^{\text{wp}}$ using the rules ${{\mathcal {R}}}_{\lambda _{\text{wp}}}({\hat{t}}^{\text{s}_{\text{wp}}},{\hat{c}}^{\text{nbh}}_{\text{wp}})$ and ${{\mathcal {R}}}_{\lambda _{\text{bp}}}({\hat{t}}^{\text{s}_{\text{wp}}},{\hat{c}}^{\text{nbh}}_{\text{wp}})$ that are specified next (the last of these parameter arguments applies to $\texttt {RAM}_1^{\text{update}}$, but not $\texttt {RAM}_0^\text{update}$). We note that these procedures are coded as the default RAMs for our Numerus ANIMOVER_1 RAMP. Other procedures, possibly using integrated step selection analysis (iSSA, [58]) or methods that include direction-biasing external points of attraction or repulsion [59], may be included by the user.

#### Step-selection cells and probabilities

The step-selection rules ${{\mathcal {R}}}_{\lambda _\alpha }$ are procedures for updating the next location, angle of heading, and time spent in the current movement mode $\left( x_{\text{next}},y_{\text{next}},\theta _{\text{next}},t^{\text{s}}_\text{next}\right)$, as well as the kernel $\alpha _{\text{next}}$ to be used next. This is done in terms of the individuals current location, angle of heading and spent time $(x_{t}^{\text{id}},y_t^\text{id},\theta _t,t^{\text{s}}_t)$, and its current movement mode, as driven by the kernel implementation ${\tilde{K}}^{\alpha }_t$. In our case we have two sets of rules, one that specifies within patch movement (${{\mathcal {R}}}_{\text{wp}}({\hat{t}}^{\text{s}_{\text{wp}}},{\hat{c}}^\text{nbh}_{\text{wp}})$) and one that specifies between patch movement (${{\mathcal {R}}}_{\text{bp}}({\hat{t}}^{\text{s}_{\text{bp}}},{\hat{c}}^\text{nbh}_{\text{bp}})$). These rules include situations where the topology of the landscapes is a bounded rectangle and normal search fails to find a next location.

In computing our step-selection procedures we will make use of the set ${{\mathcal {C}}}^{\alpha }_t(x_{t}^{\text{id}},y_t^{\text{id}},\theta _t)$ defined as follows:

9 $$\begin{aligned} {{\mathcal {C}}}^{\alpha }_t(x_{t}^{\text{id}},y_t^{\text{id}},\theta _t)=\big \{ c_{ab,t} \big | \text{cell}(a,b) \hbox { overlaps with } \tilde{K}^{\alpha }_t \big \}, \ \ \alpha = \hbox {wp or bp} \end{aligned}$$

We will also make use of the following sets of probabilities

10 $$\begin{aligned} {{\mathcal {P}}}^{\alpha }_t(x_{t}^{\text{id}},y_t^\text{id},\theta _t)=\left\{ p_{ab} = \frac{c_{ab}}{ \sum _{\text{cell}(a,b) \in {{\mathcal {C}}}^{\alpha }_t}} \, \bigg |\, \forall \ \text{cell}(a,b) \in {{\mathcal {C}}}^{\alpha }_t \right\} \ \ \alpha = \hbox {wp or bp} \end{aligned}$$

Finally, we will make use of the probabilities $p_{\alpha }(t^\text{s})$ of continuing to use StaME $K_\alpha$ when having used this StaME for the past $t^{\text{s}}$ time steps

11 $$\begin{aligned} p_{\alpha }(t^{\text{s}}) = \frac{e^{-4\big (t^{\text{s}} - {\hat{t}}^{\text{s}_{\alpha }}\big )}}{1+e^{-4\big (t^{\text{s}} - {\hat{t}}^{\text{s}_{\alpha }}\big )}} \ \ \alpha = \hbox {wp or bp} \end{aligned}$$

An outline of the implementation of step selection procedures ${{\mathcal {R}}}_{\text{wp}}({\hat{t}}^{\text{s}_{\text{wp}}},{\hat{c}}^\text{nbh}_{\text{wp}})$ and ${{\mathcal {R}}}_{\text{bp}}({\hat{t}}^{\text{s}_\text{bp}},{\hat{c}}^{\text{nbh}}_{\text{bp}})$ is provided in Fig 3 with details provided in Appendix A.3 (SOF).

## Movement paths and StaME extraction

A movement track, whether simulated or empirical, in the first instance has a representation as a relocation time series of $n^\text{time}$ points—i.e., a walk

12 $$\begin{aligned} {W}= \bigl \{\bigl (t;x_t^{\text{id}}),y_t^{\text{id}}\bigr )\big |t=0,..., n^{\text{time}} \bigr \} \end{aligned}$$

Such tracks can be generated using simulations models, as we discus in some depth in the next section. In the case of empirical data, though, data preprocessing and filtering [60] are needed to get rid of spurious or problematic points or fill in missing points. From walk Eq. 12, both velocity and turning-angle time series can be generated, as outlined in Appendix A.2 (SOF) and elsewhere [30]. Various other statistics (e.g., travel distance, net displacement) can be extracted as well and used as variables to define a set of basic statistical movement elements (StaMEs), following methods described next.

### Creating StaMEs

The method described here to create StaMEs uses the velocity (*V*) and turning-angle ($\Delta \Theta$) time series derived from walk *W*, as described in Eq. A.1–A.4 (Appendix A.2, SOF). All points in our empirical time series that produced unrealistically large velocities were then removed. Although the putative maximum sustained flying speeds of barn owls have been observed in the range of 6–8 m/s (17.9 mph) [61, 62], to be conservative, we only removed points that represented unrealistically speeds. In our case, this amounted to a handful of points with velocities in excess of 75 m/s (168 mph); and we note that the average speed of the fastest 1 min segments in our analysis below (Table 2) turns out to be a credible $3 \hbox {-} 4$ m/s.

In the case of our simulated data, we normalized the entries of our cleaned *V* and $\Delta \Theta$ time series by dividing each of the entries $v_t$ in *V* by $v_{\max } = \max _{t=1,\cdots ,n^{\text{time}}} \{v_t \in V\}$ ($=10$ in our simulations) and dividing each of the entries in $\Delta \Theta$ by $2 \pi$ to obtain variables on the ranges [0, 1] and $[-1/2,1/2]$ respectively. In the case of our empirical data, we only normalized the turning angles since the velocities had the physical units m/s and we wanted to reflect this in our results.

**Fig. 4:**
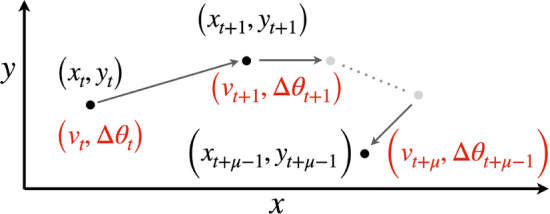
A relocation time series *W* (Eq. 12) is plotted in the *xy*-plan with computed velocities $v_t$ (Eq. A.2, SOF) and turning angles $\Delta \theta _t$ in red (Eq A.4) for a segment of $\mu$ points at times $\tau = t,t+1,\cdots ,t+\mu -1$. These values, or others (e.g., $v^{\text{per}}_t$ and $v^{\text{tan}}_t$; see Eq. A.5 and [18]) can then be used to compute statistical elements $\text{Seg}_{z}$ (Eq. 13) from a series of consecutive elements containing $\mu$ relocation points as, for example, in Eq. 13

Next, we parsed the normalized velocity and turning-angle time series into $z=1,\cdots ,n^{\text{seg}}$ segments each of length $\mu$ (Fig 4) with regard to *t*: in segments with missing points (these points may be filled in using an appropriate interpolation method) we kept the same length of segment and just adjusted for the reduced number of points (the errors from such missing points are likely to be inconsequential when the number missing is a few percent or less). The total number of segments so obtained was $n^{\text{seg}}=\lfloor \frac{n^{\text{time}}-1}{\mu } \rfloor$, with some points left over when $n^{\text{time}}-1$ was not exactly divisible by $\mu$.

We then calculated a set of statistics related to the $\mu$ normalized (in the simulated data only) velocities (equivalent to step lengths) and turning angles for each of the segments *z* in our time series data. Although various sets of statistics can be used (such as persistent and tangential velocities [18]), we settled upon mean velocities $V_z$ and mean absolute turning angles $| \Delta \Theta |_z$ for each segment and associated standard deviations $\text{SD}^{V}_z$ and $\text{SD}^{| \Delta \Theta |}_z$. Also to pick up any possible circular motion type biases in movement, we computed a normalized net displacement ($\Delta ^\rho$) statistic for each segment (i.e., the distance between the first and last points of each segment divided by quantity equal to the the number of points multiplied by the mean step-length). Specifically, for velocity and turning-angle means and standard deviations (SD) (normalized where appropriate), as well as net displacements, we defined a set of segments ${{\mathcal {S}}}_\mu$ (note below that $v_{\max } = 1$ for the empirical data and $\mu$ is adjusted for segments with missing points)

13 $$\begin{aligned} \begin{aligned} {{\mathcal {S}}}_\mu = \left\{ \text{Seg}_z \big (V_z, \text{SD}^{V}_z, | \Delta \Theta |_z, \text{SD}^{| \Delta \Theta |}_z, \Delta ^\rho _z \big ) \, \big | \, z = \lfloor \frac{t}{\mu } \rfloor +1, \ t=0,\cdots ,n^{\text{seg}}-1 \right\} \ \hbox { such that} \\ V_z=\frac{\sum _{\tau = t}^{t+\mu -1} v_{\tau }}{v_{\max } \mu } \ \text{ with } \text{ st. } \text{ dev. } \ \text{SD}^{V}_z, \ | \Delta \Theta |_z=\frac{\sum _{\tau = t}^{t+\mu -1} |\Delta \theta _{\tau }|}{2\pi \mu } \ \hbox { with st.~dev. } \ \text{SD}^{| \Delta \Theta |}_z \\ \hbox { and } \ \Delta ^\rho _z = \frac{\sqrt{(x_{t+\mu }^{\text{id}}-x_{t}^{\text{id}})^2 + (y_{t+\mu }^{\text{id}}-y_t^{\text{id}})^2 }}{\mu V_z} \end{aligned} \end{aligned}$$

### Mapping StaME centroids to kernels

We first applied our segmentation procedure Eq. 13 to data obtained from simulating the movement of an individual using a single kernel $K_\alpha = K(r^{\min }_\alpha , r^{\max }_\alpha , \psi _\alpha )$ on an unstructured (static, homogeneous) landscape. If one generates a segmentation set ${{\mathcal {S}}}_\mu$ (Eq. 13) from these simulation data, then using a variety of kernels with different admissible $r^{\min }_\alpha$, $r^{\max }_\alpha$ and $\psi _\alpha$ values, one can build up a discrete map represented by the function

14 $$\begin{aligned} {{\mathcal {F}}}^{\text{hom}}_\mu : \ \left( r^{\min }_\alpha , r^{\max }_\alpha ,\psi _\alpha , \right) \mapsto \big ({\overline{V}}_{\alpha }, \overline{\text{SD}^V}_{\alpha }, \overline{| \Delta \Theta |}_{\alpha }, \overline{\text{SD}^{| \Delta \Theta |}}_{\alpha }, \overline{\Delta ^\rho }_{\alpha } \big ) \quad \hbox {for all possible} K_\alpha \end{aligned}$$

We note that we have not subscripted the quantities in the image of this mapping with the parameter $\mu$. The reason is that the difference in values obtained for different values of $\mu$ are just variances associated with sampling and therefore are not consequential. However, we maintain the subscript $\mu$ on the mapping itself to remind ourselves that a value needs to be selected before this set can be generated. We also note, in the case of an unbiased walk, the statistic $\overline{\Delta ^\rho }$ may be ignored, because the realized movement has no prevailing circular bias to its motion. Under these circumstances, $\overline{\Delta ^\rho }$ is perfectly correlated with the other statistics defining each segment because clockwise and counterclockwise movements are equally likely. Additionally, values obtained should be relatively insensitive to $\mu$ when $\mu$ and the length $n^{\text{time}}$ of the track itself are sufficiently large to ensure that the law of large numbers is at play.

We then applied this segmentation procedure to data obtained from an ANIMOVER_1 two-kernel patch simulations ($K_{\text{wp}}$ and $K_\text{bp}$). Once the segmentation set ${{\mathcal {S}}}_\mu$ had been generated (Eq. 13), we carried out a hierarchical cluster analysis using Ward’s method (Appendix A.4, SOF) to obtain a set of *k* centroids represented by the set

15 $$\begin{aligned} {{\mathcal {X}}}^{\text{patch}}_{0,\mu ,k} = \Big \{ \big \{ \big ({\overline{V}}_\iota , \overline{\text{SD}^V}_{\iota }, \overline{| \Delta \Theta |}_\iota , \overline{\text{SD}^{| \Delta \Theta |}}_{\iota }, \overline{\Delta ^\rho }_{\iota } \big ) | \iota =1,\cdots ,k \big \}_{K_{\text{wp}},K_{\text{bp}}, {{\mathcal {L}}}^{\text{patch}}_0} \Big | \forall \hbox { combinations of } K_{\text{wp}},K_{\text{bp}}, {{\mathcal {L}}}^{\text{patch}}_0 \Big \} \end{aligned}$$

As in the case of quantifying selected points of the discrete map ${{\mathcal {F}}}^{\text{hom}}_\mu$, so can we quantify selected points of a discrete map ${{\mathcal {F}}}^{\text{patch}}_{0,\mu ,k}$ by computing *k* centroids in the set ${{\mathcal {X}}}^{\text{patch}}_{0,\mu ,k}$ from simulations of ANIMOVER_1 using different combinations of $K_\text{wp}$ and $K_{\text{bp}}$ kernels (i.e., step-selection procedures ${{\mathcal {R}}}_{\text{wp}}$ and ${{\mathcal {R}}}_{\text{bp}}$) with individuals moving over generated landscapes ${{\mathcal {L}}}^\text{patch}_0$. This mapping, in terms of the parameters used to generate it, can be expressed by:

16 $$\begin{aligned} {{\mathcal {F}}}^{\text{patch}}_{0,\mu ,k}: \left( K_{\text{wp}},K_{\text{bp}}, {{\mathcal {L}}}^{\text{patch}}_0\right) \mapsto {{\mathcal {X}}}^{\text{patch}}_{0,\mu ,k} \end{aligned}$$

In our computations, we selected our cluster number *k* to be 8 rather than 2, (see Appendix A.4, SOF, for details) even though we only had two movement modes. The reason for this is that some segments in our segmentation process will be mixtures of the two modes rather than homogeneous strings of points generated by either one or other of the two modes (see Fig C.1, Appendix C, SOF) while we expected only 2 of the 8 clusters to contain relatively homogeneous movement mode segments. Of course, the extent to which mixed versus pure movement mode segments arises depends both on the length of segments and the frequency at which the two movement modes switch between each another. The shorter the segments, or the less frequent the switching, the more likely any segment represent a series of locations generated by a single movement mode.

We can use our simulation model to numerically construct a mapping ${{\mathcal {F}}}^{\text{patch}}_{0,\mu ,k}$ of a set $\left( r^\text{min}_{\alpha },r^{\text{max}}_{\alpha }, \psi _{\alpha } \right)$ of kernel arguments onto a set of selected cluster centroid statistics $\big ({\overline{V}}_{\iota _{\alpha }}, \overline{\text{SD}^V}_{\iota _{\alpha }}, \overline{|\Delta \Theta |}_{\iota _{\alpha }}, \overline{\text{SD}^{|\Delta \Theta |}}_{\iota _{\alpha }}, \overline{\Delta ^\rho }_{\iota _{\alpha }} \big )$, as outlined in Appendix B (SOF). By way of illustration, we generate one point of the map ${{\mathcal {F}}}^{\text{hom}}_\mu$ in this paper. The range of values that may be useful to generate in a multi-point construction of this map will depend on the kind of empirical to which this mapping is fitted. Such an exercise is, thus, best left to a detailed study that explores the structure of given set of empirical movement data, such as an extended set of the owl data discussed below—a set containing data collected from at least several tens of individuals (e.g., as in [43, 52]).

## Numerus RAMPs

In the mid-2000’s s Grimm et al. [63] proposed an Overview, Design concepts and Details (ODD) protocol for the presentation of agent and individual-based models (ABMs and IBMs). A recent update of this protocol Grimm et al. [64] identifies 7 elements (3 overview, 1 design, and 3 description) needed to provide a coherent presentation of the study that conforms to the central tenant of science that “materials and methods must be specified in sufficient detail to allow replication of results Grimm et al. [64].” In Appendix D (SOF), we present an ODD protocol for the development of ANIMOVER_1, following Grimm et al. [64]’s ODD element numbering scheme.

We stress, though, that our study is about more than just building a simulation model to be used to simulate a known process using a currently available coding platform such as R or Python. In Sects. 2 and 3 we have presented a modeling framework that contains novel features regarding how concepts from step-selection function theory [55], when combined with a set of flexible rules that allow one to compute the probability of switching movement kernels (each of which represents a particular behavioral mode) in terms of local environmental factors, as well as internal clock variables, provides for the construction of a more versatile simulation model than, to the best of our knowledge, currently exists.

In the rest of this section, we present details that emphasize the utility, flexibility, and ease of use of our application platform ANIMOVER_1, based on its implementation of Numerus’ RAMP (runtime alterable model platform) technology and note that these RAMPs require not coding on behalf of the users unless they would like to change selected parts of that code pertaining generation of landscape patch structure or the functional forms of resource extraction from patches (see Sect. 4.2 for details). Although, ANIMOVER_1 itself is restricted to switching between two kernels that respectively implement within-patch (wp) and a between-patch (bp) movement modes, from the more general formulation of Sects. 2 and 3, it is clear that future versions of ANIMOVER_# can be developed that allow switching among many more modes of movement. It will also become clear in our presentation that although ANIMOVER_1 is not a general programming environment and does not require any coding experience to implement it, it does provide the user with considerable flexibility to implement sections of code pertaining to landscape patch structure initialization and to equations used to update the agent’s and landscape cells’ current states (i.e., the user may substitute the code in the runtime-alterable modules—RAMs—described below that implement Eqs. 6 or 7 for their own customized versions of these computations).

Finally, we emphasize that ANIMOVER_1 is a freely downloadable Numerus RAMP application that can be either be implemented using the Numerus Studio Platform, also downloadable for (links provided in Appendix C, SOF). We also note that Numerus RAMPs can be used in R programs as “virtual R packages,” as described at the Numerous RAMP webpage.

### RAMP construction

**Fig. 5:**
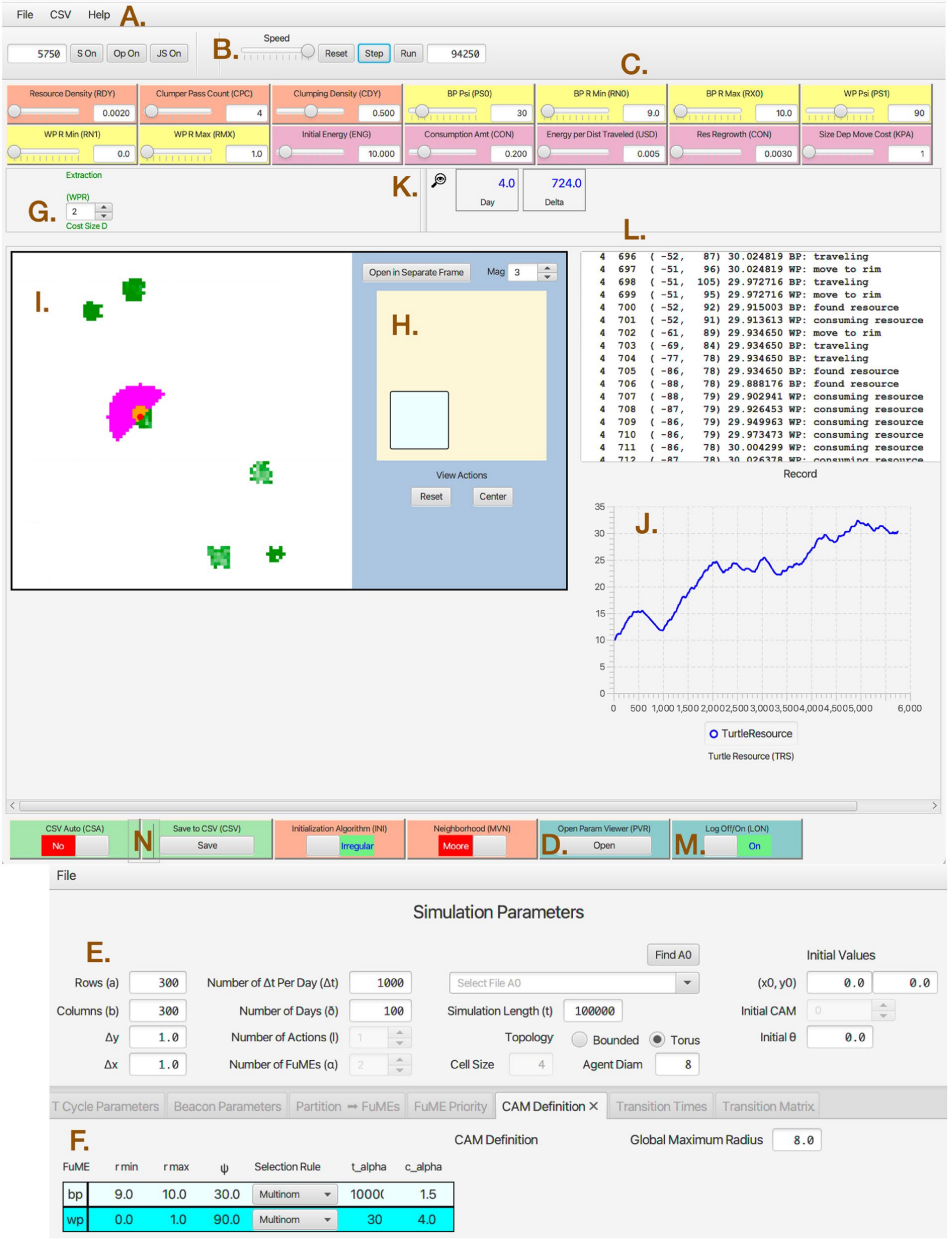
ANIMOVER_1 console as viewed in the Numerus Studio application. Labels **A**–**N** in brown are added for purposes of exposition. **A**: Pull down menus to load files. **B**: Run time controls allow for a reset in the middle or end of the run, step by step view of the changes (panels I and J), or automated run for full simulation time using parameters in panel **E** **C**: Color coded sliders used to change parameter values at the start of or during a run (orange: landscape initialization; yellow: kernel parameters; lilac: consumer resource interactions; green: saving output; turquoise: parameter and monitoring windows). **D:** Button to open the parameter windows **E** & **F**. **E**: A form where various parameters that control the size and scaling of the run are set. **F**: A set of forms to enter between (bp) and within (wp) patch kernel parameters (angles in degrees). **G**: The wheel is used to select the $\texttt {RAM}^{\text{update}}$ to be used. **H**: A viewer of the whole landscape with inset that can be observed as magnified in viewer I. **I**: A mouse-manipulable viewer of the landscape (panel **H**) that can be moved (using right click and move) and zoomed. Shown in I. are the current location (red dot), five patches (green pixels, lighter one previously exploited), and the current kernel (orange and lilac pixels). **J**: A plot of the value $h_t$ of the agent over time. **K**: Record of current day and within day iteration with additional **L**: activity log and **M**: log switch. **N**: Switches and buttons to save end-of-run output

The java-based Numerus Model Builder Designer (**NMB Designer**) Platform was used to code the model and then generate a **RAMP** (runtime alterable model platform) our **Numerus ANIMOVER_1** Application (with future versions 2, 3, etc. planned to include multidimensional landscapes, additional movement modes, and interacting individuals). The application takes the form of a portable file that, as described elsewhere [37, 65], and is played on the free downloadable NMB Studio application.

**Fig. 6:**
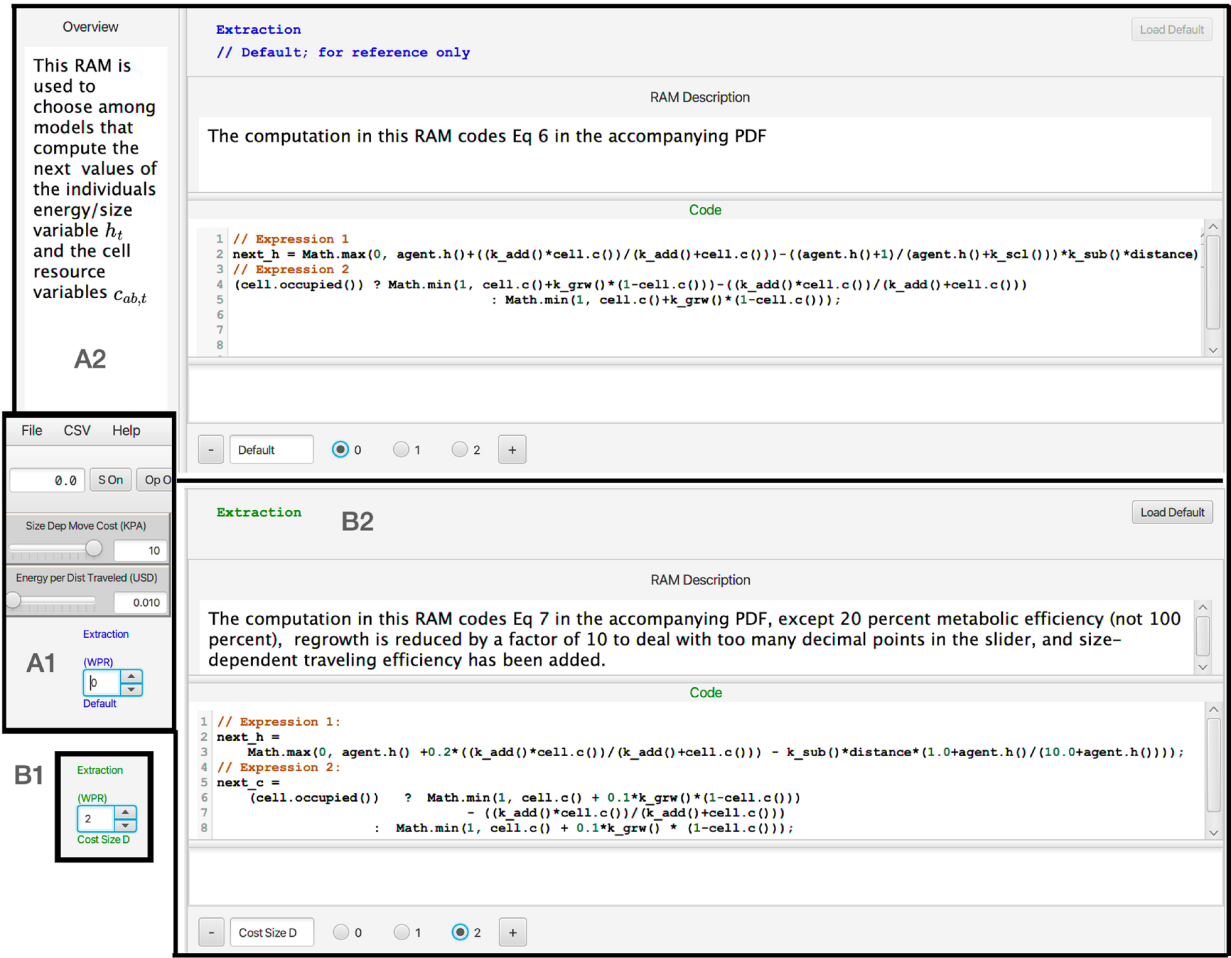
**A1**: The location on the ANIMOVER_1 console (**G**. in Fig 5) where the particular RAM to be implement in the current run is selected using the numbered roller. **A2**: The windows of the Default RAM showing the code that implements Eq. 6. **B1**: This shows the roller selected to position 2 to implement the Extraction RAM expressed in Eq. 7. Position 1 refers to a form of the equations not discussed in the text. **B2**: The windows of the Extraction RAM showing the code that implements Eq. 7. The “+” sign at the bottoms of windows A2 and B2 allow the user to open a window for the user to supply their own customized code for updating the state of the individual $h_t$ and cells array values $c_{ab,t}$

The flexibility of NMB RAMPs, beyond manipulating parameter values using its sliders (Fig 5E and F) and windows (Fig 5E and F), is facilitated by its runtime alterable modules (RAMs, Figs. 5G, and 6). These RAMs provide the user with the ability to chose alternative formulations of component parts of the model just priori to rerunning a current simulation or recode those component parts with alternate expressions. Thus, for example, in updating the value equations $h_t$ and $c_{ab,t}$, the RAMP uses the default $\texttt {RAM}^{\text{update}}_0$, which codes Eq. 6, as a default procedure, or the user can select $\texttt {RAM}^{\text{update}}_1$, which codes Eq. 7, as an alternate procedure (Fig. 6). In addition, the user can create a second alternative RAM by opening a new RAM window and inserting and saving code for customized equations, although parameters beyond the three already available as sliders at the console will have to be given fixed values (i.e., no new sliders can be created or added outside of upgrading the RAMP using the Numerus Designer Platform).

### RAMs

Two or more versions of the RAMs listed below exist: first a default version (RAM$_0$), a selectable alternative version (RAM$_1$), and perhaps additionally higher numbered versions or extemporaneously created versions, versions that can be added just prior to initiating a run of the model, and saved for future use. Thus, for example, Eq. 6 for the value dynamics of individuals and cells are coded up as default $\texttt {RAM}^{\text{update}}_0$, while the more elaborate density-dependent version of these equations (Eq. 7) are coded up as $\texttt {RAM}^\text{update}_1$.

ANIMOVER_1 includes the following RAMs:

- Initial landscape RAMs
- $\texttt {RAM}^{\text{patch}}_0$ (default) This RAM lays down a patchy landscape using rules L.1–L.3 to create irregularly-shaped (stochastic) patches stochastically placed at a specified density. It requires parameter values $p^{\text{seed}}$ to define the patch density, and $n^{\text{cont}}$ and $p^{\text{cont}}$ to generate the patch size and level of irregularity. Patches will be larger and more squarish when Moore is selected over von Neumann neighborhood.
- $\texttt {RAM}^{\text{patch}}_1$ (alt 1) This RAM lays down a patchy landscape using rules L.4–L.7 to create regularly shaped patches (cubes using Moore neighborhood, diamonds using von Neumann neighborhood) stochastically placed at a specified density. It requires parameter values $p^{\text{seed}}$ to define the patch density, and $n^{\text{cont}}$ to generate patch size.
- $\texttt {RAM}^{\text{patch}}_{2 \cdots }$ Available to the user for coding customized methods for creating initial landscape structures.
- Resource Extraction RAMs
- $\texttt {RAM}^{\text{val}}_0$ (default): This RAM updates the values $h_t$ and $c_{ab,t}$ using Eg 6 and requires parameter values $\kappa ^{\text{add}}$ and $\kappa ^{\text{sub}}$.
- $\texttt {RAM}^{\text{val}}_1$ (first alternative): This RAM updates the values $h_t$ and $c_{ab,t}$ using Eg 7 and requires parameter values $\kappa ^{\text{add}}$, $\kappa ^{\text{sub}}$ and $\kappa ^{\text{grw}}$.

### Parameter setup

The following set of parameters are needed to simulate the model. We also include some information on how our application console may be used to input some of the parameter values. Once this is done for those parameters that are either read in as a FILE (Fig 5A), entered prior to the simulation using a FORM (Fig 5E, F), specified using a PULLDOWN menu, or ignored—in which case DEFAULT values will be used, the Numerus ANIMOVER_1 will create a parameter values data file that can be download, edited and re-uploaded as needed. Other parameters will be entered and flexibly changed using a SLIDER (Fig 5C). Some of the these sliders will reflect values that are entered using a FORM while others will not be reflected in the form, but saved internally when the current simulation job is saved by the Numerus Studio Application Platform.

P.1 *Size and scope parameters* (FORMS). These are: $n^{\text{row}}$ (index *a*), $n^{\text{col}}$ (index *b*), $n^{\text{time}}$ (index *t*) and topology = torus or plane (PULLDOWN). We note that we have fixed $n^{\text{stame}}=2$ (index $\alpha$), Entry of these numbers and topology type respectively specify the dimensions of the cellular array, the length of the simulation, and whether the simulation takes place on a torus or a bounded plane.

P.2 ${{{Scaling}}\,{{parameters}}\, \Delta x, \Delta y}$ (FORMS or DEFAULT). Entry of these two Cartesian scaling values are used to assign the *x*-*y* coordinate values $(x_a^{\text{cell}},y_b^{\text{cell}})$ to each of the cells ($a=1,\cdots ,n^{\text{row}}$, $b=1,\cdots ,n^{\text{col}}$) in the simulation space ${{\mathcal {A}}}$. The default values for this are $\Delta x= \Delta y =1.$

P.3 ${{{Cellular}}\,{{array}}\, {{\mathcal {A}}}(0)}$ (FILE) or generating parameters $p^{\text{seed}}$, $p^{\text{cont}}$ and $n^{\text{cont}}$ (SLIDERS). This is an appropriately configured data file (e.g., csv text) that will up uploaded to the application to provide the initial state of all the cells in the simulation space or the initial landscape will be generated using the specified parameters algorithm L.2.

P.4 ${{{Kernel}}\,{{definition}} \,{{parameters}}\, {K_{\alpha }}, \alpha = {\text{wp and bp}}}$ (FORM and SLIDERS). Enter the three arguments for each of two kernels: i.e., $\left( r^{\min }_\alpha ,r^{\max }_\alpha , \psi _\alpha \right)$, $\alpha =$ wp and bp, respectively.

P.5 ${{{Kernel}}\,{{implementation}}\,{{parameters}}\,{{for}}\, {{\tilde{K}}^{\alpha }}}$ (SLIDER). Enter the arguments $\left( {\hat{t}}^{\text{s}_{\alpha }}, {\hat{c}}^{\text{nbd}}_\alpha \right)$ for the step-selection procedures ${{\mathcal {R}}}_{\alpha }$, $\alpha =$ wp and bp.

P.6 *Initial values* (various). The initial time is automatically taken to be $t=0$. The initial location and angle is computed from the selection of values (*a*, *b*) entered (FORM) or ignored (DEFAULT) and an initial value for the heading direction $\theta _0$ is entered (FORM) or ignore DEFAULT). The default starting cell is $a=\lfloor n^{\text{row}}/2\rfloor$, $b=\lfloor n^{\text{col}}/2\rfloor$, and default angle of heading is $\theta _0=0$. The actual starting location is thus $(x_a^{\text{cell}},y_b^{\text{cell}})$. The initial value $h_0$ for the individual must be entered (Form) or will be 10 (DEFAULT).

P.7 *Initial kernel wp or bp* (PULLDOWN). This will set the initial condition $\alpha _0$

P.8 ${{{Update}}\,{{parameters}}\,\kappa ^{\text{add}}, \kappa ^{\text{sub}}, \kappa ^{\text{grw}} {\text{and}} \kappa ^{\text{scl}}}$ (SLIDERS). The first two of these parameters are used in Eq. 6 or all four in Eq. 7 for updating $h_{t}$ and $c_{ab,t}$.

P.9 ${{{Step }}\,{{selection}}\,{{rule\,parameters}}\, {\hat{t}}^{\text{s}_{\text{wp}}}, {\hat{t}}^{\text{s}_{\text{bp}}}, {\hat{c}}_{ab,\text{wp}}, {\hat{c}}_{ab,\text{bp}}}$ (SLIDERS). The first two will be integer value sliders between [1, 1000], while the second two will be values to 1 dp between [0,5].

### Simulation setups and units

If landscape pixels are $\Delta x = \Delta y = 10$ m and $n^\text{row} = n^{\text{col}} = 500$ then our 250,000 pixel landscape is $5^2=25$ $\hbox {km}^2$. If the units of *t* are 1 m intervals, then in a day an individual will move up to $1440 = 60 \times 24$ times. If an individual walks at a persistent speed of 5 km per hour, which is $\frac{5000}{3600}=1.39$ m/s, then an individual can cross either the length or breadth of our landscape in 1 h, and a single pixel in 14 secs. In this case, the a maximum radius of $r^{\max } = 4$ would suffice, although a movement kernel covering short sprints would have a maximum radius of 4 or 6 times this value. For theoretical studies, boundary effects can be avoided during simulations by setting topology=torus (which makes the left and right columns of cells in the landscape array neighboring columns, and the top and bottom rows of cells neighboring rows).

### Model output

During the course of a simulation, one can visual observed the run as it progresses in window I (Fig 5) of the ANIMOVER_1 console. One can also observe the sequence of movements for the current day stage of the simulation in window L if switch M is in the “On” position (Fig 5). At the end of the run a CSV file is automatically saved if switch N is in the “Yes” position or if the “Save to CSV” button N is pressed after the run is complete. The saved CSV file is headed by a list of all the parameter settings for the run. It also has the following columns of data consecutive generated at step of the model simulation: day, within-day step, *x*-location, *y*-location, distance moved angle of head (degrees), agent-state (resources), movement mode (bp or wp) (Fig C.1, SOF). An example of this output can be found in Two_Kernel_Movement.csv (SOF) with a graphically depicted subset explained in Appendix C.1 (Output Data, SOF). In addition, links to downloading ANIMOVER_1 and to a RAMP Users Guide can be found in Appendix C.2 (SOF).

## Illustrative examples

### One point in the mapping of ${{\mathcal {F}}}_\mu ^{\text{hom}}$

By way of illustration, we used ANIMOVER_1 to construct 1 point in the mapping ${{\mathcal {F}}}_\mu ^{\text{hom}}(K)$ (Eq. 14). Specifically, we used the kernel $K(0,6,\pi /2)$ to simulate the movement of an individual for 1500 steps over a homogeneous landscape. Using a $\mu =15$ points segmentation interval we generated the set ${{\mathcal {S}}}_{15}$ (Eq. 13) and then calculated the means of the statistical variables of the interest to obtain $\left( {\overline{V}}, \overline{\text{SD}^V}, \overline{| \Delta \Theta |}, \overline{\text{SD}^{| \Delta \Theta |}}\right) = (3.38,1.23,0.27,27.6)$. Thus we identified one point in the mapping ${{\mathcal {F}}}^{\text{hom}}_\mu$ (Eq. 14): specifically,

17 $$\begin{aligned} {{\mathcal {F}}}^{\text{hom}}_{15}: \ \left( 0,5,\pi /2 \right) \mapsto (3.38,1.23,0.27,0.15) \end{aligned}$$

where we reiterate that we did not compute the displacement variable because it is non-informative due to the lack of a circular bias to the motion. Other points in the mapping can of course be constructed, as outlined in M.1–4 above. The range of parameters use to compute the structure of ${{\mathcal {F}}}^{\text{hom}}_\mu$ depends on the range of the image space $\left( {\overline{V}}, \overline{\text{SD}^V}, \overline{| \Delta \Theta |}, \overline{\text{SD}^{| \Delta \Theta |}}\right)$ that needs to be covered. Interpolation can also be used, where desired to estimate points that are contained within the nodes of the lattice structure used to compute the mapping at discrete points in the range space (as discussed in Appendix B, SOF).

### A two-movement mode simulation

We carried out a two-movement mode simulation on a patchy landscape that we manually stopped after 69,525 model steps. The parameters that we used for this simulation were:

- *Size and scaling* $n^{\text{row}}=300$, $n^{\text{col}}=300$, $n^{\text{time}}=100,000$ (as an upper limit) and topology = torus, $\Delta x=\Delta y=1$
- *Landscape generator* Initialization algorithm = Irregular, Neighborhood = Moore, $p^{\text{seed}}=0.1$ (resource density), $p^{\text{cont}}=0.7$ (clumping density) and $n^{\text{cont}}$ (clumper pass count)
- *Kernel parameters* Within patch: $r^{\min }_{\text{wp}}=0$, $r^{\max }_{\text{wp}}=1$, $\psi _{\text{wp}}=90,$ ${\hat{t}}^{\text{s}_\text{wp}}=30, {\hat{c}}^{\text{nbd}}_{\text{wp}}=1.5$; Between patch $r^{\min }_{\text{bp}}=9$, $r^{\max }_{\text{bp}}=10$, $\psi _{\text{bp}}=30,$ ${\hat{t}}^{\text{s}_{\text{bp}}}=10,000, {\hat{c}}^{\text{nbd}}_{\text{bp}}=4$
- *Update parameters* $\kappa ^{\text{add}}=0.4$, $\kappa ^\text{sub}=0$, $\kappa ^{\text{grw}}=0.03$ and $\kappa ^{\text{scl}}=1.0$

**Fig. 7:**
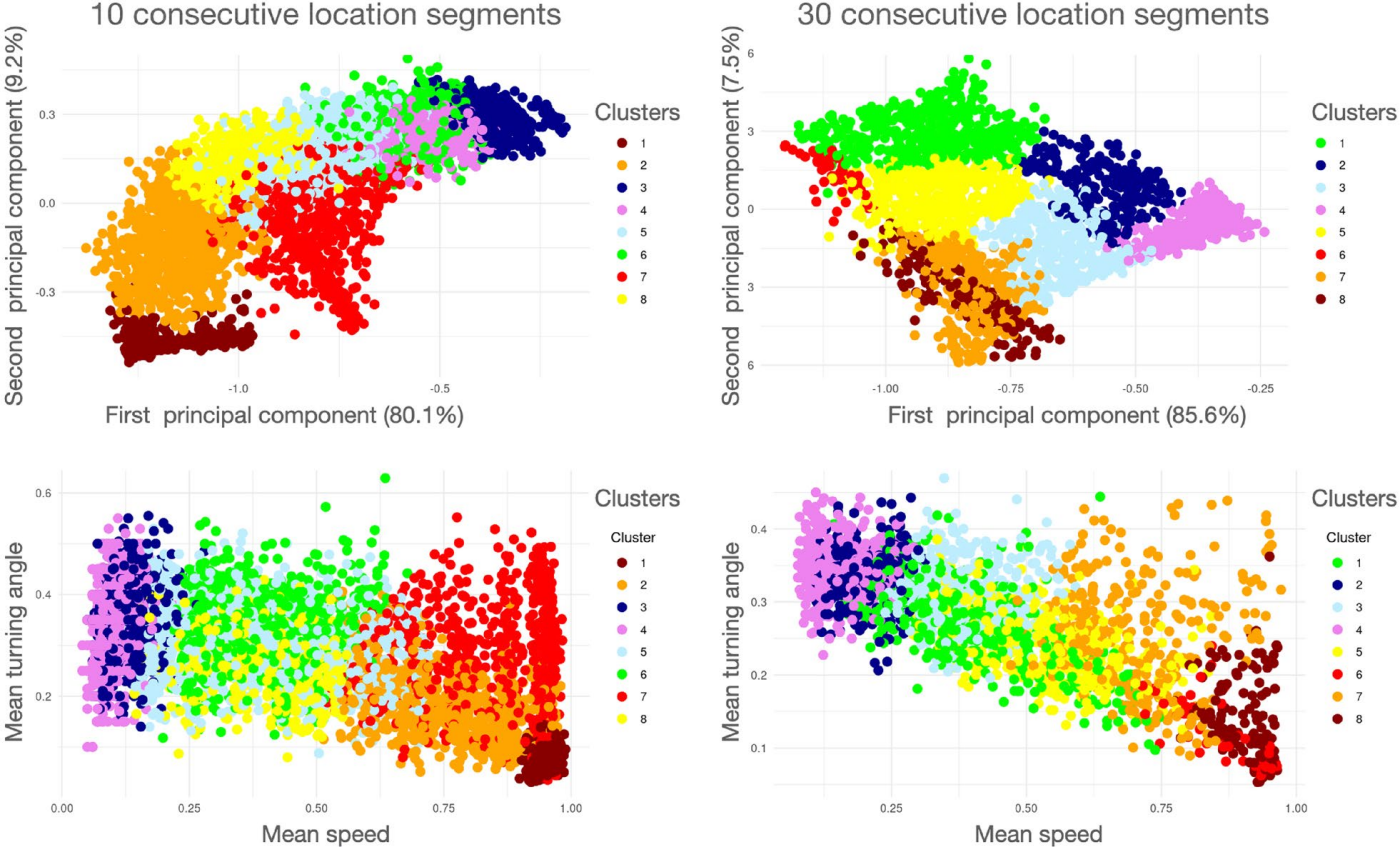
Results of a hierarchical cluster analysis (with number of clusters $k=8$) performed on both 10-point (upper and lower left panels, $\nu =10$; see Fig 4) and 30-point (upper and lower right panels, $\nu =30$) segmentation of the simulation data generated with parameters specified for our two movement mode simulation (also see Fig C.1, Appendix C, SOF). The two top panels are plots of the clusters in PC1/PC2 space and the two lower panels are plots in Mean-speed/Mean-turning-angle space. A color spectrum is used to depict the smallest (blue end) to largest (red end) of segments within clusters. The centroid arguments of each cluster (Eq. 15) are listed in Table 1. The colors have been selected to reflect a spectral scale of the largest to smallest mean speed for each cluster’s centroid, though the cluster numbers (Column C in Table 1) are set by the clustering algorithm. Depictions of 130 randomly selected segments from each cluster for the 10-step segmentation case can be seen in Figs A.2–A.9 (Appendix A, SOF)

**Table 1:** The centroid arguments (Eq. 15) obtained from a hierarchical cluster analysis (with $k=8$ clusters labeled $\text{C}=1,\cdots ,8$, see legends in Fig 7) analysis of 10- and 30-step segmentation of our two movement mode simulation output

C	# Segs. (%)	${\overline{V}}\pm {\overline{\text{SD}^V}}^\dag$	$\overline{| \Delta \Theta |}\pm {\overline{\text{SD}^{| \Delta \Theta |}}}^\dag$	$\overline{\Delta ^\rho }\pm {\text{SD}^{\overline{\Delta ^\rho }}}^\ddag$	Prop. wp
			$${{\bf 10-step\, segs}}^*$$		
1	811 (11.7)	0.95$\pm 0.04$	0.08$\pm 0.05$	0.86$\pm 0.14$	0.01$\pm 0.04$
7	694 (10.0)	0.84$\pm 0.21$	0.28$\pm 0.25$	0.14$\pm 0.11$	0.51$\pm 0.10$
2	776 (11.2)	0.75$\pm 0.33$	0.18$\pm 0.18$	0.79$\pm 0.13$	0.27$\pm 0.15$
8	546 (7.9)	0.38$\pm 0.40$	0.27$\pm 0.23$	0.81$\pm 0.08$	0.66$\pm 0.16$
6	838 (12.1)	0.38$\pm 0.38$	0.33$\pm 0.25$	0.11$\pm 0.06$	0.75$\pm 0.10$
5	1057 (15.2)	0.37$\pm 0.36$	0.31$\pm 0.23$	0.46$\pm 0.14$	0.71$\pm 0.17$
3	987 (14.2)	0.12$\pm 0.10$	0.35$\pm 0.23$	0.18$\pm 0.07$	0.90$\pm 0.10$
4	1243 (17.9)	0.10$\pm 0.07$	0.34$\pm 0.23$	0.44$\pm 0.12$	0.93$\pm 0.08$
			$${{\bf 30-step\, segs}}^*$$		
8	138 (6.0)	0.91$\pm 0.15$	0.14$\pm 0.14$	0.27$\pm 0.14$	0.18$\pm 0.17$
6	51 (2.2)	0.86$\pm 0.20$	0.12$\pm 0.12$	0.82$\pm 0.09$	0.12$\pm 0.10$
7	306 (13.2)	0.71$\pm 0.36$	0.25$\pm 0.24$	0.17$\pm 0.12$	0.48$\pm 0.16$
5	416 (18.0)	0.53$\pm 0.41$	0.25$\pm 0.23$	0.49$\pm 0.09$	0.52$\pm 0.13$
1	463 (20.0)	0.40$\pm 0.39$	0.27$\pm 0.23$	0.70$\pm 0.10$	0.63$\pm 0.15$
3	361 (15.6)	0.39$\pm 0.39$	0.31$\pm 0.25$	0.14$\pm 0.10$	0.74$\pm 0.07$
2	246 (10.6)	0.20$\pm 0.26$	0.33$\pm 0.23$	0.29$\pm 0.11$	0.85$\pm 0.06$
4	336 (14.5)	0.15$\pm 0.18$	0.35$\pm 0.23$	0.08$\pm 0.05$	0.89$\pm 0.06$

$^*$All numbers are dimensionless due to normalizations (see text for details)

$^\dag$This is the average of the standard deviations reported for each segment in the cluster

$^\ddag$This is the standard deviation of the normalized net displacement across all segments in the cluster

The column “Prop. wp” lists the mean and standard deviation of the proportion of with-in to between patch kernels (i.e. $\frac{\text{wp}}{\mathrm{wp + bp}}$; see Fig C.1, Appendix C, SOF) averaged across all segments in each cluster. The results are listed in descending size of average speed $({\bar{V}})$ for the centroid of each cluster. Depictions of 130 randomly selected segments from each cluster for the 10-step segmentation case can be seen in Figs A.2–A.9 (Appendix A, SOF)

Under pure between-patch (bp) movement on a homogeneous landscape, given that $r^{\min }_{\text{bp}}=9$ and $r^{\max }_{\text{bp}}=10$, we should expect ${\overline{V}}_{\text{bp}}$ after normalizing by $V_\text{bp} \approx 10$ to be in the range [0.9, 1]. Also given that the turning should, on average (since $\psi _{\text{wp}}=30$ degrees) be around $(0.5 \times 30)/180 \approx 0.083$. We carried out such a simulation over 100,000 steps and obtained ${\overline{V}}=0.95$, $\overline{\text{SD}^V}=0.03$, $\overline{| \Delta \Theta |}=0.08$, $\overline{\text{SD}^{| \Delta \Theta |}}=0.05$, and $\overline{\Delta ^\rho }=0.83$, which are almost identical to the values for the 10-step segmentation of the data that has the largest ${\overline{V}}$ value or fastest speed as reported in the Table (i.e., cluster C = 1 for which ${\overline{V}}=0.95$, $\overline{\text{SD}^V}=0.04$, $\overline{| \Delta \Theta |}=0.08$, $\overline{\text{SD}^{| \Delta \Theta |}}=0.05$, and $\overline{\Delta ^\rho }=0.86$). Thus the segments in this cluster from our two movement mode simulation are very close to what we obtain when we simulate pure one-mode between-patch movement. The cluster with the largest ${\bar{V}}$ in our 30-step segmentation (cluster C = 8) does not fit nearly as well, presumably because many fewer pure bp movement segments arise in this case compared with the 10-step segmentation case.

This last conclusion is reinforced by looking at the mean proportion of wp kernel steps associated with the segments of the different clusters reported in right-most column of Table 1. Thus only 1% of the 811 cluster 1 (largest ${\overline{V}}$) segments of length 10 steps were generated by a wp kernel, while the mean proportion of wp kernel steps associated with the two largest ${\overline{V}}$ 30-step segments were clusters 8 and 6 with reported proportions 0.12 and 0.18 in clusters 6 and 8 respectively. At the other end of the velocity spectrum, the two smallest velocity 10-step segment clusters 3 and 4 reported a mean of 0.90 and 0.93 wp segments, while in the 30-step segment case the two smallest velocity clusters 2 and 4 reported a mean of 0.85 and 0.89 wp segments respectively. This result reinforces our earlier comment and obvious result (see Fig C.1, Appendix C, SOF) that segments with fewer steps provide a greater proportion of pure step-type StaMEs than those based on more steps.

The remaining seven 10-step segment clusters and six 30-step segment clusters contained segments that included mixtures of wp and bp, ranging from averages of a quarter to three quarters of each type in the segments of each cluster. This calls into question what the optimal number of base segment clusters and, hence number of StaME types should be. For example, to what extent should we merge clusters reporting similar segment statistics? The results reported in Table 1 suggest that for the 10-step segmentation perhaps clusters 3 and 4 could be combined, although they are well-separated by their mean relative net-displacement value ($\overline{\Delta ^\rho }=0.18$ versus 0.44 respectively). This is true of many clusters in the 30-step case. For example, ignoring the value of $\overline{\Delta ^\rho }$ suggests that clusters 6 + 8, 5 + 1 + 3, and 2 + 4 might be combined to yield four distinct clusters, but in each case the variable $\overline{\Delta ^\rho }$ provides some cause for separation. In general, however, the optimal number of StaMEs will be data dependent. Additionally, finding the optimal number requires that we develop suitable measures so that such questions can be answered with some rigor. Such measures are currently being developed in the context of an information theory formulation of CAM construction using StaMEs as a set of building block segments [34].

A random selection of 130 segments from each of the 8 clusters in the case of the 10-step segmentation are plotted in Figs A.2–A.9 (Appendix A, SOF, where we note that the actual size of segments across panels is not comparable because the axes in each panel have been automatically set by our plotting routines. From these illustrations it is clear that the fastest cluster ($\text{C}=1$; Fig A.2, Appendix A, SOF) consist primarily of unidirectional lines, as is characteristic of between-patch movement, with some small deviations and occasional changes in direction. This is in contrast with the smallest cluster ($\text{C}=4$; Fig A.5) that has a number of segments switching directions by $\pi /2$ every couple of steps, as is characteristic of in-patch foraging. The difference between segments $\text{C}=6$ (Fig A.7) and $\text{C}=8$ (Fig A.9), which have virtually the same average speeds (Table 1) and similar average turning angles look quite different: the start and end points are relatively close with spiky profiles ($\text{C}=6$) or far apart with much more open profiles ($\text{C}=8$).

### Empirical data example

**Fig. 8:**
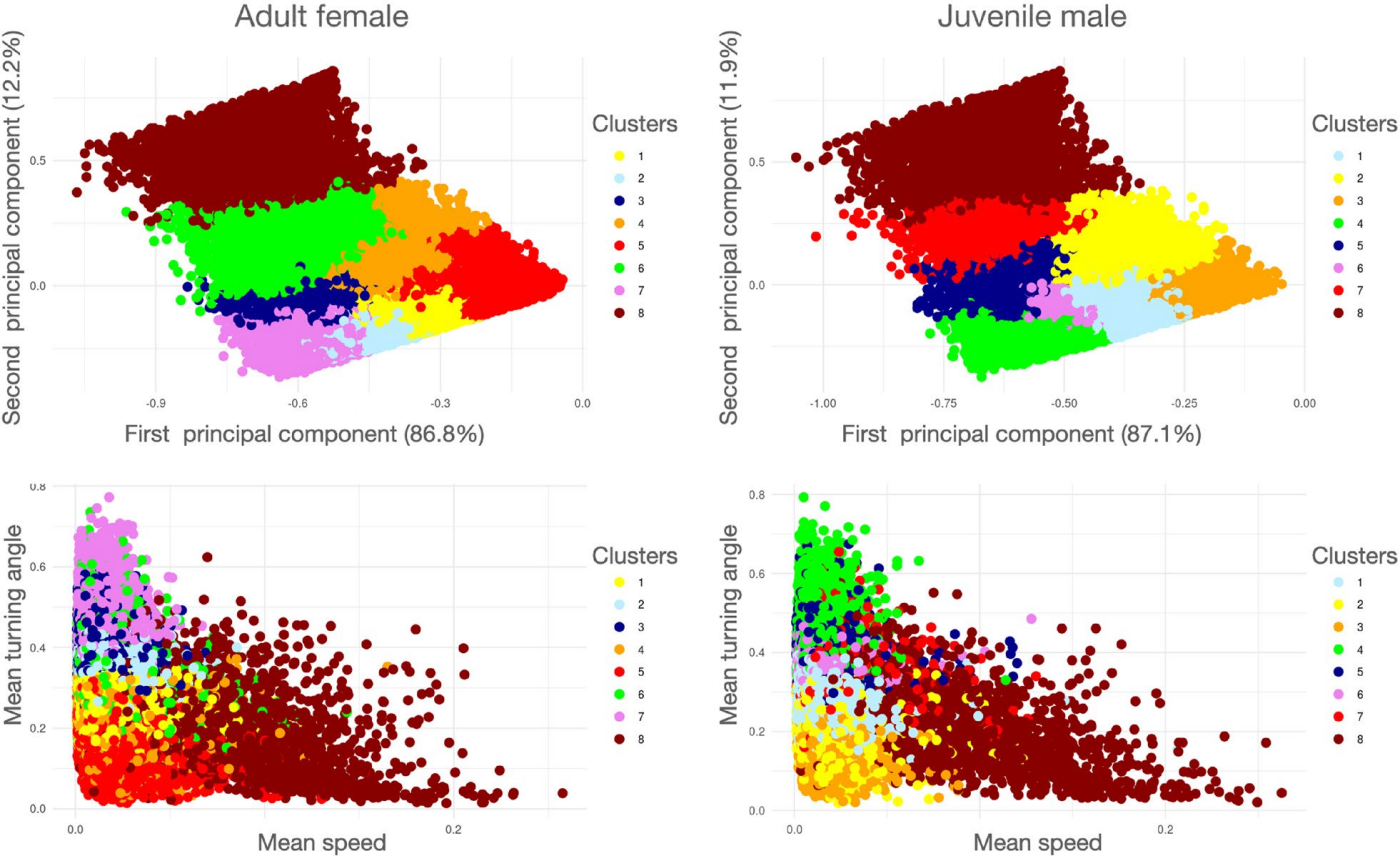
Results of cluster analysis ($k=8$) performed on segmentation of the tracks of two different barn owls. As in Fig 8 The two top panels are plots of the clusters in PC1/PC2 space and the two lower panels are plots in Mean-speed/Mean-turning-angle space. A color spectrum is used to depict the smallest (blue end) to largest (red end) of segments within clusters. The centroid arguments of each cluster (Eq. 15) are listed in Table 2. The colors have been selected to reflect a spectral scale of the largest to smallest mean speed for each cluster’s centroid, though the cluster numbers (Column C in Table 2) are set by the clustering algorithm. Note that mean speed, as plotted here is 10 times smaller than the mean speeds ${\bar{V}}$ recorded in Table 2: this resizing was made to avoid extra zeros after the decimal points the in the Table. Also note that the triangular distribution of points in bottom two graphs arises because smaller turning angles are associated with the occurrence of segments with faster average speeds. Depictions of 130 randomly selected segments from each cluster for the Adult female and Juvenile male segmentation cases can be seen in Appendix A (SOF) in Figs A.10–A.17 and Figs A.18–A.25 respectively

**Table 2:** The centroid arguments (Eq. 15) obtained from a hierarchical cluster analysis (with $k=8$ clusters labeled $\text{C}=1,\cdots ,8$, see legends in Fig 7) analysis of a segmentation of tracks from two different barn owls

**C**	Segments	${\overline{V}}\pm {\overline{\text{SD}^V}}^\dag$	$\overline{| \Delta \Theta |}\pm {\overline{\text{SD}^{| \Delta \Theta |}}^\dag }$	$\overline{\Delta ^\rho }\pm {\text{SD}^{\overline{\Delta ^\rho }}}^\ddag$
	# (%)	(m/s)	(units $\pi$)	(proportion)
*Adult female*				
8	4530 (5.2)	2.80$\pm 1.81$	0.24$\pm 0.23$	0.79$\pm 0.12$
5	11655 (13.3)	$1.07\pm 0.68$	0.18$\pm 0.15$	0.13$\pm 0.06$
4	12670 (14.4)	$0.79\pm 0.52$	$0.25\pm 0.22$	0.27$\pm 0.06$
1	19641 (22.4)	0.76$\pm 0.47$	0.27$\pm 0.25$	0.10$\pm 0.04$
6	9278 (10.6)	0.71$\pm 0.54$	0.33$\pm 0.28$	0.42$\pm 0.08$
2	11639 (13.3)	0.69$\pm 0.42$	0.36$\pm 0.31$	0.73$\pm 0.03$
3	10450 (11.9)	0.61$\pm 0.38$	0.39$\pm 0.30$	0.22$\pm 0.04$
7	7895 (9.0)	0.61$\pm 0.36$	0.46$\pm 0.31$	0.12$\pm 0.05$
*Juvenile male*				
8	3622 (5.8)	2.55$\pm 1.75$	0.24$\pm 0.23$	0.77$\pm 0.12$
7	2679 (4.3)	0.76$\pm 0.64$	0.35$\pm 0.28$	0.49$\pm 0.06$
3	3553 (5.7	0.72$\pm 0.45$	$0.16\pm 0.15$	0.09$\pm 0.04$
2	7730 (12.4)	0.59$\pm 0.40$	0.24$\pm 0.21$	0.28$\pm 0.07$
4	9446 (15.1)	0.51$\pm 0.31$	0.44$\pm 0.32$	0.08$\pm 0.04$
1	21029 (33.6)	0.50$\pm 0.30$	0.28$\pm 0.24$	0.12$\pm 0.06$
5	5956 (9.5)	0.50$\pm 0.33$	0.41$\pm 0.30$	0.29$\pm 0.06$
6	8484 (13.6)	0.46$\pm 0.27$	0.38$\pm 0.29$	0.16$\pm 0.05$

$^\dag$This is the average of the standard deviations reported for each segment in the cluster

$^\ddag$This is the standard deviation of the normalized net displacement across all segments in the cluster

The results are listed in descending size of average speed $({\bar{V}})$ (m/s) for the centroid of each cluster. The turning angles are proportions of $\pi$, and the displacement are proportions as described in the methods. Depictions of 130 randomly selected segments from each cluster for the Adult female and Juvenile male segmentation cases can be seen in Appendix A (SOF) Figs. A.10–A.17 and Figs. A.18–A.25 respectively

We analyzed relocation data obtained from two barn owls (*Tyto alba*), using an ATLAS reverse GPS technology system that was set up in the Harod valley in northeast Israel [52]. The relocation data for both individuals, an adult female and a juvenile male (GG41259 and GG41269 in the original data set and individuals with IDs 29 and 31 in [43]), were collected a frequency of 0.25 Hz (i.e., one point every 4 s) during a several week period in the late summer of 2021. We used a 15-step segmentation, which corresponds to segmenting the movement track into one minute sequences. We then performed a hierarchical cluster analysis with 8 clusters, described in Appendix A.4 (SOF) and obtained the results illustrated in Fig 8. The centroid statistics obtained for each cluster are provided in Table 2. Unlike the simulated results were we normalized the velocities to lie between 0 and 1, the velocities in Table 2 have the units of meters per second. Also, recall that the absolute values of average turning angles ($\overline{\Delta \Theta }$) are proportions of $\pi$ while the net displacements ($\overline{\Delta ^\rho }$) are the distances between the start and end points of each segment as a proportion of the sum of the lengths of the 15 consecutive steps that make up each segment.

We see from Table 2 that the average speeds of the cluster of fastest (i.e., largest) segments for the adult female and juvenile male (C = 8 in both cases) are 2.80 and 2.55 m/s respectively. For both individuals, we see in Table 2 that these “fastest clusters” (as represented by ${\bar{V}}$) are around 3 times faster than the next fastest clusters (compare first and second rows of values in Table 2), with the fastest clusters containing only 5–6% of all segments. For both individuals the bulk of the segments have speeds that lie around the geometric means of the fastest and slowest clusters of segments (i.e., between 0.35$-$0.8 m/s), are very similar in size but these intermediate speed clusters highly variable in their shape: some are relatively open (say, net displacement ${\bar{\Delta }}^\rho \ge 0.3$) and some are relatively closed (say, net displacement ${\bar{\Delta }}^\rho <0.2$). The slowest clusters of the adult female and juvenile male have the relatively low speeds of 0.10 and 0.15 m/s respectively, but these slowest adult female segments are much more open on average ($\text{C}=7$, $\bar{\Delta \rho }=0.44$) than the juvenile male segments ($\text{C}=6$, $\bar{\Delta \rho }=0.08$). The reason for this requires a much closer look at the locations of these segments on the actual landscape where they occurred.

Depictions of 130 randomly selected segments from each cluster for the Adult female and Juvenile male segmentation cases can be seen in Figs A.10-A.17 and Figs 18-A.25 (Appendix A, SOF) respectively. These depictions cannot be compared across segments for size because the axes in each panel of each figure have been automatically generated by our plotting routines. They also do not provide any landscape details. Such considerations remain a subject for future studies once analyses of the movement tracks of multiple individuals of each type have been undertaken.

As somewhat expected, a distinct correlation is evident between the mean speed and mean turning angle of a segment. This expresses itself through the lower triangular distribution of points in the mean-speed/mean-turning-angle plots in Fig 8. This correlation also appears in the simulated data as well, but it is much less obvious: it is indicated by the negative slope of a band of points (Fig 7) rather than by a triangular distribution of points. The band implies that segments with lower speed in the simulation data have larger turning angles, while in the empirical data, lower speed still allows for a range of turning angles.

## Discussion

The information that can be extracted from the movement path of an individual is akin to decoding a string of highly degraded symbols without an accompanying Rosetta stone to interpret the meaning of these symbols. In fact, interesting albeit superficial commonalities and differences can be drawn between reading a book and “reading” the life-time track of an animal’s story from its birth to its death. In a book, pages are spatially well-defined objects, as are the temporally well-defined diel activity routines of the lifetime track of an animal (Fig 1). Canonical activity mode (CAM) segments within a behavioral activity mode (BAM) are like words in a sentence, while BAMs within a diel activity routine (DAR) are like the sentences on a page. Unlike a book, however, where every letter is clearly visible, the letters (FuMEs) of an animal track are not at all visible, only hints of letters at regularly “spaced” (in time) intervals. Thus, instead of being able to see the words as we can in any book, we can only conjure up poor images of ersatz words in the form of StaMEs (Statistical Movement Elements, Fig 1). The book metaphor for lifetime tracks, however, is too simple in one very important way: books start with blank pages upon which letters are then printed. Tracks are generally imposed upon highly structured rather than blank landscape and the underlying landscape structure plays a decisive role in determining the spatiotemporal characteristics of the tracks that are imprinted on it. [66–68].

In some ways, our Rosetta stone is our simulation model. It allows us through simulations to see how different kernels produce segments with particular sets of statistics. We illustrated this in Sect. 5.1 where we showed, for example, that a kernel with minimum- and maximum-step-length and range-of-turning-angle parameters values $r^{\min }=0$, $r^{\max }=5$, and $\psi =\pi /2$ creates a cluster of segments whose centroid has mean step-length and turning-angles (standard deviations in parenthesis) of 3.38 (±1.23) and 0.27$\pi$ (±0.15$\pi$) (Eq. 17).

More ambitiously, we could numerically construct 2-patch mappings, as defined in Eq. 16, where Table 2 represents the set of centroids ${{\mathcal {X}}}^{\text{patch}}_{0,\mu ,k}$ (Eq. 15) obtained under a hierarchical 8-cluster, $\mu =15$ point-segmentation of the simulated data (using $\texttt {RAM}^{\text{patch}}_0$) when the arguments of this mapping are (see Eq. 17) the simulation parameters listed in Sect. 5.2. In the case of trying to match movement modes to empirical relocation data, we can use the actual landscape associated with those data, plus set selection functions fitted to different parts of the landscape (e.g., movement within and between areas where particular types of activities take place). This appears to be a challenging process where it remains to be seen in future studies how the methods discussed in this paper can be applied to identifying putative movement modes that appear to be used by individuals when moving across real landscapes and carrying out a range of activities.

When comparing the diversity of the clusters obtained from the adult female (AF) and juvenile male (JM) barn owl tracks, as reported in Table 2, with that of the simulated data, as reported in Table 1, its clear that our StaME approach has the potential to match real with simulated data. For example, when using 10-step segments the range of values is nearly 10-fold for velocity, 4 to 5-fold for absolute turning-angle, and 8-fold for relative net-displacement. For the 30-step segments these fall to 6-fold for velocity, 3-fold for absolute turning-angle, and 6-fold for relative net-displacement. Comparable ranges for the barn owl are around 5-fold for velocity (somewhat less for the AF and more for the JM), around 3-fold for absolute turning-angle, and 8–10 fold for net displacement (lower end for AF, upper end of JM). Thus our simulations show a greater velocity range and a greater absolute turning-angle range, but a smaller relative net-displacement range than our barn owl data. The larger barn owl net-displacement range arises because the owls typically return home each day, but displacement to another resting site occurs on some days [43]. To capture this behavior in our ANIMOVER_1 simulator, we would need to add a movement kernel that is biased to move in the direction of a homing beacon at certain times during the diel cycle.

The challenge should not be underestimated when it comes to demonstrating how a bottom-up StaME approach to constructing and analyzing movement track structures, when combined with fitting auto-regressive models (AR(p) where p is the depth of the time delay dependence), can be used to fit models to movement patterns at the subdiel and diel scales. Stochastic differential equation (SDE) methods have typically been used to estimate macro level quantities emerging from the movement behavior of individuals and populations, such as home range, speed, and distance travelled [69, 70]. To keep things simple, however, these methods avoid the complexities of dynamic background environments, which are known to greatly influence movement behavior [71]. Bottom up hierarchical modeling approaches, however, by linking movement to environment using step-selection functions [55], provide a direct way of incorporating environmental covariates into movement behavior. This behavior is complicated by the fact that an individual’s internal state variables (motivations linked to time-of-day and time-of-year factors, hunger, thirst, fear, etc.) are also critically important [72] Ultimately these factors will need to be included in simulation models used to predict the movement patterns of individuals at the level of diel and subdiel movement patterns. Incorporation of these, which remains a challenge for future studies, will require fitting auto regressive models to the sequencing of CAMs with movement mode switching probabilities that are functions of all critical covariates that individuals use to decide on where to move next as their movement track relocation time series unfolds under various environmental conditions.

Of course, other approaches to simulating movement have been developed, several of which are mathematically more sophisticated than the approach we take. Some of these treat movement as continuous-time SDE process for which Brownian motion (purely random movement) and Orstein-Uhlenbeck processes (directionally correlated random walks) and modifications thereto (e.g., velocity correlated walks, central attractors, etc.) [73–77] are examples. Others represent movement in terms of partial differential equations some random components that switch between gradient following search (advection plus random noise) and random search [78] or discrete approaches using integrated step-selection functions [79]. In addition, machine learning methods have been shown to outperform SDE models for predicting the next location of individuals in some systems [80].

It is worth noting that spatio-temporally continuous models are from a computational point of view also discrete in requiring discretization schemes to generate numerical solutions. In such cases, however, the aim is to keep the discretization small enough and solution methods robust enough so the associated discretization errors do not affect interpretations of outcomes. Formulating movement models discretely, as we have, with regards to space and time avoids the issue of what is an appropriate solution method, but introduces both philosophical and practical issues regarding differences in discrete- versus continuous-time representations of ecological processes [81], particularly since data always discretized in one way or another [82]. Additionally, discrete time, space, and variable trait formulations of ecological processes facilitate the incorporation of idiosyncratic details that elude the more rigorous formulation of continuous variable formulations. This is particularly true when it comes to turning model formulations into computational code for analysis through numerical simulation. Such formulations are naturally facilitated when movement is embedded into network structures [83, 84] or is modeled as taking place on cellular arrays [85–88].

Given the complex shapes of the segments plotted in Figs A.2-A.25 (Appendix A, SOF), the hierarchical clustering approach that we have taken to parsing the movement track segments into categories may not be the best approach. Supervised and unsupervised deep-learning approaches provide less forced and more powerful ways to extract clusters, particularly those targeted at time series data [89–94]. In terms of supervised, deep-learning approaches, convolutional neural nets (CNNs) of appropriate types may be trained using segments simulated by ANIMOVER_1 over real landscapes, obtained through the application of various sets of movement rules, as a way of generating the training sets [95]. Once trained these CNNs can then be used to classify empirical data segments thereby providing some insights into the types of movement rules that may have been used by actual individuals when moving over the landscapes in question. Further, we note that rectangular looking elements illustrated in Figs A.4 and A.5 are a function of the scale of the discretized landscapes over which trajectories are simulated or, for that matter analyzed, in terms of the smallest step size invoked or time interval used to record consecutive locations. However, no matter what scale is selected, the smallest StaMEs—i.e., those associated with resting behavior—will be limited by both the level of discretization used in simulating data or the errors associated with measuring locations using GPS or reverse GPS technologies [96]. In addition, the scale of discretization is ultimately limited by the consistency/stability trade-off of numerical computations [97]. It should also be added that various measures can be taken to correct errors associated with the measurement of locations using techniques to smooth out data or apply Kalman filtering, but this may lead to some loss of temporal resolution in the underlying data (e.g.,see [21]).

## Conclusion

In the absence of suitable high frequency data, insights into the ecological underpinnings of the movement tracks are currently being obtained by studying them the hierarchical level of behavioral activity modes (BAMs), [21], diel activity routines (DARs) [42, 43, 98] and above [99, 100]. The reason for this is its fixed temporal length of 24 h, which makes it possible to segment in a relatively unambiguous way. The only ambiguity is what point in time during the diel period should be fixed as the start/end point for each DAR. This problem has been discussed elsewhere [101, 102]. Additionally, DARs can be strung together to obtain supra-diel constructs (Fig 1) such as the extent of seasonal range of individuals [32, 103], and beyond this to classifying the syndromic movement behavior from whole or abbreviated lifetime tracks [100].

Next steps to follow on from the work presented here likely include studies to evaluate the most appropriate clustering methods to be used to classify segmentation types at different levels of segmentation (e.g., StaMEs to LiTs, Fig 1). It should also include studies of how a bottom-up StaME approach to constructing and analyzing movement track structures, when combined with fitting auto-regressive models (AR(*p*) where *p* is the depth of the time delay dependence), can be used to fit models to higher order segments, particularly variable length BAMs and how this compares with the more direct approach of identifying BAMs using behavioral change point analysis (BCPA) and hidden Markov models (HMM).

Over the next decade we can expect exponential increases over time in the availability of high-frequency movement relocation time series for many different species, reflecting both growing interest in the topic, and technological improvements in tracking methods. This increase, together with the increasing computational power of server clusters, the use of parallel processing, and improvements and extensions to our ANIMOVER_1 simulator will make the methods discussed in this paper for deconstructing diel activity routine tracks into StaMEs more reliable and easier to implement. Additionally, deep learning methods and possibly some other machine learning methods [91–95, 104] that are able to account for complexities in the shapes of the segments arising from our analysis (e.g., see Figs A.2-A.25 in Appendix A, SOF) may be more useful for categorizing segments than the hierarchical clustering approach used here. It will also facilitate using StaMEs to bring movement canonical activity modes (CAMs) and higher level behavioral activity modes (BAMs) or movement syndromes [32, 105] into sharper focus using various time series forecasting techniques [106, 107] and more sophisticated movement simulation algorithms.

### Glossary

For the convenience of the reader we provide a glossary of indices and symbols in Table 3.

**Table 3:** Indices and symbols used to formulate the model’s structure

Symbols	Explanation	Ref.
*Indices and periods*		
$a=1,\cdots ,n^{\text{row}}$	Row and column indices to denote landscape cell(*a*, *b*)	Eq. 1
$$b=1,\cdots ,n^{\text{col}}$$		
$t=0,\cdots ,n^{\text{time}}$, $t^{\text{stop}}$	Index and number of time steps in global simulation, stop time if agent value drops to 0	Fig. 3
$\mu$, $z=1,\cdots ,n^{\text{seg}}$	Number of consecutive points in a StaME segment, index for and number of segments in a DAR	Fig. 4
$\alpha =1,\cdots ,n^{\text{stame}}$	StaME index and number	Eq. 4
$\lambda =1,\cdots ,n^{\text{rule}}$, ${{\mathcal {R}}}_\lambda$, ${{\mathcal {R}}}_{\lambda _\alpha }$	Rule index and number, step-selection rules, $\alpha$-specific rule	Eq. 5
$\iota ,\cdots ,k$	Cluster index (iota) and number of clusters	Eq. 15
*Structures*		
$(x_a^{\text{cell}},y_b^{\text{cell}})$, $\Delta x,$ $\Delta y$, $\Omega _{\text{scale}}$	Locations of cell(*a*, *b*) on the landscape, row and column distances between consecutive cells, and the time-space scaling constant	
${{\mathcal {A}}}(t)$, $c_{ab,t}$, $(x_{t}^{\text{id}},y_t^{\text{id}})$, $\theta _t$	The state of cellular array ${{\mathcal {A}}}$, of cell$_{ab}$, and location and angle of heading of the agent at time *t*	Eq. 1
$p^{\text{seed}}$, $p^{\text{cont}}$, $n^{\text{cont}}$	Parameters used to generate a patchy landscape	$\texttt {RAM}^{\text{patch}}$
$\rho _{ab}(x_{t}^{\text{id}},y_t^{\text{id}})$, $\theta _{ab}(x_{t}^{\text{id}},y_t^{\text{id}})$	Distance between the individuals current location and a cell $(x_a^{\text{cell}},y_b^{\text{cell}}$) and angle of heading to this cell	Eq. 2-3
$K_{\alpha }$, ${{\mathcal {K}}}$, $r^{\mathrm{\min }}_\alpha ,\ r^{\mathrm{\max }}_\alpha ,\ \psi _\alpha$	StaME kernels, kernel $\alpha$ and its min & max SL (step length, aka velocity), and TA (turning-angle) range arguments	Eq. 4
${\tilde{K}}^{\alpha }_t(\text{args})$, $\theta _t$ ${{\mathcal {R}}}_{\lambda _\alpha }$, ${\hat{t}}^{\text{s}_\alpha }$, ${\hat{c}}^{\text{nbh}}_\alpha$	Movement kernel $\alpha$ at time *t* and angle of heading angle of heading, step-selection rule, mode switching and neighborhood-value parameters	Eq. 5
$h_t$, $t^{\text{s}_\alpha }_{t}$, $h_{\text{next}}$, $c_{cb,\mathrm next}$	Agent value at time *t*, time spent in current movement mode $\alpha$, agent and cell interim values	Eq. 6-8
$\kappa ^{\text{add}}$, $\kappa ^{\text{sub}}$, $\kappa ^{\text{grw}}$, $\kappa ^{\text{scl}}$	Parameter values used in cell and agent updating equations	Eqs. 6 and 7
${{\mathcal {C}}}^{\alpha }_{t}({x_{t}^{\text{id}},y_t^{\text{id}},\theta _t})$	The set of all cells that overlap with kernel ${\tilde{K}}^{\alpha }_t(\text{args})$	Eq. 9
${{\mathcal {P}}}^{\alpha }_{t}({x_{t}^{\text{id}},y_t^{\text{id}},\theta _t})$	The set of probability values for cells that overlap with kernel ${\tilde{K}}^{\alpha }_t(\text{args})$	Eq. 10
$p_{\alpha }(t_s)$, ${\hat{t}}^{\text{s}_\alpha }$	Probability that an individual in mode $\alpha$ for period $t^{\text{s}_\alpha }$ continues in this mode, the $\alpha$ mode switching function parameter value	Eq. 11
*W*, *V*, $\Delta \Theta$	Stochastic walk, velocity (aka step length:SL) and turning-angle (TA) relocation time series	Eqs. 12, A.1–A.3
$\text{Seg}_z$, ${{\mathcal {S}}}_\mu$, $V_z$, $\text{SD}^{V}_z$, $| \Delta \Theta |_z$, $\text{SD}^{| \Delta \Theta |}_z$, $\Delta ^\rho _z$	Segment *z*, set of segments, segment specific average velocity and standard deviation, average turning angle and standard deviation, net displacement	Eq 13
${{\mathcal {F}}}^{\text{hom}}_\mu$	Mapping of the parameters of kernel $K_{\alpha }$ onto the centroid of all segments generated by a walk using this kernel on a homogeneous landscape	Eq. 14
${{\mathcal {X}}}^{patch}_{0,\mu ,k}$, ${{\mathcal {F}}}^{patch}_{0,\mu ,k}(\text{args})$	Set of centroids from a hierarchical cluster analysis of a 2-mode patchy landscape simulation using $\texttt {RAM}^{\text{patch}}_0$, and resulting discrete mapping from the relevant parameter space to the centroid space	Eq. 15-16

## Supplementary Information

Supplementary file 1.

Supplementary file 2.

## Data Availability

The simulation data can be found in the supplementary online file Two_Kernel_Movement.csv. The data used for the barn owl analysis is available at the Github repository https://github.com/LudovicaLV/DAR_project, as provided in [43]. ANIMOVER_1 (deployed as the RAMP file Ani1Cr3.nms, see Appendix C, SOF) and the most recent release of Numerus Studio can be downloaded without cost from the Numerus webpage https://www.numerusinc.com/studio/. Instructions for using Numerus Studio are contained in the RAMP Users Guide at https://wiki.numerusinc.com/index.php/Ramp_User_Guide. Numerus RAMPs can be used in R computing environments as virtual R packages.

## References

[1] Dougherty ER, Seidel DP, Carlson CJ, Spiegel O, Getz WM. Going through the motions: incorporating movement analyses into disease research. Ecol Lett. 2018;21(4):588–604. 29446237 10.1111/ele.12917

[2] Saltz D, Getz WM. Finding a home: stopping theory and its application to home range establishment in a novel environment. Front Conserv Sci. 2021;2:714580.

[3] Abrahms B, Aikens EO, Armstrong JB, Deacy WW, Kauffman MJ, Merkle JA. Emerging perspectives on resource tracking and animal movement ecology. Trends Ecol Evol. 2021;36(4):308–20. 33229137 10.1016/j.tree.2020.10.018

[4] Allen AM, Singh NJ. Linking movement ecology with wildlife management and conservation. Front Ecol Evol. 2016;3:155.

[5] Berger-Tal O, Saltz D. Invisible barriers: anthropogenic impacts on inter-and intra-specific interactions as drivers of landscape-independent fragmentation. Philos Trans R Soc B. 2019;374(1781):20180049. 10.1098/rstb.2018.0049 PMC6710564 31352896

[6] Binning SA, Shaw AK, Roche DG. Parasites and host performance: incorporating infection into our understanding of animal movement. Integr Comp Biol. 2017;57(2):267–80. 28859405 10.1093/icb/icx024

[7] Shaw AK. Causes and consequences of individual variation in animal movement. Mov Ecol. 2020;8(1):12. 32099656 10.1186/s40462-020-0197-x PMC7027015

[8] Armstrong JB, Takimoto G, Schindler DE, Hayes MM, Kauffman MJ. Resource waves: phenological diversity enhances foraging opportunities for mobile consumers. Ecology. 2016;97(5):1099–112. 27349088 10.1890/15-0554.1

[9] Thorup K, Tøttrup AP, Willemoes M, Klaassen RHG, Strandberg R, Vega ML, Dasari HP, Araújo MB, Wikelski M, Rahbek C. Resource tracking within and across continents in long-distance bird migrants. Sci Adv. 2017;3(1): e1601360. 28070557 10.1126/sciadv.1601360 PMC5214581

[10] Abrahms B, Hazen EL, Aikens EO, Savoca MS, Goldbogen JA, Bograd SJ, Jacox MG, Irvine LM, Palacios DM, Mate BR. Memory and resource tracking drive blue whale migrations. Proc Natl Acad Sci. 2019;116(12):5582–7. 30804188 10.1073/pnas.1819031116 PMC6431148

[11] Morales JM, Moorcroft PR, Matthiopoulos J, Frair JL, Kie JG, Powell RA, Merrill EH, Haydon DT. Building the bridge between animal movement and population dynamics. Philos Trans R Soc B Biol Sci. 2010;365(1550):2289–301. 10.1098/rstb.2010.0082 PMC2894961 20566505

[12] Hays GC, Bailey H, Bograd SJ, Don Bowen W, Campagna C, Carmichael RH, Casale P, Chiaradia A, Costa DP, Cuevas E, et al. Translating marine animal tracking data into conservation policy and management. Trends Ecol Evol. 2019;34(5):459–73. 30879872 10.1016/j.tree.2019.01.009

[13] Sokolow SH, Nova N, Pepin KM, Peel AJ, Pulliam JRC, Manlove K, Cross PC, Becker DJ, Plowright RK, McCallum H, et al. Ecological interventions to prevent and manage zoonotic pathogen spillover. Philos Trans R Soc B. 2019;374(1782):20180342. 10.1098/rstb.2018.0342 PMC6711299 31401951

[14] Ims RA. Movement patterns related to spatial structures. In: Mosaic landscapes and ecological processes. Cham: Springer; 1995. p. 85–109.

[15] Morales JM, Haydon DT, Frair J, Holsinger KE, Fryxell JM. Extracting more out of relocation data: building movement models as mixtures of random walks. Ecology. 2004;85(9):2436–45.

[16] Getz WM, Saltz D. A framework for generating and analyzing movement paths on ecological landscapes. Proc Natl Acad Sci. 2008;105(49):19066–71. 19060192 10.1073/pnas.0801732105 PMC2614716

[17] Edelhoff H, Signer J, Balkenhol N. Path segmentation for beginners: an overview of current methods for detecting changes in animal movement patterns. Mov Ecol. 2016;4(1):21. 27595001 10.1186/s40462-016-0086-5 PMC5010771

[18] Gurarie E, Andrews RD, Laidre KL. A novel method for identifying behavioural changes in animal movement data. Ecol Lett. 2009;12(5):395–408. 19379134 10.1111/j.1461-0248.2009.01293.x

[19] Chen J, Gupta AK. Parametric statistical change point analysis: with applications to genetics, medicine, and finance. Cham: Springer; 2011.

[20] Gurarie E, Bracis C, Delgado M, Meckley TD, Kojola I, Michael Wagner C. What is the animal doing? tools for exploring behavioural structure in animal movements. J Anim Ecol. 2016;85(1):69–84. 25907267 10.1111/1365-2656.12379

[21] Teimouri M, Indahl U, Sickel H, Tveite H. Deriving animal movement behaviors using movement parameters extracted from location data. ISPRS Int J Geo-Inf. 2018;7:78. 10.3390/ijgi7020078.

[22] Gundermann KP, Diefenbach DR, Walter WD, Corondi AM, Banfield JE, Wallingford BD, Stainbrook DP, Rosenberry CS, Buderman FE. Change-point models for identifying behavioral transitions in wild animals. Mov Ecol. 2023;11(1):65. 37864238 10.1186/s40462-023-00430-0 PMC10589947

[23] Thompson PR, Harrington PD, Mallory CD, Lele SR, Bayne EM, Derocher AE, Edwards MA, Campbell M, Lewis MA. Simultaneous estimation of the temporal and spatial extent of animal migration using step lengths and turning angles. Mov Ecol. 2024;12(1):1. 38191509 10.1186/s40462-023-00444-8 PMC10775566

[24] Franke A, Caelli T, Hudson RJ. Analysis of movements and behavior of caribou (*Rangifer tarandus*) using hidden Markov models. Ecol Model. 2004;173(2–3):259–70.

[25] Langrock R, King R, Matthiopoulos J, Thomas L, Fortin D, Morales JM. Flexible and practical modeling of animal telemetry data: hidden Markov models and extensions. Ecology. 2012;93(11):2336–42. 23236905 10.1890/11-2241.1

[26] Michelot T, Langrock R, Patterson TA. movehmm: an r package for the statistical modelling of animal movement data using hidden Markov models. Methods Ecol Evol. 2016;7(11):1308–15.

[27] Zucchini W, MacDonald IL, Langrock R. Hidden Markov models for time series: an introduction using R. Chapman and Hall/CRC; 2016.

[28] Pohle J, Langrock R, van Beest FM, Schmidt NM. Selecting the number of states in hidden Markov models: pragmatic solutions illustrated using animal movement. J Agric Biol Environ Stat. 2017;22(3):270–93.

[29] Benhamou S. Of scales and stationarity in animal movements. Ecol Lett. 2014;17(3):261–72. 24350897 10.1111/ele.12225

[30] Getz WM. A hierarchical path-segmentation movement ecology framework. Ecol Process. 2022;11(1):1–15.

[31] Getz WN. An animal movement track segmentation framework for forecasting range adaptation under global change. Front Ecol Evol. 2023;11:1171169.

[32] Kays R, Hirsch B, Caillaud D, Mares R, Alavi S, Havmøller RW, Crofoot M. Multi-scale movement syndromes for comparative analyses of animal movement patterns. Mov Ecol. 2023;11(1):61. 37794525 10.1186/s40462-022-00365-y PMC10552421

[33] Gontier N. Hierarchies, networks, and causality: the applied evolutionary epistemological approach. J Gen Philos Sci. 2021;52(2):313–34.

[34] Getz WM. An information theory treatment of animal movement tracks. In L Giuggioli and P Maini, (eds.) The Mathematics of Movement: An Interdisciplinary Approach to Mutual Challenges in Animal Ecology and Cell Biology, page to appear (see arXiv:2403.16290). Springer, New York, 2024.

[35] Garde B, Wilson RP, Fell A, Cole N, Tatayah V, Holton MD, Rose KAR, Metcalfe RS, Robotka H, Wikelski M, et al. Ecological inference using data from accelerometers needs careful protocols. Meth Ecol Evol. 2022;13(4):813–25. 10.1111/2041-210X.13804 PMC9303593 35910299

[36] Van Walsum TA, Perna A, Bishop CM, Murn CP, Collins PM, Wilson RP, Halsey LG. Exploring the relationship between flapping behaviour and accelerometer signal during ascending flight, and a new approach to calibration. Ibis. 2020;162(1):13–26.

[37] Getz WM, Salter R, Vissat LL. Simulation applications to support teaching and research in epidemiological dynamics. BMC Med Educ. 2022;22(1):632. 35987608 10.1186/s12909-022-03674-3 PMC9391658

[38] Sethi V, Spiegel O, Salter R, Cain S, Toledo S, Getz W. An information theory framework for movement path segmentation and analysis. bioRxiv, 2024. 10.1101/2024.08.02.606194. URL https://www.biorxiv.org/content/early/2024/08/06/2024.08.02.606194.

[39] Kays R, Crofoot MC, Jetz W, Wikelski M. Terrestrial animal tracking as an eye on life and planet. Science. 2015;348(6240):aaa2478. 26068858 10.1126/science.aaa2478

[40] Beardsworth CE, Gobbens E, van Maarseveen F, Denissen B, Dekinga A, Nathan R, Toledo S, Bijleveld AI. Validating atlas: a regional-scale, high-throughput tracking system. Meth Mol Evol. 2022;13:1990–2004.

[41] Kazimierski LD, Catalano NE, Laneri K, Oliver AB, Calzolari G, Joseph J, Amico GC, Abramson G. Trajectory assessment of the vulnerable marsupial *Dromiciops gliroides* in the patagonian temperate forest. Mamm Biol. 2021;101:715.

[42] Owen-Smith N, Goodall V. Coping with savanna seasonality: comparative daily activity patterns of African ungulates as revealed by GPS telemetry. J Zool. 2014;293(3):181–91.

[43] Vissat LL, Cain S, Toledo S, Spiegel O, Getz WM. Categorizing the geometry of animal diel movement patterns with examples from high-resolution barn owl tracking. Mov Ecol. 2023;11(1):1–20. 36945057 10.1186/s40462-023-00367-4 PMC10029274

[44] Sören Häfker N, Tessmar-Raible K. Rhythms of behavior: are the times changin? Curr Opin Neurobiol. 2020;60:55–66. 31812940 10.1016/j.conb.2019.10.005

[45] Singh NJ, Börger L, Dettki H, Bunnefeld N, Ericsson G. From migration to nomadism: movement variability in a northern ungulate across its latitudinal range. Ecol Appl. 2012;22(7):2007–20. 23210316 10.1890/12-0245.1

[46] Schick RS, Loarie SR, Colchero F, Best BD, Boustany A, Conde DA, Halpin PN, Joppa LN, McClellan CM, Clark JS. Understanding movement data and movement processes: current and emerging directions. Ecol Lett. 2008;11(12):1338–50. 19046362 10.1111/j.1461-0248.2008.01249.x

[47] Codling EA, Hill NA. Sampling rate effects on measurements of correlated and biased random walks. J Theor Biol. 2005;233(4):573–88. 15748917 10.1016/j.jtbi.2004.11.008

[48] Shepard ELC, Wilson RP, Gareth Rees W, Grundy E, Lambertucci SA, Vosper SB. Energy landscapes shape animal movement ecology. Am Nat. 2013;182(3):298–312. 23933722 10.1086/671257

[49] Hernandez-Pliego J, Rodriguez C, Bustamante J. Gone with the wind: seasonal trends in foraging movement directions for a central-place forager. Curr Zool. 2014;60(5):604–15.

[50] Payne E, Spiegel O, Sinn DL, Leu ST, Gardner MG, Godfrey SS, Wohlfeil C, Sih A. Intrinsic traits, social context, and local environment shape home range size and fidelity of sleepy lizards. Ecol Monogr. 2022;92(3): e1519.

[51] Wilensky U, Rand W. An introduction to agent-based modeling: modeling natural, social, and engineered complex systems with NetLogo. Mit Press; 2015.

[52] Cain S, Solomon T, Leshem Y, Toledo S, Arnon E, Roulin A, Spiegel O. Movement predictability of individual barn owls facilitates estimation of home range size and survival. Mov Ecol. 2023;11(1):10. 36750910 10.1186/s40462-022-00366-x PMC9906850

[53] Corl A, Charter M, Rozman G, Toledo S, Turjeman S, Kamath PL, Getz WM, Nathan R, Bowie RCK. Movement ecology and sex are linked to barn owl microbial community composition. Mol Ecol. 2020;29(7):1358–71. 32115796 10.1111/mec.15398

[54] Getz WM. Biomass transformation webs provide a unified approach to consumer-resource modelling. Ecol Lett. 2011;14(2):113–24. 21199247 10.1111/j.1461-0248.2010.01566.x PMC3032891

[55] Thurfjell H, Ciuti S, Boyce MS. Applications of step-selection functions in ecology and conservation. Mov Ecol. 2014;2(1):4. 25520815 10.1186/2051-3933-2-4 PMC4267544

[56] Duchesne T, Fortin D, Rivest L-P. Equivalence between step selection functions and biased correlated random walks for statistical inference on animal movement. PLoS ONE. 2015;10(4): e0122947. 25898019 10.1371/journal.pone.0122947 PMC4405542

[57] Panzacchi M, Van Moorter B, Strand O, Saerens M, Kivimäki I, St CC, Clair IH, Boitani L. Predicting the continuum between corridors and barriers to animal movements using step selection functions and randomized shortest paths. J Anim Ecol. 2016;85(1):32–42. 25950737 10.1111/1365-2656.12386

[58] Avgar T, Potts JR, Lewis MA, Boyce MS. Integrated step selection analysis: bridging the gap between resource selection and animal movement. Methods Ecol Evol. 2016;7(5):619–30.

[59] Preisler HK, Ager AA, Wisdom MJ. Analyzing animal movement patterns using potential functions. Ecosphere. 2013;4(3):1–13.

[60] Gupte PR, Beardsworth CE, Spiegel O, Lourie E, Toledo S, Nathan R, Bijleveld AI. A guide to pre-processing high-throughput animal tracking data. J Anim Ecol. 2022;91(2):287–307. 34657296 10.1111/1365-2656.13610 PMC9299236

[61] Wolf T, Konrath R. Avian wing geometry and kinematics of a free-flying barn owl in flapping flight. Exp Fluids. 2015;56:1–18.

[62] Song J. Fly low: The ground effect of a barn owl (*Tyto alba*) in gliding flight. Proc Inst Mech Eng C J Mech Eng Sci. 2021;235(2):308–18.

[63] Grimm V, Berger U, Bastiansen F, Eliassen S, Ginot V, Giske J, Goss-Custard J, Grand T, Heinz SK, Huse G, et al. A standard protocol for describing individual-based and agent-based models. Ecol Model. 2006;198(1–2):115–26.

[64] Grimm V, Railsback SF, Vincenot CE, Berger U, Gallagher C, DeAngelis DL, Edmonds B, Ge J, Giske J, Groeneveld J, et al. The odd protocol for describing agent-based and other simulation models: a second update to improve clarity, replication, and structural realism. J Artif Soc Soc Simul. 2020;23(2):7.33204215

[65] Getz WM, Vissat LL, Salter R. Simulation and analysis of animal movement paths using numerus model builder. In: 2020 Spring Simulation Conference (SpringSim), pp. 1–12. IEEE, 2020.

[66] Doherty TS, Fist CN, Driscoll DA. Animal movement varies with resource availability, landscape configuration and body size: a conceptual model and empirical example. Landscape Ecol. 2019;34:603–14.

[67] Lubitz N, Bradley M, Sheaves M, Hammerschlag N, Daly R, Barnett A. The role of context in elucidating drivers of animal movement. Ecol Evol. 2022;12(7): e9128. 35898421 10.1002/ece3.9128 PMC9309038

[68] Brum-Bastos V, Łoś M, Long JA, Nelson T, Demšar U. Context-aware movement analysis in ecology: a systematic review. Int J Geogr Inf Sci. 2022;36(2):405–27.

[69] Fleming CH, Deznabi I, Alavi S, Crofoot MC, Hirsch BT, Patricia Medici E, Noonan MJ, Kays R, Fagan WF, Sheldon D, et al. Population-level inference for home-range areas. Methods Ecol Evol. 2022;13(5):1027–41.

[70] Silva I, Fleming CH, Noonan MJ, Fagan WF, Calabrese JM. movedesign: Shiny r app to evaluate sampling design for animal movement studies. Methods Ecol Evol. 2023;14(9):2216–25.

[71] Riotte-Lambert L, Matthiopoulos J. Environmental predictability as a cause and consequence of animal movement. Trends Ecol Evol. 2020;35(2):163–74. 31699411 10.1016/j.tree.2019.09.009

[72] Spiegel O, Harel R, Getz WM, Nathan R. Mixed strategies of griffon vultures (*Gyps fulvus*) response to food deprivation lead to a hump-shaped movement pattern. Mov Ecol. 2013;1:1–12. 25709819 10.1186/2051-3933-1-5 PMC4337378

[73] Calabrese JM, Fleming CH, Gurarie E. ctmm: an r package for analyzing animal relocation data as a continuous-time stochastic process. Methods Ecol Evol. 2016;7(9):1124–32.

[74] Auger-Méthé M, Newman K, Cole D, Empacher F, Gryba R, King AA, Leos-Barajas V, Flemming JM, Nielsen A, Petris G, et al. A guide to state-space modeling of ecological time series. Ecol Monogr. 2021;91(4): e01470.

[75] Paun I, Dirk Husmeier J, Hopcraft GC, Masolele MM, Torney CJ. Inferring spatially varying animal movement characteristics using a hierarchical continuous-time velocity model. Ecol Lett. 2022;25(12):2726–38. 36256526 10.1111/ele.14117 PMC9828272

[76] Vilk O, Orchan Y, Charter M, Ganot N, Toledo S, Nathan R, Assaf M. Ergodicity breaking in area-restricted search of avian predators. Phys Rev X. 2022;12(3):031005.

[77] Vilk O, Aghion E, Nathan R, Toledo S, Metzler R, Assaf M. Classification of anomalous diffusion in animal movement data using power spectral analysis. J Phys A Math Theor. 2022;55(33):334004.

[78] Fagan WF, Hoffman T, Dahiya D, Gurarie E, Cantrell RS, Cosner C. Improved foraging by switching between diffusion and advection: benefits from movement that depends on spatial context. Thyroid Res. 2020;13:127–36.

[79] Signer J, Fieberg J, Reineking B, SchlÃgel U, Smith B, Balkenhol N, Avgar T. Simulating animal space use from fitted integrated stepâ selection functions ( ISSF ). Meth Ecol Evol. 2023. 10.1111/2041-210X.14263.

[80] Wijeyakulasuriya DA, Eisenhauer EW, Shaby BA, Hanks EM. Machine learning for modeling animal movement. PLoS ONE. 2020;15(7):e0235750. 32716917 10.1371/journal.pone.0235750 PMC7384613

[81] Getz WM. An introspection on the art of modeling in population ecology. Bioscience. 1998;48(7):540–52.

[82] Lesne A. The discrete versus continuous controversy in physics. Math Struct Comput Sci. 2007;17(2):185–223.

[83] Bastille-Rousseau G, Douglas-Hamilton I, Blake S, Northrup JM, Wittemyer G. Applying network theory to animal movements to identify properties of landscape space use. Ecol Appl. 2018;28(3):854–64. 29420867 10.1002/eap.1697

[84] Yin S, Yanjie X, Mingshuai X, de Jong MCM, Huisman MRS, Contina A, Prins HHT, Huang ZYX, de Boer WF. Habitat loss exacerbates pathogen spread: An agent-based model of avian influenza infection in migratory waterfowl. PLoS Comput Biol. 2022;18(8):e1009577. 35981006 10.1371/journal.pcbi.1009577 PMC9426877

[85] Couzin ID, Krause J, Franks NR, Levin SA. Effective leadership and decision-making in animal groups on the move. Nature. 2005;433(7025):513. 15690039 10.1038/nature03236

[86] Getz WM, Salter R, Lyons AJ, Sippl-Swezey N. Panmictic and clonal evolution on a single patchy resource produces polymorphic foraging guilds. PLoS ONE. 2015;10(8):e0133732. 26274613 10.1371/journal.pone.0133732 PMC4537111

[87] Getz WM, Salter R, Seidel DP, Van Hooft P. Sympatric speciation in structureless environments. BMC Evol Biol. 2016;16:1–12. 26922946 10.1186/s12862-016-0617-0 PMC4770699

[88] Salecker J, Sciaini M, Meyer KM, Wiegand K. The NLRX r package: a next-generation framework for reproducible Netlogo model analyses. Methods Ecol Evol. 2019;10(11):1854–63.

[89] Xie J, Girshick R, Farhadi A. Unsupervised deep embedding for clustering analysis. In International conference on machine learning, pp. 478–487. PMLR, 2016.

[90] Fawaz HI, Forestier G, Weber J, Idoumghar L, Muller P-A. Deep learning for time series classification: a review. Data Min Knowl Disc. 2019;33(4):917–63.

[91] Karim MR, Beyan O, Zappa A, Costa IG, Rebholz-Schuhmann D, Cochez M, Decker S. Deep learning-based clustering approaches for bioinformatics. Brief Bioinform. 2021;22(1):393–415. 32008043 10.1093/bib/bbz170 PMC7820885

[92] Lafabregue B, Weber J, Gançarski P, Forestier G. End-to-end deep representation learning for time series clustering: a comparative study. Data Min Knowl Disc. 2022;36(1):29–81.

[93] Ren Y, Pu J, Yang Z, Xu J, Li G, Pu X, Yu PS, He L. Deep clustering: a comprehensive survey. arXiv preprint arXiv:2210.04142, 2022. 10.1109/TNNLS.2024.3403155 38963736

[94] Rama ÓJ , Moreno-Pino F, Ramírez D, Olmos PM. Interpretable spectral variational autoencoder (ISVAE) for time series clustering. arXiv e-prints, pp. arXiv–2310, 2023.

[95] Wu H, Liu Q, Liu X. A review on deep learning approaches to image classification and object segmentation. Comput Mater Contin. 2019;60(2):575–97.

[96] He X, Montillet J-P, Fernandes R, Bos M, Kegen Y, Hua X, Jiang W. Review of current GPS methodologies for producing accurate time series and their error sources. J Geodyn. 2017;106:12–29.

[97] Arnold DN. Stability, consistency, and convergence of numerical discretizations. In: Encyclopedia of applied and computational mathematics. Berlin: Springer; 2015. p. 1358–64.

[98] Klarevas-Irby JA, Farine DR. Diel patterns of movement reveal temporal strategies during dispersal. Anim Behav. 2024;207:119–29.

[99] Owen-Smith N, Hopcraft G, Morrison T, Chamaillé-Jammes S, Hetem R, Bennitt E, Van Langevelde F. Movement ecology of large herbivores in African savannas: current knowledge and gaps. Mammal Rev. 2020;50(3):252–66.

[100] Abrahms B, Seidel DP, Dougherty E, Hazen EL, Bograd SJ, Wilson AM, Weldon McNutt J, Costa DP, Blake S, Brashares JS, et al. Suite of simple metrics reveals common movement syndromes across vertebrate taxa. Mov Ecol. 2017;5(1):1–11. 28580149 10.1186/s40462-017-0104-2 PMC5452391

[101] Seidel DP, Linklater WL, Kilian W, du Preez P, Getz WM. Mesoscale movement and recursion behaviors of Namibian black rhinos. Mov Ecol. 2019;7(1):1–14. 31728193 10.1186/s40462-019-0176-2 PMC6842456

[102] Owen-Smith N. Daily movement responses by African savanna ungulates as an indicator of seasonal and annual food stress. Wildl Res. 2013;40(3):232–40.

[103] Owen-Smith N, Martin J. Identifying space use at foraging arena scale within the home ranges of large herbivores. PLoS ONE. 2015;10(6):e0128821. 26066834 10.1371/journal.pone.0128821 PMC4466150

[104] Muñoz-Gil G, Garcia-March MA, Manzo C, Martín-Guerrero JD, Lewenstein M. Single trajectory characterization via machine learning. New J Phys. 2020;22(1):013010.

[105] Spiegel O, Leu ST, Bull CM, Sih A. What’s your move? Movement as a link between personality and spatial dynamics in animal populations. Ecol Lett. 2017;20(1):3–18. 28000433 10.1111/ele.12708

[106] Torres JF, Hadjout D, Sebaa A, Martínez-Álvarez F, Troncoso A. Deep learning for time series forecasting: a survey. Big Data. 2021;9(1):3–21. 33275484 10.1089/big.2020.0159

[107] Lim B, Zohren S. Time-series forecasting with deep learning: a survey. Phil Trans R Soc A. 2021;379(2194):20200209. 33583273 10.1098/rsta.2020.0209

