# Supplementary material for: The statistical building blocks of animal movement simulations
Source: Mov Ecol. 2024 Sep 30;12:67. doi: 10.1186/s40462-024-00507-4 (PMC11440923; doi:10.1186/s40462-024-00507-4)
Supplement: Supplementary file 1 [file 40462_2024_507_MOESM1_ESM.pdf]

Appendices

These appendices contain references to equations and tables appearing in the main text of *Getz et al., The Statistical Building Blocks of Animal Movement Simulations* and the bibliographic citations herein are included in the *References* section of that text.

A Hierarchical Segmentation and Empirical Data

A.1 Issues of scale

We have various temporal scales in our model as they relate to the following structures and processes from likely fastest to slowest:

1. FuME scale (fundamental movement element). The scale of the actual "hidden" movement elements underlying the StaMEs (previously called metaFuMEs). This is the average time it takes to perform a typical FuME, such as one repeatable sequence when striding, wing flapping, wiggling or undulating one's body.

2. Landscape traversal scale. This converts the landscape pixel scale to the typical time it takes for individual to move across one pixel. This scale is typically finer than the relocation data scale (see next), though if the pixels are large (e.g., 10 meters or more) and the relocation data scale fine then this scale may be coarser than the relocation data scale.

3. Relocation data scale. This is set by the frequency of the relocation data. Fine scale implies that data points are less than 10 secs apart. Course scale implies points more than 1 minute apart. Intermediate scale is between these two.

4. StaMe scale (statistical movement elements, formerly metaFuMEs). This is the scale of our StaME elements—i.e., it relates to the number of relocation data points (including missing points) used to compute our StaMEs, which my vary from typically 10 to 30 points, depending on the fineness of the relocation data scale.

5. CAM scale (Canonical activity mode) (formerly short or homogeneous CAMs). This scale relates to the typical length of a homogeneous sequence of some underlying metaFuMEs. This scale will vary somewhat, depending on the type of behavior. Directed walking, for example, may last several hours, though it will likely be interspersed with pauses when the individual rests or scans their surrounding for information relating to navigation or safety. Grazing, which is a mix of several different FuMEs that each last a short time (e.g., stop-start motions involving forward, sideways and backward movements coupled with various head motions) may also last on the order of large fractions of an hour or multiple hours.

6. Activity type scale (formerly long CAMs). When relocation data is relatively fine (consecutive points are seconds apart) then "activity types" may be identified in terms of a characteristic mix of CAMs. Thus a migration movement CAM that lasts for a substantial part of a diel may be made up of a characteristic mix of directed movement, grazing, and resting CAMs. On the other hand, if the relocation data scale is relatively course (i.e., relocation points are minutes apart) then StaMEs reflect average movement behavior during "migration movement" may already have statistics that reflect a mix of directed movement, grazing, and resting behaviors.

A.2 Movement path data and segmentation

Here is a refinement of the hierarchical definitions provided in [30] to help us understand how to parse out the information contained in the planar points relocation time series or walk (recall Eq 12)

$$W = \left\{ \left(t; x_t^{\mathrm{id}}, y_t^{\mathrm{id}}\right) \middle| t = 0, ..., n^{\mathrm{time}} \right\}$$

From this time series, the following step length S, or equivalently velocity V and turning-angle $\Delta\Theta$ time series, can be derived using the relocation point frequency F

$$V = \{(t, v_t|\ t = 1, ..., n^{\text{time}}\} \tag{A.1}$$

using the derived values

$$v_t = F\sqrt{\left(x_t^{\text{id}} - x_{t-1}^{\text{id}}\right)^2 + \left(y_t^{\text{id}} - y_{t-1}^{\text{id}}\right)^2} \tag{A.2}$$

and

$$\Delta\Theta = \{(t, \Delta\theta_t|\ t = 2, ..., n^{\text{time}}\} \tag{A.3}$$

using the derived values

$$|\Delta\theta_t| = \min\{|\theta_t - \theta_{t-1}|, 2\pi - |\theta_t - \theta_{t-1}|\}, \qquad \text{where}\ \ \theta_t = \arctan\left(\frac{y_t^{\text{id}} - y_{t-1}^{\text{id}}}{x_t^{\text{id}} - x_{t-1}^{\text{id}}}\right) \tag{A.4}$$

and the sign of $|\Delta\theta_t|$ is easily determined by considering which quadrants θ_{t-1} and θ_t are in and whether moving clockwise or anticlockwise subscribes the smaller of the two angles between them: anticlockwise implies positive and clockwise negative. As in [18], we may also study movement in terms of the persistent (v^{per}) and tangential (v^{tan}) velocities rather than speed (v or stepsize) and turning angle ($\delta\theta$), using the transformations

$$v_t^{\text{per}} = v_t \cos(\theta_t) \quad \text{and} \quad v_t^{\text{tan}} = v_t \sin(\theta_t) \tag{A.5}$$

FuME A FuME is a fundamental movement element that for some types may be hard to observe its start and end points, or even define with any precision

Step A step is one point t in the time series W with which we associate a step length $s(t)$ and turning angle $\Delta\theta(t)$

StaME (previously called metaFuMEs) These are statistically entities computed from consecutive segments of fixed length of a walk W (Eq 12) (i.e., each segment contains a specified number of consecutive relocation points—typically 10-30 to obtain reasonable statistics). These segments can be classified into a few different StaME categories or types based on their statistics—typically the mean and variances of the persistent and turning velocities $v(p)$ and h_t obtained for each segment.

CAM A canonical activity mode, formerly referred as a "short" or " homogeneous" CAM that is a consecutive sequence of two or more StaMEs of the same type.

Activity type Formerly referred to as a long CAM, and is a characteristic mix of CAMs defining a complex movement activity such as grazing (a mix of stop-start movements)

DAR A diel activity routine with start and stop points in its 24 hour cycle selected to correspond, for example, to its major resting period.

LiMP A lifetime movement phase is a sequence of DARs that occurs during the individuals life history. It may be a migration event that occurs over several days or weeks, or it may be a seasonal movement pattern (e.g., a nomadic phase, or a home ranging phase) that repeats annually.

LiT The lifetime track of an individual from the moment it starts moving after birth to the moment it ceases to move at death.

A.3 Step-selection procedures

ANIMOVER_1 includes two movement modes, each involving its own step selection procedure. One specifies the movement of individuals within patches (wp) and a second between patches (bp). Each of these two modes involves a kernel with mode-specific parameters $K_\alpha = K\left(r_\alpha^{\min}, r_\alpha^{\max}, \psi_\alpha\right)$, $\alpha =$ wp, bp (Eq 4), as well as mode specific time-in-mode parameter $\hat{t}_t^{s_\alpha}$ (i.e. the value of t^{s_α} at time t) and neighborhood value parameter $\hat{c}_\alpha^{\text{nbh}}$. The procedures also involve updating individual and cellular array states using Eqs 6 or 7, depending on which of the `RAMs` (see Section 4.2) have been selected.

Within patches step-selection procedure, $\mathcal{R}_{\rm wp}(\hat{t}^{s_{\rm wp}}, \hat{c}^{\rm nbh}_{\rm wp})$

$\mathcal{R}_{\rm wp}.1$ *SET* $\alpha_t = \alpha_{\rm wp,next}$, $\left(x^{\rm id}_t, y^{\rm id}_t, \theta_t, t^{\rm s}_t\right) = (x_{\rm next}, y_{\rm next}, \theta_{\rm next}, t^{\rm s}_{\rm next})$, and $(c_{ab,t}, h) = (c_{ab,{\rm next}}, h_{\rm next})$ (Fig 3).

$\mathcal{R}_{\rm wp}.2$ *COMPUTE* the values of the admissible set of cells $\mathcal{C}^{\rm wp}_t$ (Eq 9) and *COMPUTE* $c^{\max}_{ab} = \max\{c_{ab} \mid {\rm cell}(a,b) \in \mathcal{C}^{\rm wp}_t\}$. Note, for the case `topology`=`plane`, there may be no admissible cells.

$\mathcal{R}_{\rm wp}.3$ *COMPUTE*

$$\text{Sum}_9\,(c^{\max}_{ab}) = c^{\max}_{ab} + \text{ sum of values in 8-cell Moore neighborhood of } c^{\max}_{ab} \text{ cell}$$

$\mathcal{R}_{\rm wp}.4$ *IF* $\text{Sum}_9\,(c^{\max}_{ab}) \geq \hat{c}^{\rm nbh}_{\rm wp}$ *THEN*

(a) *COMPUTE* the set of probabilities $\mathcal{P}^{\rm wp}_t$ (Eq 10) associated with selecting one of the admissible cells

(b) *SELECT* the next cell, cell(a,b) using the probabilities in $\mathcal{P}^{\rm wp}_t$ in a multinomial drawing (i.e., the cell most likely to be selected is the one with the largest p_{ab} and so on)

ELSE

(c) *MODIFY* $\tilde{K}^{\rm wp}$ by replacing $r^{\max}_{\rm wp}$ with $r^{\max}_{\rm bp}$, $r^{\min}_{\rm wp}$ with 0 (typically we already have $r^{\min}_{\rm wp} = 0$), and $\psi_{\rm wp}$ with π and repeat steps $\mathcal{R}_{wp}.2$ & 3.

(d) *APPLY* rule $\mathcal{R}_{\rm wp}$ 4a & b to the selected cell.

(e) *IF* the updated $\text{Sum}_9\,(c_{ab}) \geq \hat{c}^{\rm nbh}_{\rm wp}$ *THEN* move to this cell

(f) *ELSE* move at random to one of the cells on the rim of a circle at radius $r^{\max}_{\rm bp}$ from the current location (i.e., all points whose distance from the current location is within the band $\left[r^{\max}_{\rm bp} - \sqrt{\Delta x^2 + \Delta y^2}, r^{\max}_{\rm bp}\right]$). We note that this step will also prevent an individual from getting stuck at a boundary when `topology` = `plane`.

$\mathcal{R}_{\rm wp}.5$ Now that we have identified the next cell(a,b) to be occupied:

(a) *SET* $(x_{\rm next}, y_{\rm next}) = (x^{\rm cell}_a, y^{\rm cell}_b)$.

(b) *COMPUTE* the next angle of heading $\theta_{\rm next}$ (Eq 3) and distance moved $\rho_{ab}(x_{\rm next}, y_{\rm next})$ (Eq 2).

(c) *UPDATE* $c_{ab\,{\rm next}}$ and $h_{\rm next}$ using $\texttt{RAM}^{\rm update}_0$ (Eq 6) or $\texttt{RAM}^{\rm update}_1$ (Eq 7) or a user specified $\texttt{RAM}^{\rm update}_2$

$\mathcal{R}_{\rm wp}.6$ *UPDATE* the StaME index $\alpha_{\rm next}$ using probability $p_{\rm wp}(t^{\rm s})$ (Eq 11) as follows

(where $\text{Sum}_9\,(c_{ab})$ is sum of values in the 8-cell Moore neighborhood of Cell(a,b) plus c_{ab} itself)

IF $Z \sim \texttt{BINOMIAL}[p_{\rm wp}(t^{\rm s})] = 1$ *AND* $\text{Sum}_9\,(c_{ab}) \geq \hat{c}^{\rm nbh}_{\rm wp}$ (if updated under $\mathcal{R}$.4c use latest value) *THEN* $\alpha_{\rm next} = {\rm wp}$ *ELSE* $\alpha_{\rm next} = {\rm bp}$

Between patches step-selection procedure, $\mathcal{R}_{\rm bp}(\hat{t}^{s_{\rm bp}}, \hat{c}^{\rm nbh}_{\rm bp})$

$\mathcal{R}_{\rm bp}.1$ *SET* $\alpha_t = \alpha_{\rm bp,next}$, $\left(x^{\rm id}_t, y^{\rm id}_t, \theta_t, t^{\rm s}_t\right) = (x_{\rm next}, y_{\rm next}, \theta_{\rm next}, t^{\rm s}_{\rm next})$, and $(c_{ab,t}, h) = (c_{ab,{\rm next}}, h_{\rm next})$ (Fig 3).

$\mathcal{R}_{\rm bp}.2$ *COMPUTE* the values of the admissible set of cells $\mathcal{C}^{\rm vis}_t$ (i.e., using $\tilde{K}^{\rm vis}_t(0, r^{\max}_{\rm wp}, \pi/2)$ rather than $\tilde{K}^{\rm bp}_t$ in Eq 9) and *COMPUTE* $c^{\max}_{ab} = \max\{c_{ab} \mid {\rm cell}(a,b) \in \mathcal{C}^{\rm vis}_t\}$. Note, for the case `topology`=`plane`, there may be no admissible cells when the individual is on the boundary near a corner. In this case, when no visible cells are available, then use $K_{\rm vis}(0, r^{\max}_{\rm bp}, \pi)$, i.e., the individual now looks behind itself as well.

$\mathcal{R}_{\rm bp}.3$ *COMPUTE*

$$\text{Sum}_9\,(c^{\max}_{ab}) = c^{\max}_{ab} + \text{ sum of values in 8-cell Moore neighborhood of } c^{\max}_{ab} \text{ cell}$$

$\mathcal{R}_{\rm bp}.4$ *IF* $\text{Sum}_9\,(c^{\max}_{ab}) \geq \hat{c}^{\rm nbh}_{\rm wp}$ *THEN*

(a) *COMPUTE* the set of probabilities $\mathcal{P}^{\rm wp}_t$ (Eq 10) associated with selecting one of the admissible cells

(b) *SELECT* the next cell, $\text{cell}(a,b)$ using the probabilities in in a multinomial drawing

ELSE move to any cell at random in the set $\mathcal{C}^{\rm bp}_t$ (i.e., overlapping with $\tilde{K}^{\rm wp}_t$).

$\mathcal{R}_{\rm bp}.5$ Now that we have identified the next $\text{cell}(a,b)$ to be occupied:

(a) *SET* $(x_{\rm next}, y_{\rm next}) = (x^{\rm cell}_a, y^{\rm cell}_b)$.

(b) *COMPUTE* the next angle of heading $\theta_{\rm next}$ (Eq 3) and distance moved $\rho_{ab}(x_{\rm next}, y_{\rm next})$ (Eq 2).

(c) *UPDATE* $c_{ab\,{\rm next}}$ and $h_{\rm next}$, but for this movement mode setting $\kappa^{\rm add} = 0$ in the same $\texttt{RAM}^{\rm update}$ used in $\mathcal{R}_{\rm wp}.6$

$\mathcal{R}_{\rm bp}.6$ *UPDATE* the StaME index $\alpha_{\rm next}$ using probability $p_{\rm bp}(t^{\rm s})$ (Eq 11) as follows

(where $\text{Sum}_9\,(c_{ab})$ is sum of values in the 8-cell Moore neighborhood of $\text{Cell}(a,b)$ plus c_{ab} itself)

IF $Z \sim \texttt{BINOMIAL}[p_{\rm bp}(t^{\rm s})] = 1$ *AND* $\text{Sum}_9\,(c_{ab}) \leq \hat{c}^{\rm nbh}_{\rm bp}$ *THEN* $\alpha_{\rm next} = \text{bp}$ *ELSE* $\alpha_{\rm next} = \text{wp}$

A.4 StaMEs from empirical data

Various methods can be applied to extract StaMEs from empirical data. We have outlined one method in Section 3 of our text. Here we present more comprehensive details on the method we use. In order to separate different movement modes from each other, we perform a cluster analysis. Clustering is an unsupervised machine learning procedure which finds an optimum partition of a set of points with two or more variables into subsets (or clusters) of similar points.

Here we use hierarchical clustering approach, which results in a tree structure called dendrogram having a single cluster at the root, with leaf nodes representing data points in the set. Recall from Eq 13 that the variables $\mathcal{S}_\mu$ had been constructed using the relocation time series W in Eq 12. which we repeat here for convenience:

$$\mathcal{S}_\mu = \left\{ \text{Seg}_z\big(V_z, \text{SD}^V_z, |\Delta\Theta|_z, \text{SD}^{|\Delta\Theta|}_z, \Delta^\rho\big) \big| z = \lfloor \frac{t}{\mu} \rfloor + 1,\ t = 0, \cdots, n^{\rm time} - 1 \right\} \text{ such that}$$

$$V_z = \frac{\sum_{\tau=t}^{t+\mu-1} v_\tau}{v_{\max}\mu} \text{ with st. dev. } \text{SD}^V_z,\ |\Delta\Theta|_z = \frac{\sum_{\tau=t}^{t+\mu-1} |\Delta\theta_\tau|}{2\pi\mu} \text{ with st. dev. } \text{SD}^{|\Delta\Theta|}_z$$

$$\text{and } \Delta^\rho = \frac{\sqrt{(x^{\rm id}_{t+\mu} - x^{\rm id}_t)^2 + (y^{\rm id}_{t+\mu} - y^{\rm id}_t)^2}}{\mu V_z}.$$

To begin with, it is important to discard any points containing missing or non-numeric values (since the variables to be used in clustering algorithm are all numeric). Further, each of these variables ranges in $[0,1]$, and hence no further normalisation has been carried out. Cluster analysis is performed on the segment level data (i.e., with $n^{\rm seg} = \lfloor \frac{t}{\mu} \rfloor + 1$ points) using the variables in $\mathcal{S}_\mu$. Specifically, we want to find an optimal partition

$$\mathcal{P}(\mathcal{S}_\mu) = \{C_1, C_2, ..., C_k\},$$

where $\cup_{i=1}^k C_i = \mathcal{S}_\mu$, and $C_i \cap C_j = \emptyset$ (hard clustering).

Hierarchical agglomerative algorithms implement bottom-up clustering methodology, which starts with each point of $\mathcal{S}$ in its own singleton cluster, followed by successive fusions of two clusters at a time, depending on similarity between them, leading to a specified number of clusters (or, alternatively, leading to one cluster followed by cutting the dendrogram at the desired number of clusters). These are deterministic, yet greedy,

in the sense that the clusters are merged based entirely on the similarity measure, thereby yielding a local solution. Most of the similarity schemes are specified not in terms of an objective function to be optimized, but procedurally. We use Ward's minimum sum of square scheme, which performs a fusion of clusters while minimizing the intra-cluster variance. Our distance metric to quantify dissimilarity is the Euclidean measure.

To perform clustering, we make use of `hclust` function from `fastcluster` `R` package. It replaces the `stats`:: `hclust` function, which offers the most common implementation of Ward's hierarchical clustering in `R`. The conventional algorithm from `stats` package takes as input the set of points $\{\mathbf{x}_z\}|_{z=1}^{n^{\text{seg}}} = \{V_z,\ \text{SD}_z^V,\ |\Delta\Theta|_z,\ \text{SD}_z^{|\Delta\Theta|}, \Delta_z^{\rho}\}|_{z=1}^{n^{\text{seg}}} \in \mathbb{R}^{n^{\text{seg}}\times 5}$ (represented by $\mathcal{S}_\mu$) and a pair-wise dissimilarity matrix $d(C_i, C_j)|_{i,j=1}^{n^{\text{seg}}}$ (to be computed in advance). Starting with n^{seg} clusters, it fuses multiple pairs of them in each of $n^{\text{seg}} - 1$ steps while satisfying Ward's criterion and updates the dissimilarity matrix with the distance of each of the clusters still available to be merged with the newly created cluster. Accordingly, a series of partitions are produced, with the first one containing singleton clusters and the last one containing all n^{seg} points in one cluster. At each step, a set of clusters available to be merged (active clusters) is maintained, and each merger is tracked leading to a dendrogram. Concisely, the algorithm can be represented as shown in Algorithm 1. The dendrogram thus produced can also be understood as a weighted graph, with leaf

Algorithm 1: Naive hierarchical agglomerative clustering algorithm

```
Initialize active set with each instance in singleton cluster
repeat
    Select the pair of clusters with the most suitable distance
    Remove each from the active set
    Add their union to the tree
until Active set has only one item
```

nodes representing data points, and each internal node representing the cluster of its descendent leaves. The dissimilarity between clusters is represented by edge weights. Built-in `R` function `cutree` can then be used to cut the dendrogram at the required number of clusters, and `colMeans` can be used to compute a cluster's center.

This algorithm has a time complexity of $\mathcal{O}(n^{\text{seg}^3}$ and requires $\Omega(n^{\text{seg}^2})$ memory (on account of distance matrix computation and storage), making it unsuitable for our segment-level data set with $\sim\ 10^5$ points. Further, it is also difficult to distribute over multiple threads because the complete dissimilarity matrix along with active clusters and current state of dendrogram is required by all the processes. To get around these difficulties, we make two modifications to this workflow. First, we make use of `parDist` function of `parallelDist` package, which permits parallel computations of pair-wise dissimilarities. It offers the same interface as that of `stats::dist` `R` function. Second, we use an improved algorithm for hierarchical agglomerative clustering from `fastcluster` package, as mentioned above. The algorithm performs hierarchical clustering with Ward's scheme faster by accomplishing the search for the best cluster to merge with any cluster in the most efficient way [108]. For the clustering procedure, we choose to not perform dimensional reduction and use all 5 variables in $\mathcal{S}_\mu$. This ensures enhanced interpretation of results.

As noted above, the number of clusters k needs to be specified in order to cut the dendrogram. As is evident from Figures 7 and 8, we selected $k = 8$ to be used in our analyses. We initially choose to err on the side of having more than fewer clusters than may turn out to be optimal because we wanted to try to isolate clusters at the extremes of the cluster groups that were as homogeneous as possible in terms of not mixing many different movement modes in one movement segment. It may also be useful in some particular cases to use a gap statistic to justify one's choice of k. For example, in the two-movement mode simulation with 10-step segmentation we used a Gap statistic that compares the total intra-cluster variance for different values of k with the corresponding no clustering case. In this case, we see in Fig A.1 that the rate of increase of our Gap statistic somewhat flattens at $k = 8$. Accordingly, this would be the appropriate choice for our 10-step segmentation two-mode movement simulation. A relatively large value large value of k allows us to consider how close the centroids of different clusters would be and what it may mean to combine one

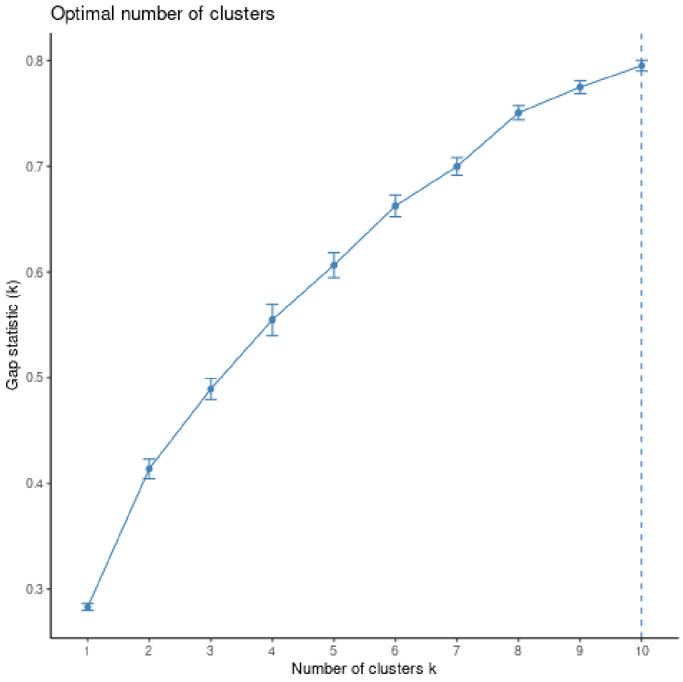

Figure A.1: Gap statistic for the two-movement mode simulation data in section 5.2.

or more clusters. Also, every data set may have a different preferential value for k when considering a gap statistic. Further, to facilitate comparisons across our analyses of different data sets, we kept to our selection of $k = 8$. As mentioned in the discussion section, hierarchical clustering is but one of many methods that may be used to cluster the data and other machine learning and even deep learning methods [109, 110, 111], can be explored, particularly when segment shapes vary widely.

Our computations have been performed on a node of Berkeley's HPC Cluster Savio. The system is Lenovo NeXtScale nx360m5 equipped with two Intel Xeon 12-core Haswell processors (core frequency 2.3 Ghz) and 128 GB of 2133 Mhz DDR4 memory. The `R` function `parDist` distributes distance matrix calculation over all of 24 available processor cores, and returns a `"dist"` class object - the lower triangle of the distance matrix (since it is symmetric). `hclust` returns a list with several components describing the dendrogram. `cutree` method, which is used to cut the tree, returns a vector of integers indicating which cluster each point has been assigned to.

The segment level data in $\mathcal{S}$ has 5 variables. Accordingly, principal components analysis is required to visualize the clustering results. This is a coordinate transformation procedure to a new set of variables such that maximum variance is preserved (which amounts to minimum loss of information). The two principal components accounting for maximum variance are used for 2-d cluster plots.

Our speed and turning-angle measurements are scaled with maximum speed and π, respectively, ensuring that the segment level data has all variables ranging in $[0, 1]$. This makes it reasonable to calculate principal components without further normalization. For better interpretation, we report the variable $\overline{V} \pm \overline{\mathrm{SD}^V}$ in Table 2 in units of m/s after rescaling with the maximum speed. PCA has been performed using built-in R method `prcomp`. To ensure no further normalization, the binary arguments `center` and `scale` in `prcomp` (which govern whether the variables are zero centered, and whether the variables are scaled to have unit variance, respectively) are set to `FALSE`. The function returns a list with various components which can be used to read eigenvectors and eigenvalues (and hence standard deviations of principal components) of the covariance matrix.

A.5 Plots of 10-step simulation data segments

Each plot in this subsection, particularly across different clusters, has had its axes for each panel automatically set by the plotting routine. Thus the size of segments is not comparable, only their relative shapes is informative.

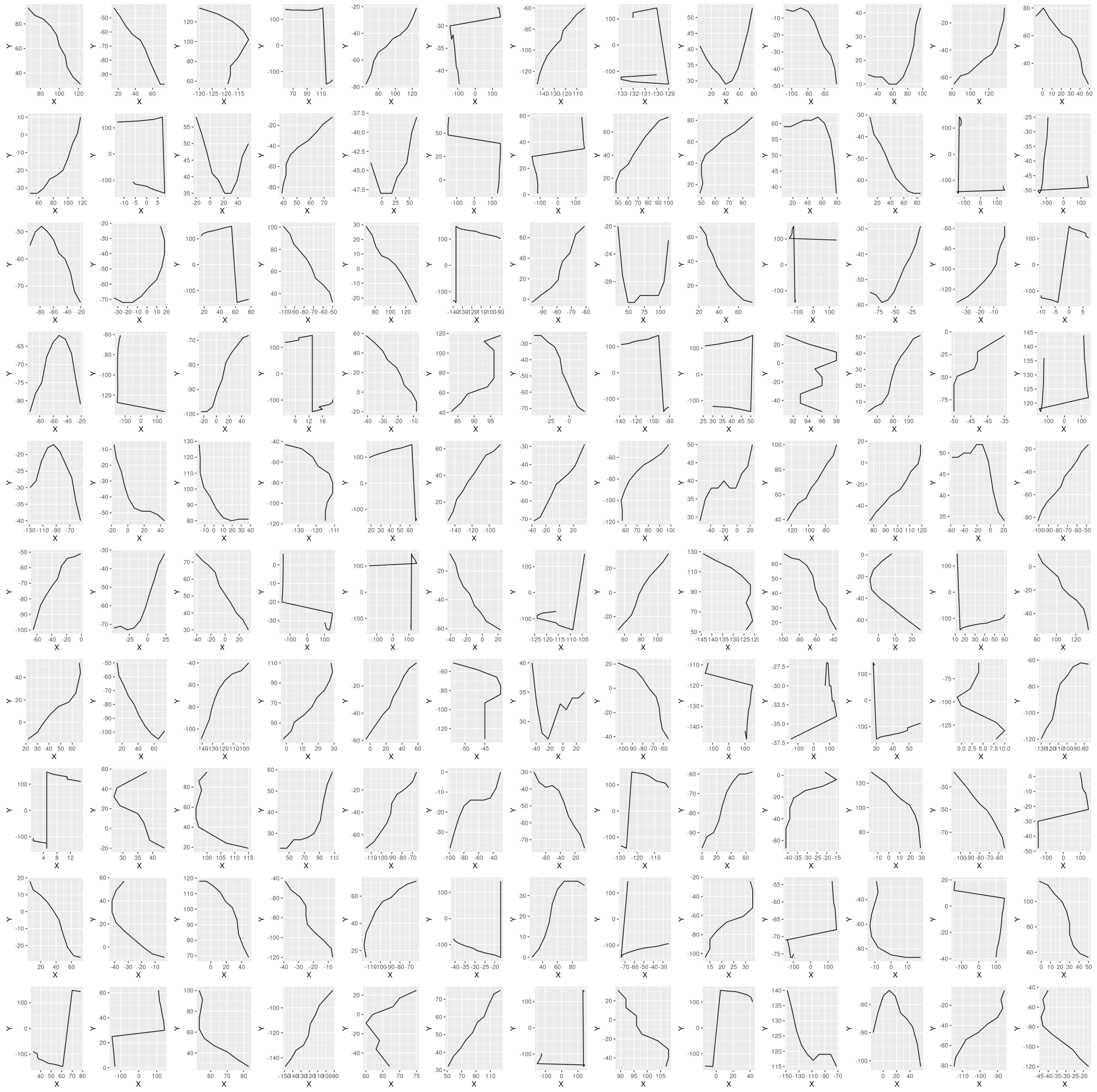

Figure A.2: 130 random 10-step segments from cluster C=1 (Table 1)

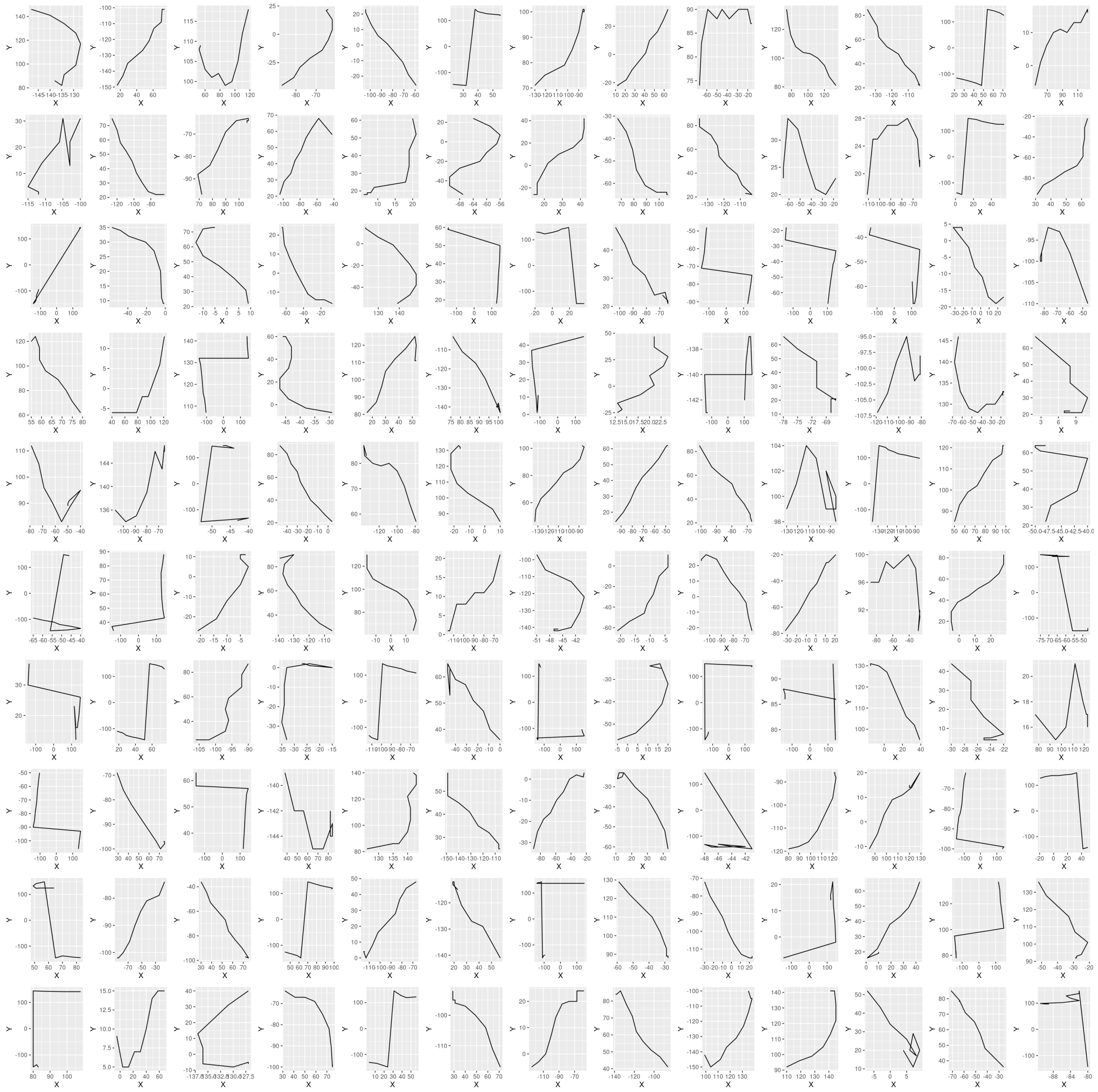

Figure A.3: 130 random 10-step segments from cluster C=2 (Table 1)

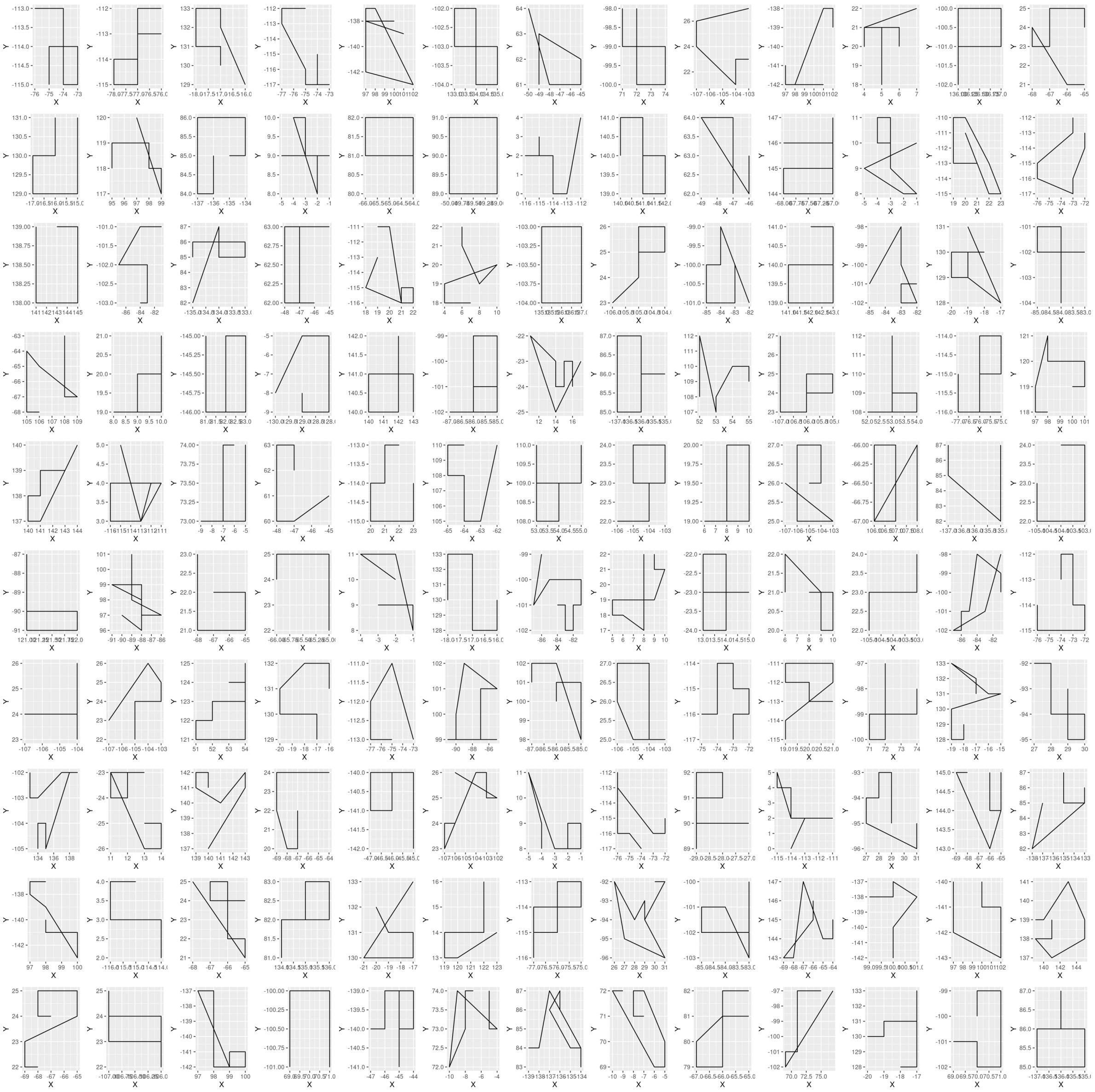

Figure A.4: 130 random 10-step segments from cluster C=3 (Table 1)

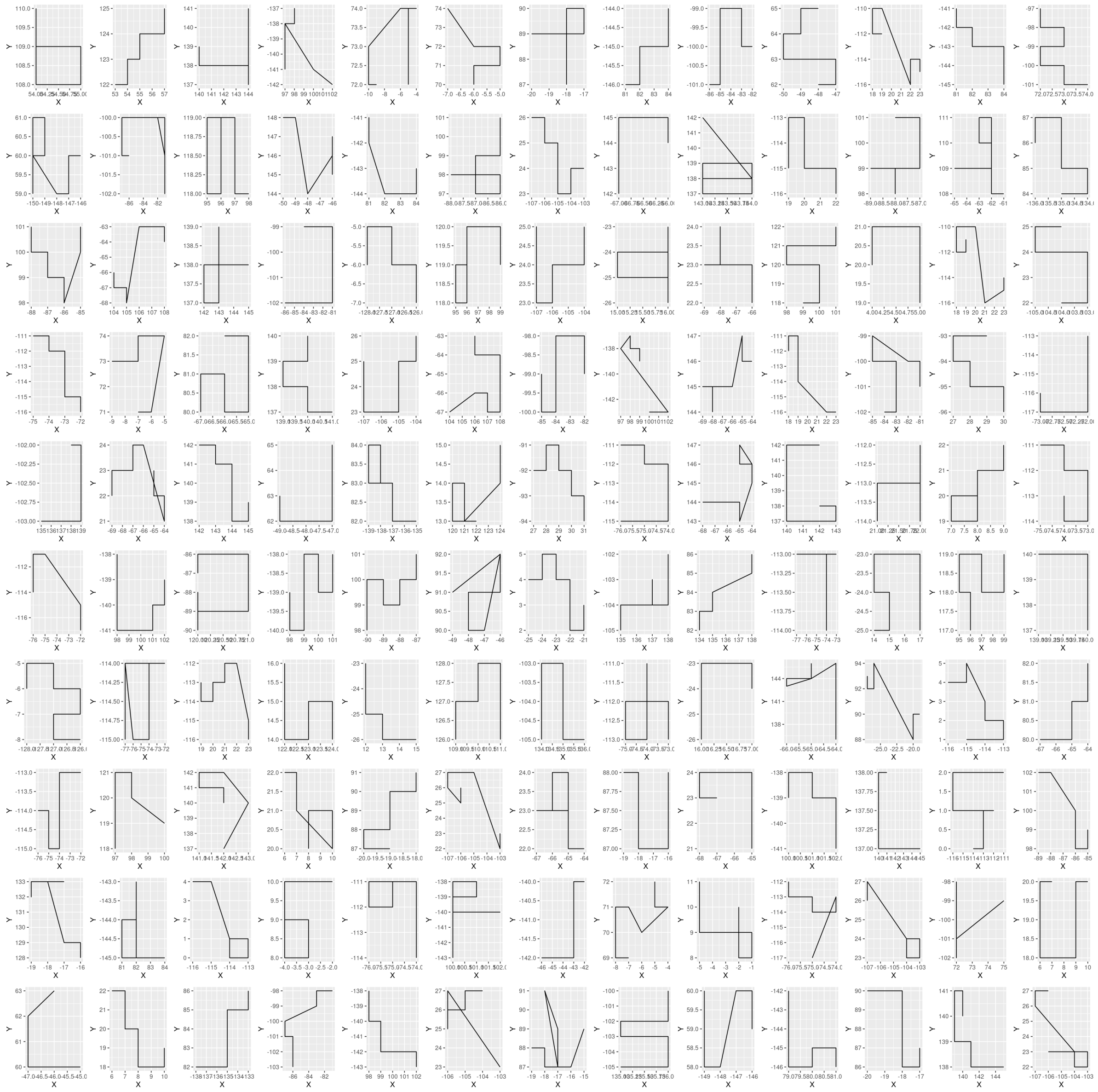

Figure A.5: 130 random 10-step segments from cluster C=4 (Table 1)

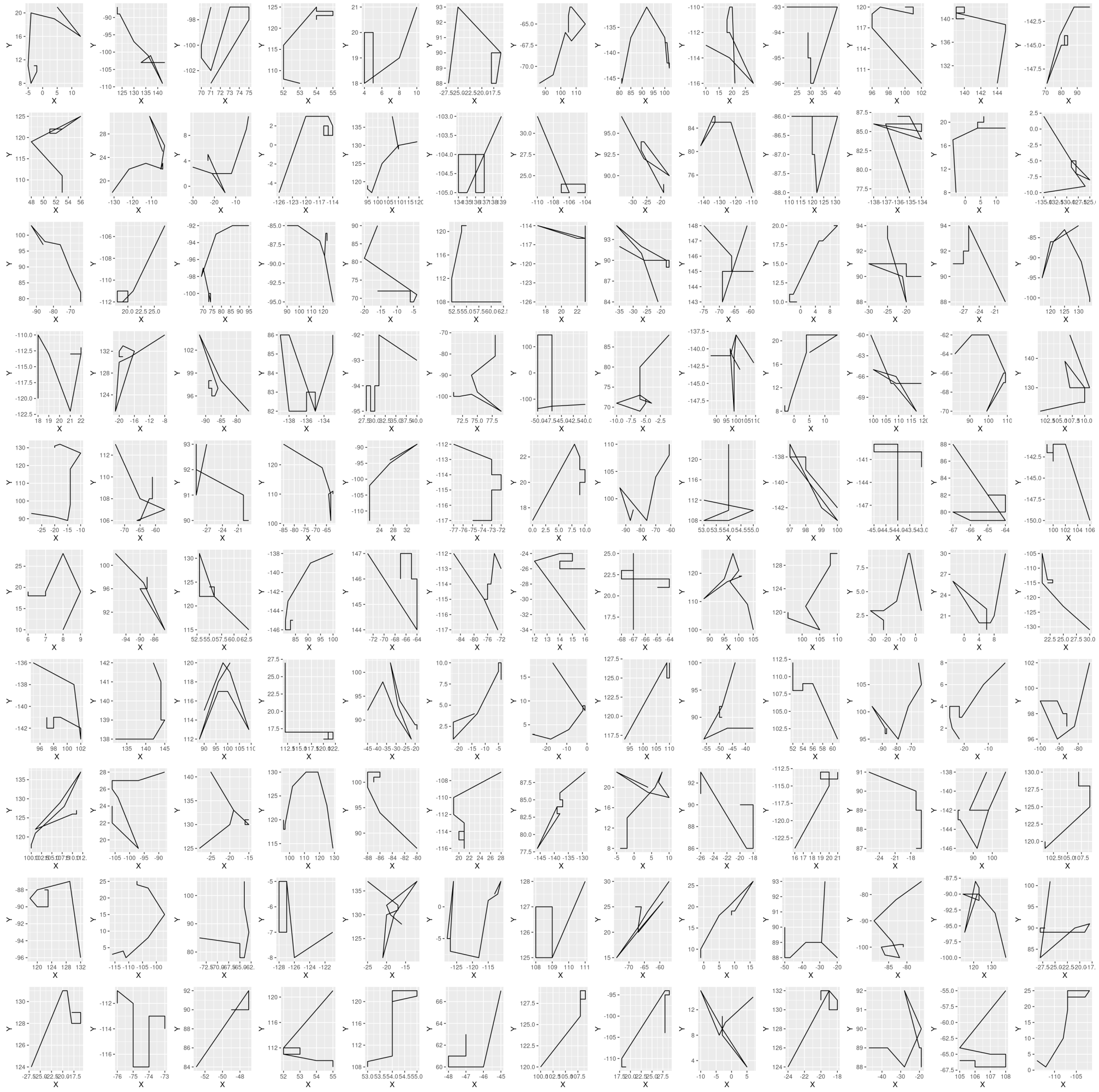

Figure A.6: 130 random 10-step segments from cluster C=5 (Table 1)

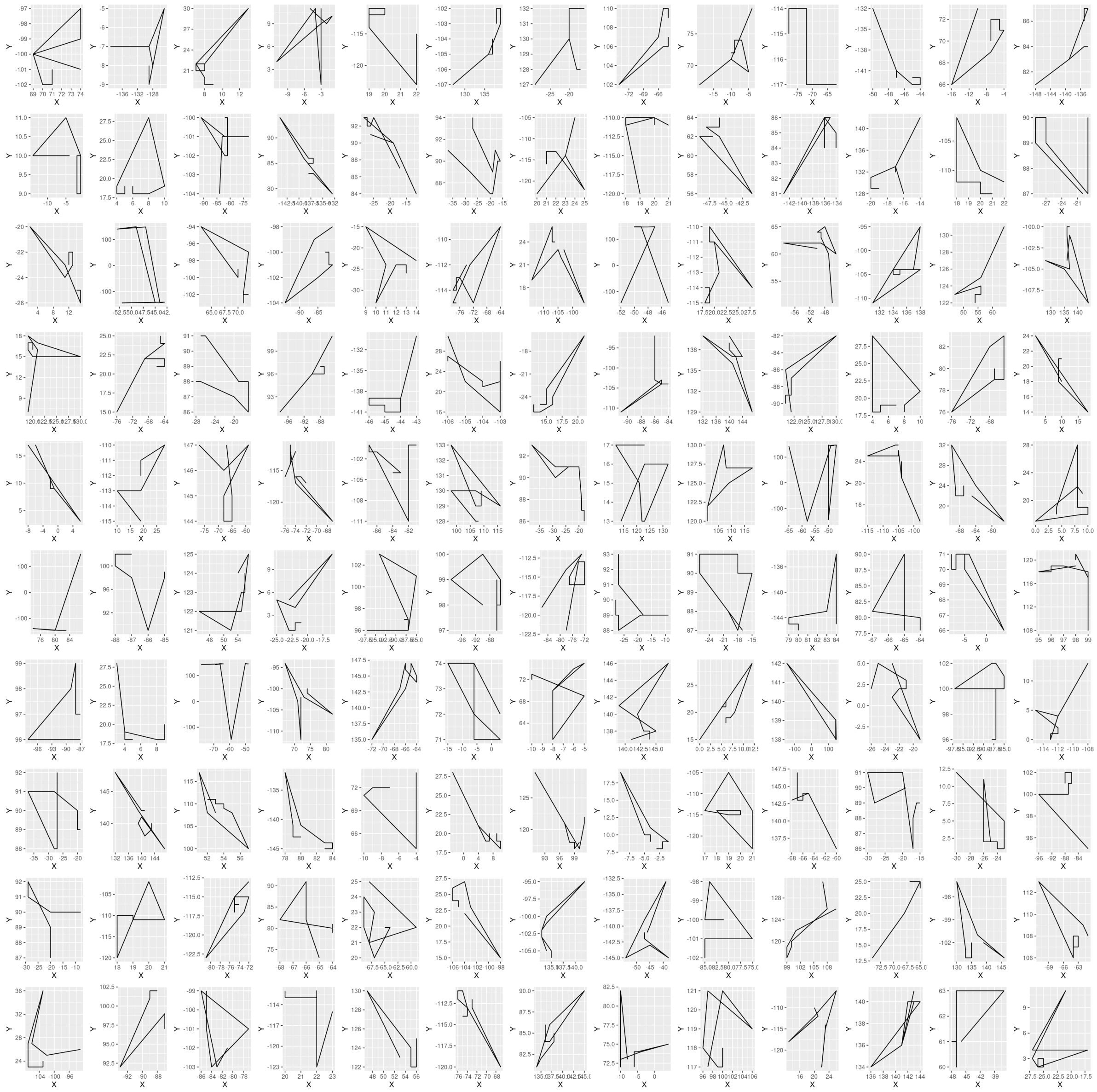

Figure A.7: 130 random 10-step segments from cluster C=6 (Table 1)

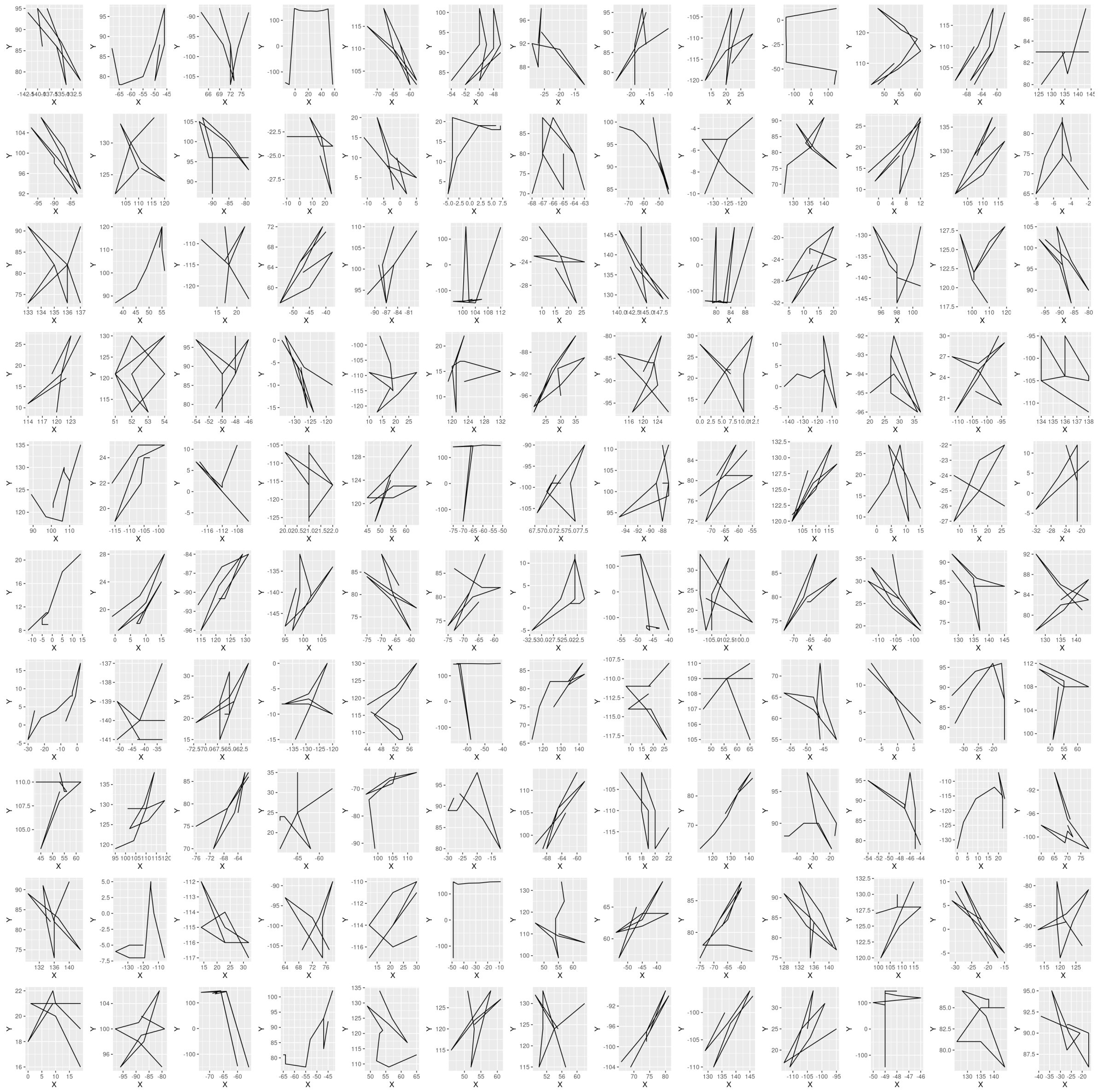

Figure A.8: 130 random 10-step segments from cluster C=7 (Table 1)

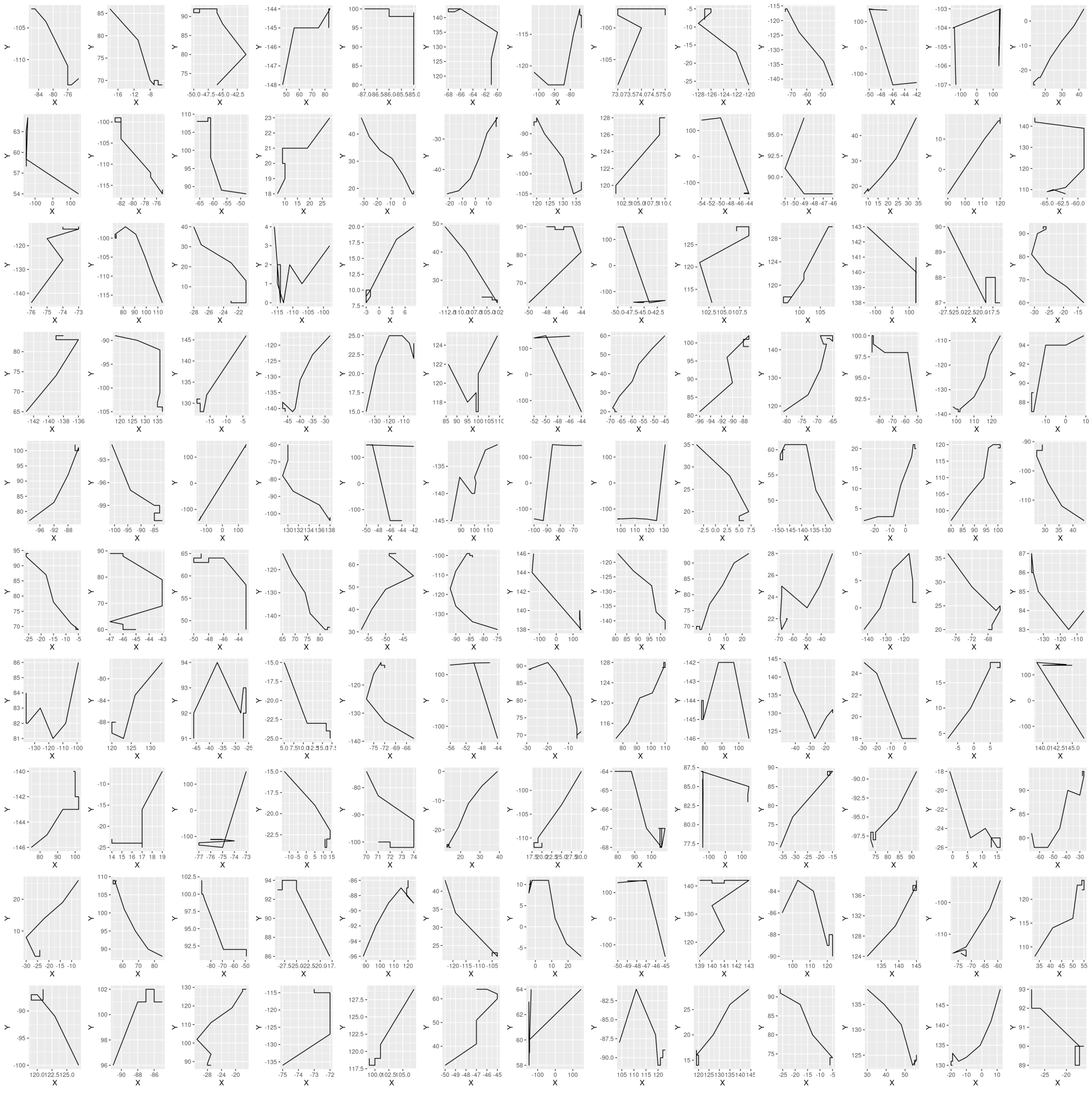

Figure A.9: 130 random 10-step segments from cluster C=8 (Table 1)

A.6 Plots of Adult Female Barn Owl StaMEs

Each plot in this subsection, particularly across different clusters, has had its axes for each panel automatically set by the plotting routine. Thus the size of segments is not comparable, only their relative shapes are informative.

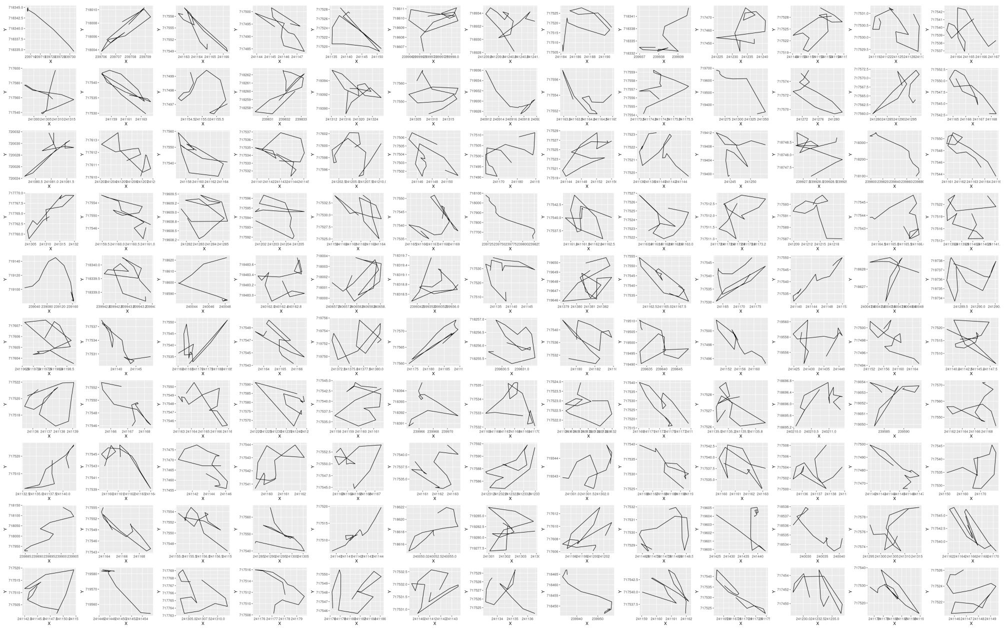

Figure A.10: 130 random adult female barn owl segments from cluster C=1 (Table 2)

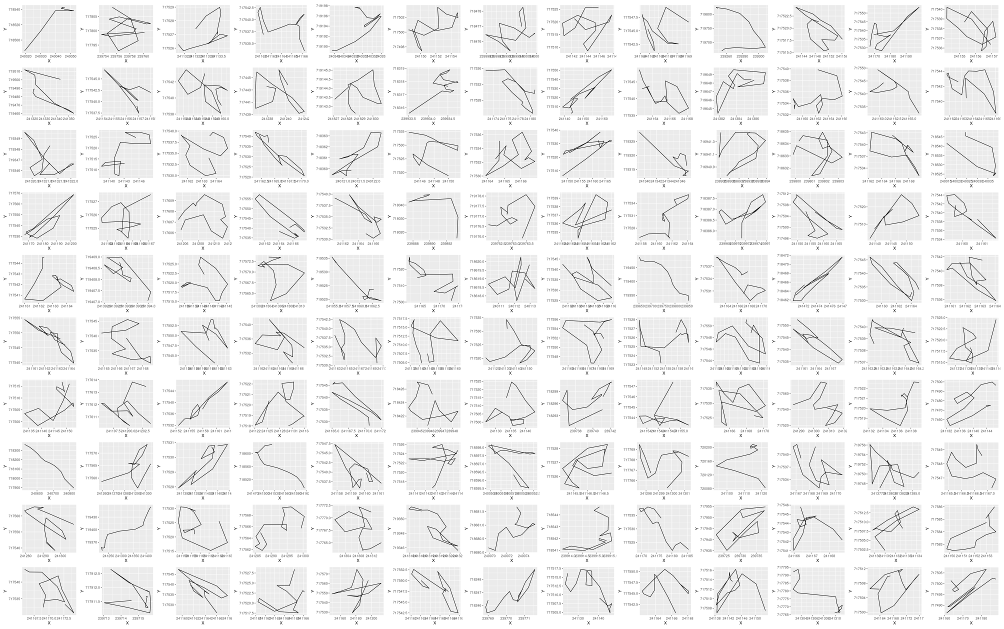

Figure A.11: 130 random adult female barn owl segments from cluster C=2 (Table 2)

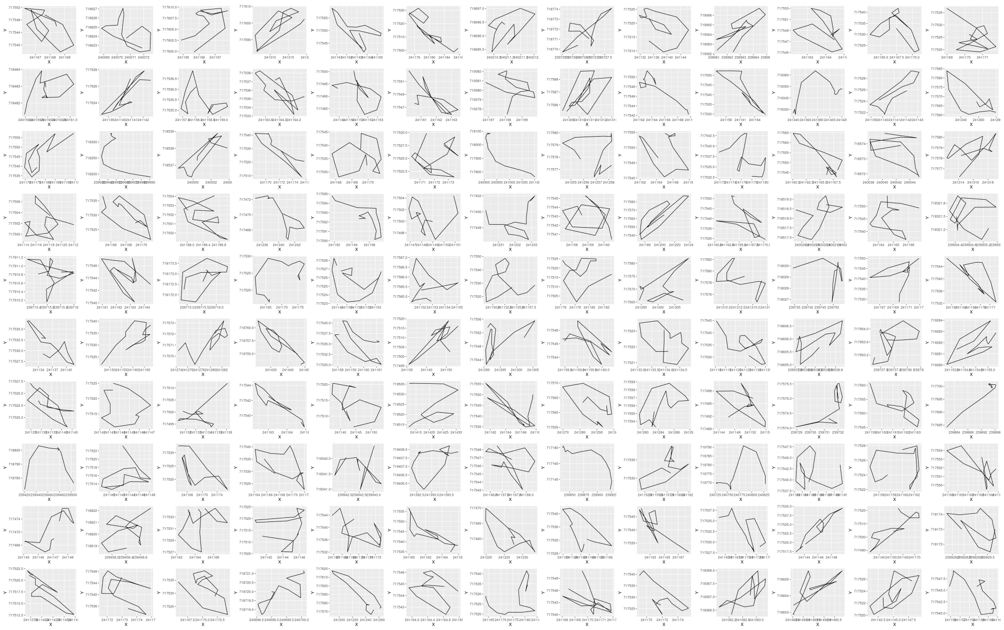

Figure A.12: 130 random adult female barn owl segments from cluster C=3 (Table 2)

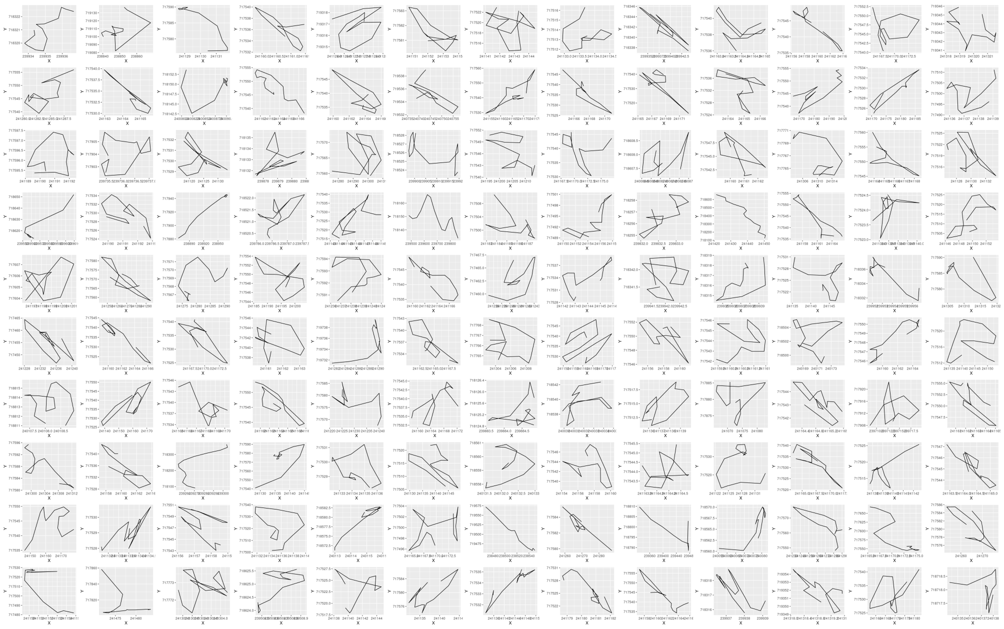

Figure A.13: 130 random adult female barn owl segments from cluster C=4 (Table 2)

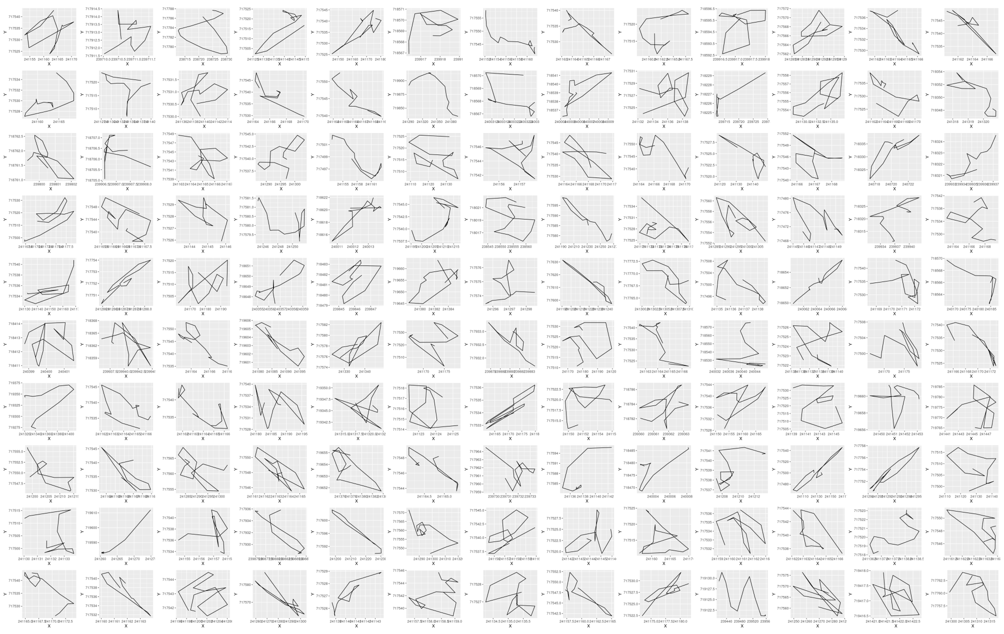

Figure A.14: 130 random adult female barn owl segments from cluster C=5 (Table 2)

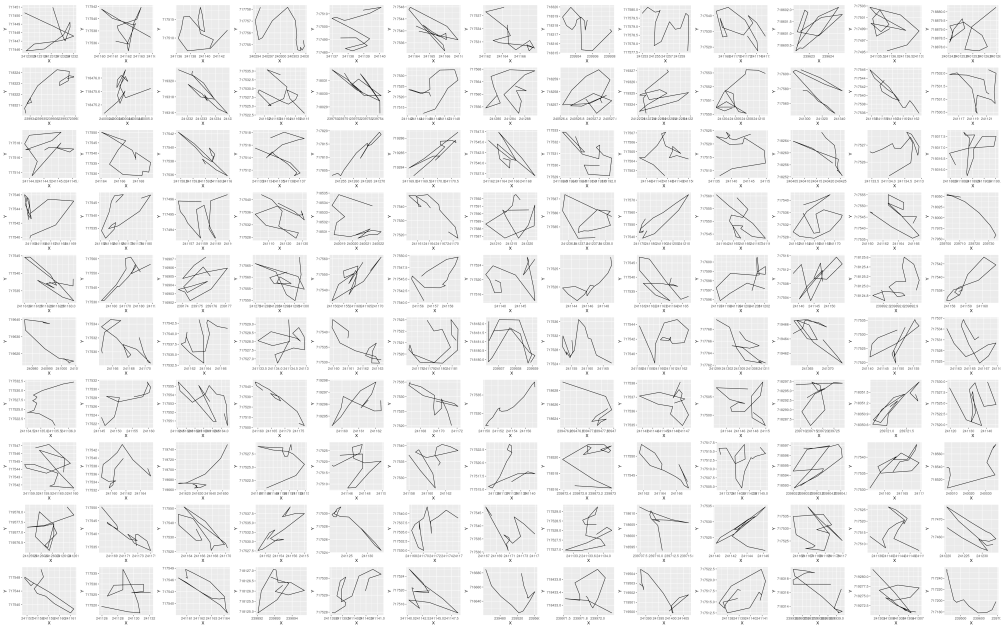

Figure A.15: 130 random adult female barn owl segments from cluster C=6 (Table 2)

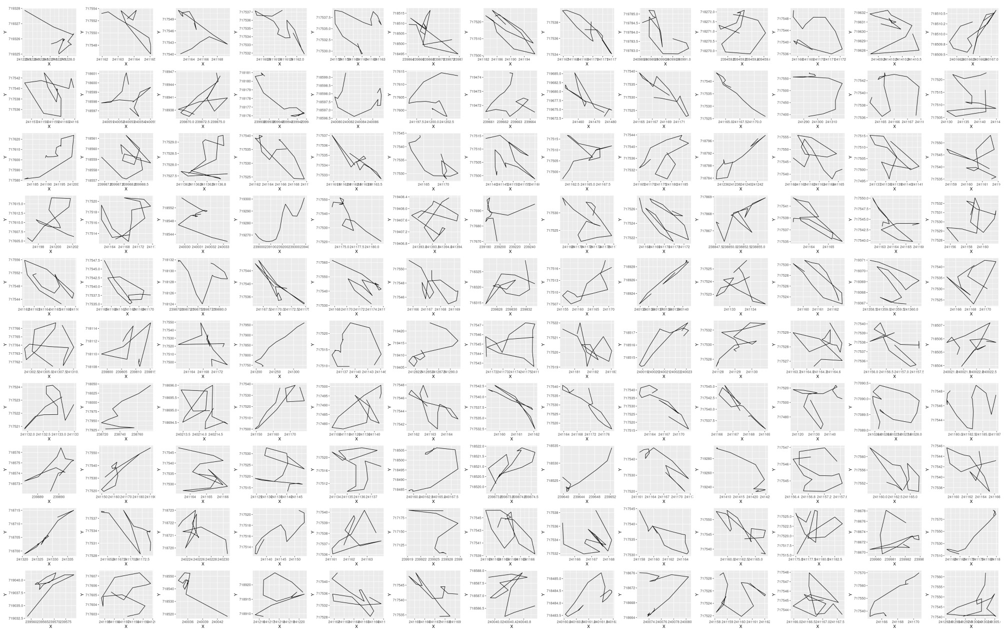

Figure A.16: 130 random adult female barn owl segments from cluster C=7 (Table 2)

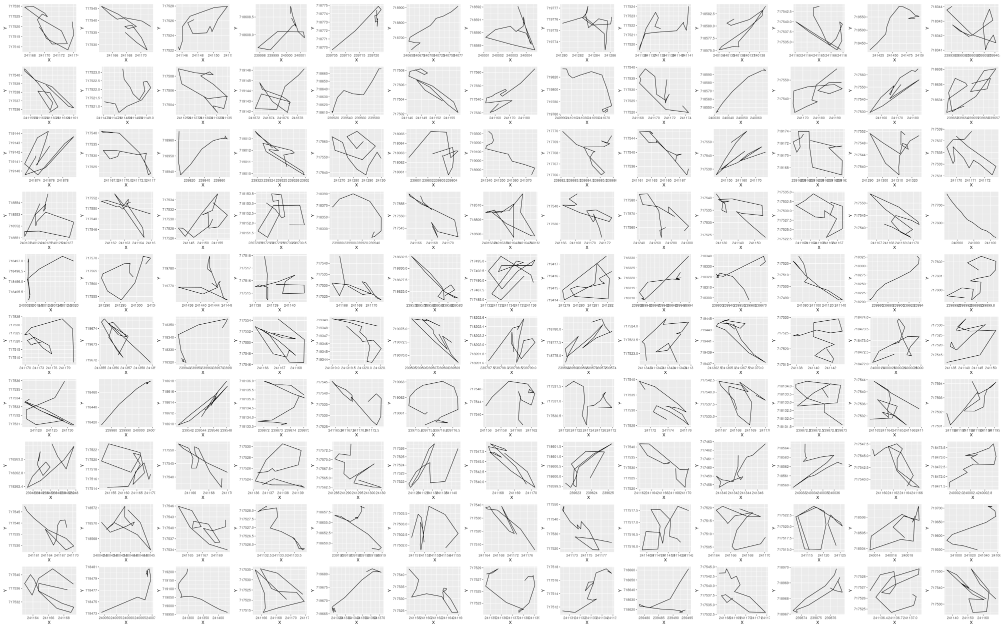

Figure A.17: 130 random adult female barn owl segments from cluster C=8 (Table 2)

A.7 Plots of Juvenile Male Barn Owl StaMEs

Each plot in this subsection, particularly across different clusters, has had its axes for each panel automatically set by the plotting routine. Thus the size of segments is not comparable, only their relative shapes is informative.

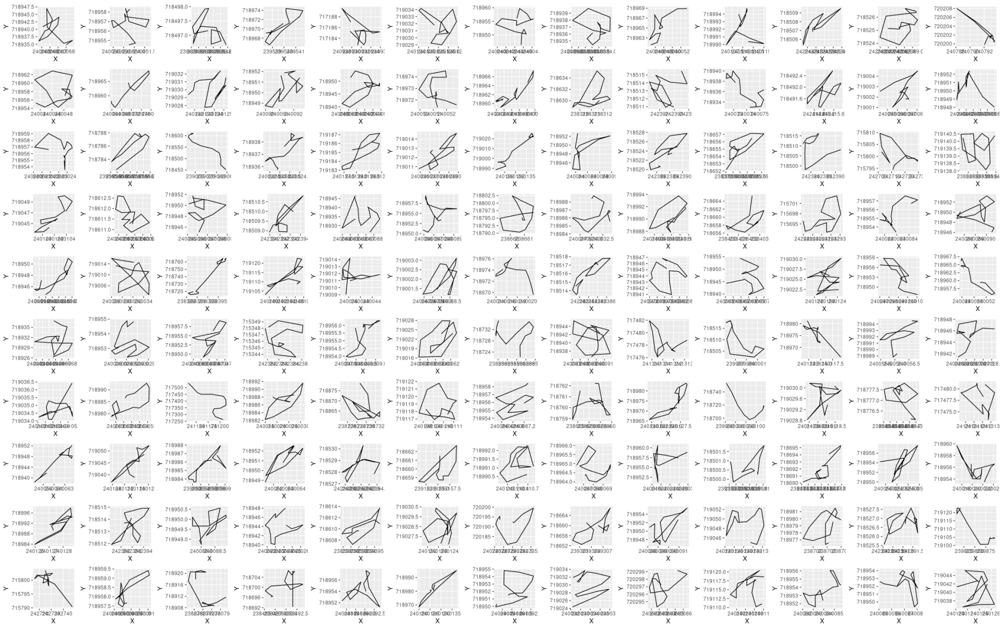

Figure A.18: 130 random adult female barn owl segments from cluster C=1 (Table 2)

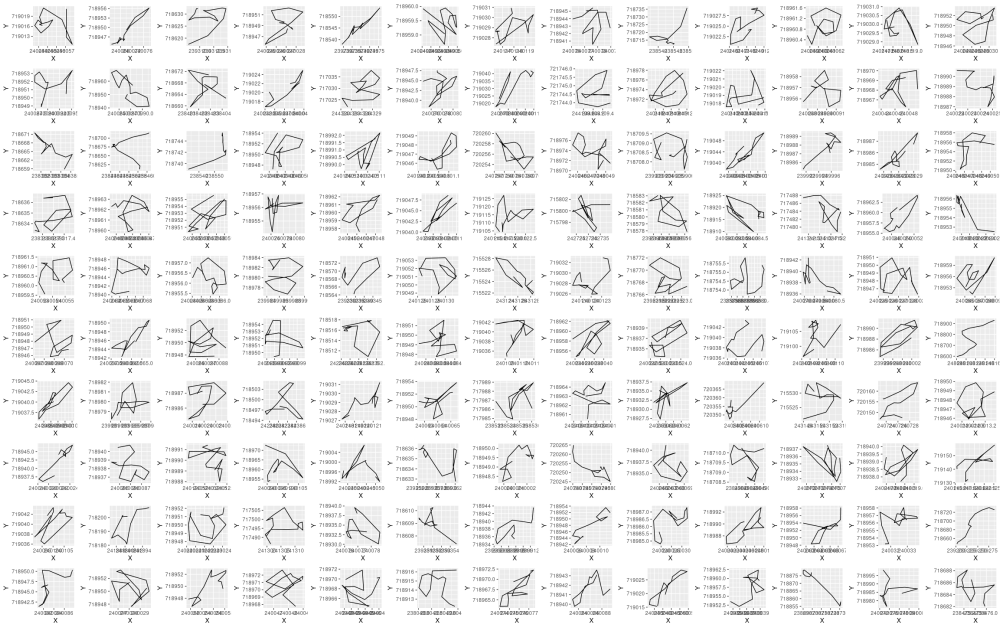

Figure A.19: 130 random juvenile barn owl segments from cluster C=2 (Table 2)

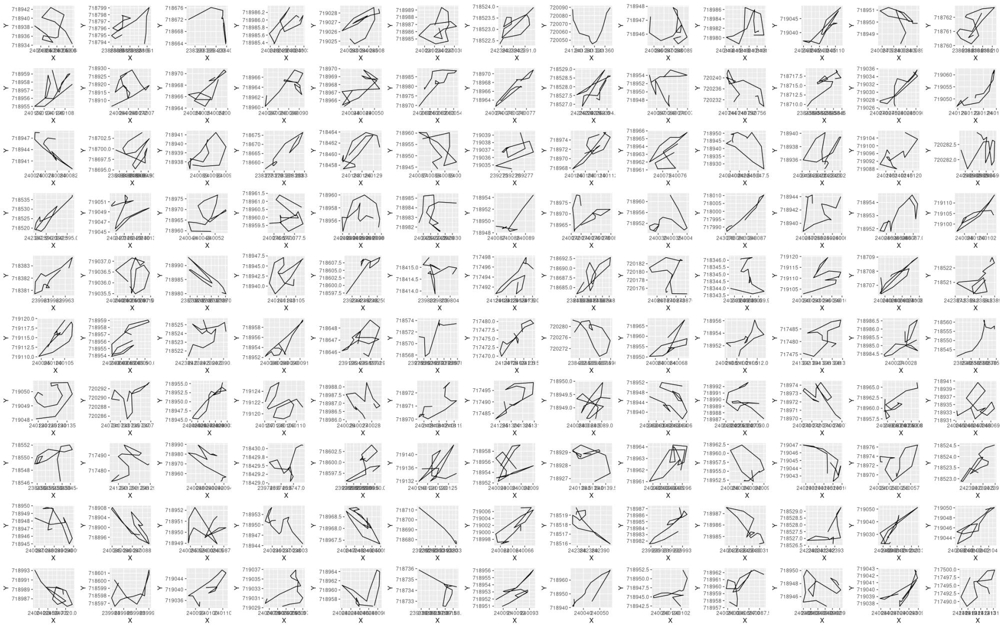

Figure A.20: 130 random juvenile barn owl segments from cluster C=3 (Table 2)

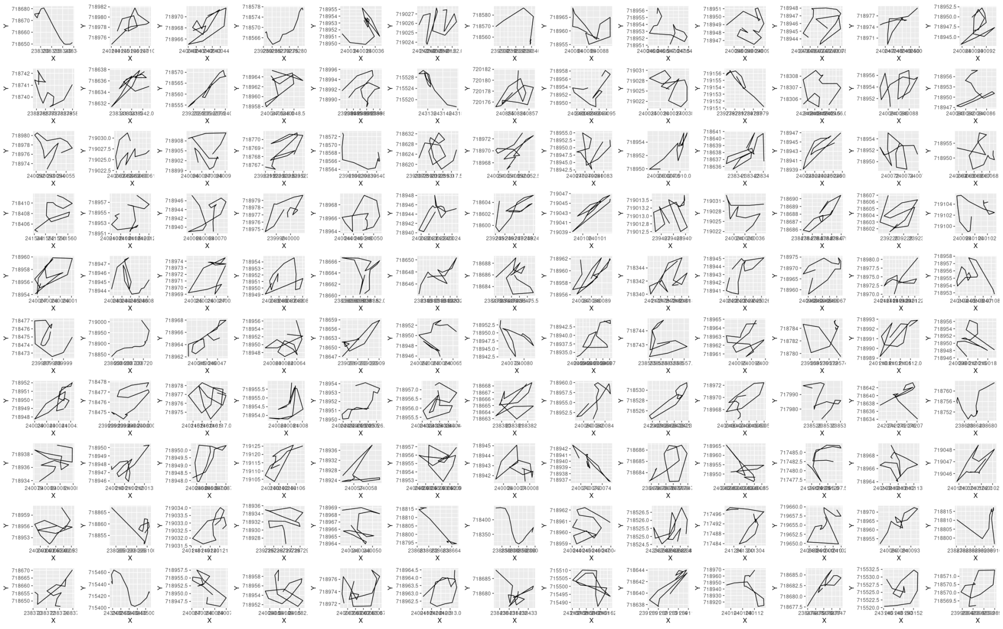

Figure A.21: 130 random juvenile barn owl segments from cluster C=4 (Table 2)

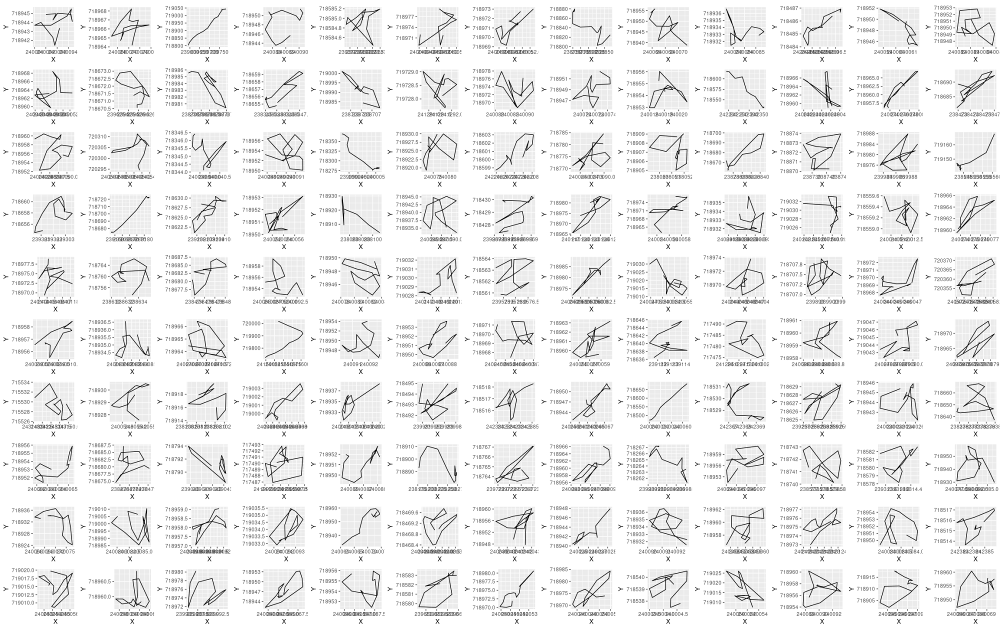

Figure A.22: 130 random juvenile barn owl segments from cluster C=5 (Table 2)

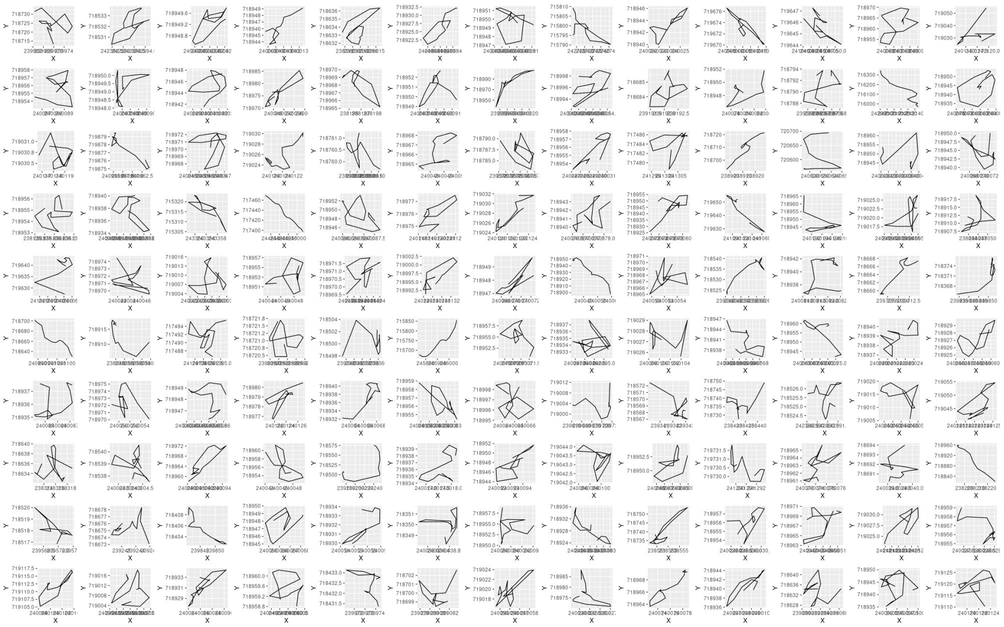

Figure A.23: 130 random juvenile barn owl segments from cluster C=6 (Table 2)

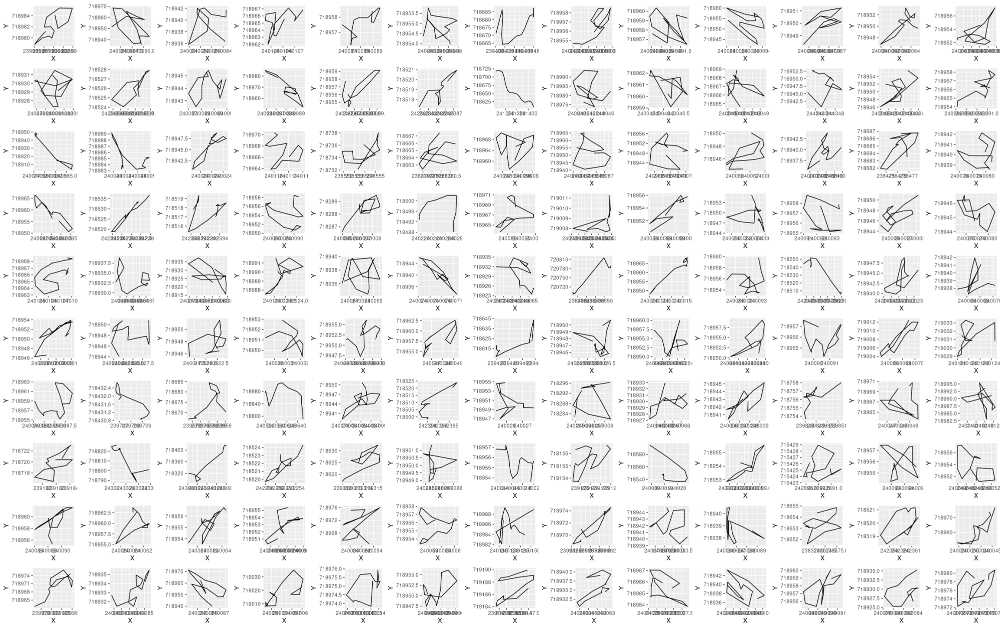

Figure A.24: 130 random juvenile barn owl segments from cluster C=7 (Table 2)

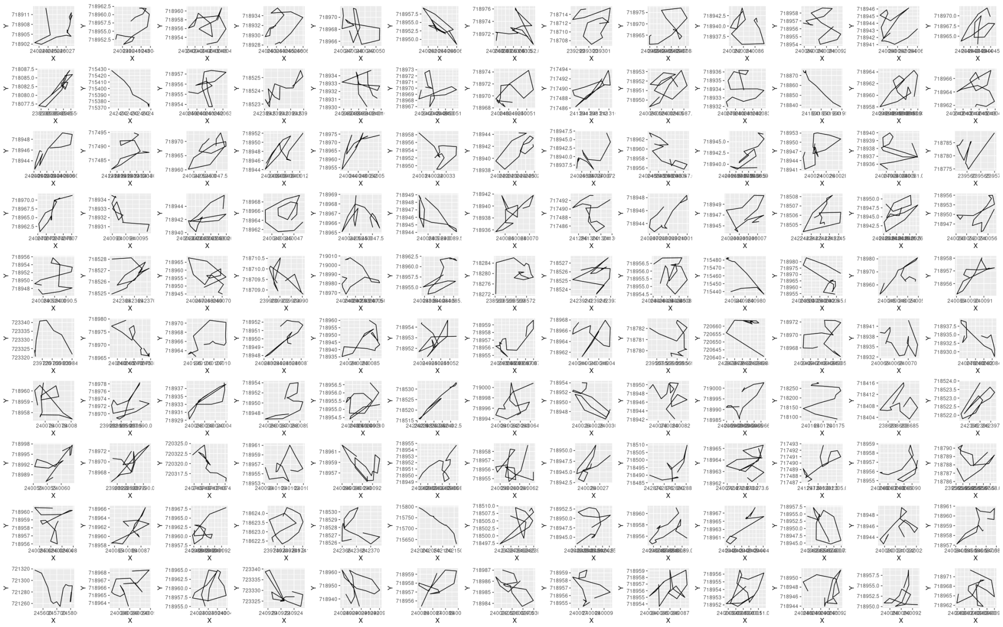

Figure A.25: 130 random juvenile barn owl segments from cluster C=8 (Table 2)

B Numerical construction of a homogeneous movement map

A numerical representation of the mapping $\mathcal{F}_\mu^{\rm hom}$ Eq 14, where we note that we have dropped the dependence on $\overline{\Delta^\rho}$ because of the lack of circular bias in our movement simulations and the index μ in the image space) can be constructed as follows:

1. For a set of kernels defined in terms a positive integer $\hat{r}^{\max}$ and an incremental angle $\Delta\psi$ (typically equal to $\pi/12$ or $\pi/36$) define the following set of kernels

$$\mathcal{K}\left(\hat{r}^{\max}, \Delta\psi\right) = \left\{K(r^{\min}, r^{\max}, \nu\Delta\psi)\,\middle|\, r^{\min} = 0, \cdots, r^{\max},\ r^{\max} = 1, \cdots, \hat{r}^{\max},\ \nu = 1, \cdots, \frac{\pi}{\Delta\psi}\right\} \tag{B.6}$$

2. For each kernel in $\mathcal{K}\left(\hat{r}^{\max}, \Delta\psi\right)$ run a simulation of the model, moving the individual over the desired homogeneous landscape for a selected period of period of time. For example, if one wants to generate 100 segments, each 15 points long, then the simulation will proceed for 1500 time steps.

3. For each of the simulated trajectories of the kernels in $\mathcal{K}\left(\hat{r}^{\max}, \Delta\psi\right)$, carry out a segmentation with segment size μ and generate the selected set of segment statistics, in our case the values $\left(V, {\rm SD}^V, |\Delta\Theta|, {\rm SD}^{|\Delta\Theta|}\right)$ for each segment. Compute the means of these statistics across the set of segments to obtain one candidate point of the mapping Eq 14.

4. In identifying the set of kernel $\left(r^{\min}, r^{\max}, \nu\Delta\psi\right)$ that generates an image under the mapping $\mathcal{F}_\mu^{\rm hom}$ that is closest to the observed point $\left(\overline{V}^{\rm obs}, \overline{{\rm SD}^V}^{\rm obs}, \overline{|\Delta\Theta|}^{\rm obs}, \overline{{\rm SD}^{|\Delta\Theta|}}^{\rm obs}\right)$ proceed as follows:

   (a) Discretize the image space of $\mathcal{F}_\mu^{\rm hom}$ through a series of latin cube lattice computations of $\mathcal{F}_\mu^{\rm hom}$ with arguments ranging over the cube with min and max bounding vertices $\left\{(0,0,0), (\hat{r^{\min}}, \hat{r^{\max}}, \hat{\nu}\Delta\psi)\right\}$ and intermediate cubes with min and max vertices $\left\{(r^{\min}, r^{\max}, \nu\Delta\psi), (r^{\min}+1, r^{\max}+1, (\nu+1)\Delta\psi)\right\}$, $r^{\min} = 0, \cdots \hat{r^{\min}} - 1$, $r^{\max} = 0, \cdots \hat{r^{\max}} - 1$, and $\nu = 0, \cdots, \hat{\nu} - 1$.

   (b) Select the intermediate cube that contains the image $\left(\overline{V}^{\rm obs}, \overline{{\rm SD}^V}^{\rm obs}, \overline{|\Delta\Theta|}^{\rm obs}, \overline{{\rm SD}^{|\Delta\Theta|}}^{\rm obs}\right)$: i.e., the cube for which, making the K_α arguments in $\mathcal{F}_\mu^{\rm hom}(K_\alpha)$ explicitly:

$$\mathcal{F}_\mu^{\rm hom}\left(r^{\min}, r^{\max}, \nu\Delta\psi\right) \le \left(\overline{V}^{\rm obs}, \overline{{\rm SD}^V}^{\rm obs}, \overline{|\Delta\Theta|}^{\rm obs}, \overline{{\rm SD}^{|\Delta\Theta|}}^{\rm obs}\right) \le \mathcal{F}_\mu^{\rm hom}\left(r^{\min}+1, r^{\max}+1, (\nu+1)\Delta\psi\right) \tag{B.7}$$

   (c) Either select the vertex in the cube defined by $\left\{(r^{\min}, r^{\max}, \nu\Delta\psi), (r^{\min}+1, r^{\max}+1, (\nu+1)\Delta\psi)\right\}$ whose image under $\mathcal{F}_\mu^{\rm hom}$ is closest to the observed point $\left(\overline{V}^{\rm obs}, \overline{{\rm SD}^V}^{\rm obs}, \overline{|\Delta\Theta|}^{\rm obs}, \overline{{\rm SD}^{|\Delta\Theta|}}^{\rm obs}\right)$ or use a finer resolution Latin Cube grid within this cube to locate a set of parameters the provides an image as close as desired to the observed point.

C ANIMOVER_1: Access and Data

C.1 Output Data

At the end of the simulation the output data can either be automatically or manually saved (using a toggle switch on the console, see Fig 5, N in main text) as a csv file. The header to this file contains a list of all the parameter and switch settings that were used to generate the run. In addition, the data are listed in 8 different columns depicted in Fig. C.1.

7	Day	Delta	X	Y	Distance	Theta (Deg.)	Resources	Kernel
715	8	14	-96	27	10	36.869898	135.24144	BP: Movement
716	8	15	-105	26	9.055385	186.34019	135.43017	WP: Rim
717	8	16	-106	25	1.414214	225	135.67697	BP: Vision
718	8	17	-110	16	9.848858	246.03751	135.67697	WP: Rim
719	8	18	-112	7	9.219544	257.47119	135.67697	BP: Movement
720	8	19	-114	2	5.385165	248.19859	135.96269	BP: Vision
721	8	20	-116	1	2.236068	206.56505	136.2484	BP: Vision
722	8	21	-112	3	4.472136	26.565051	136.53412	WP: Seeking
723	8	22	-112	4	1	90	136.81983	WP: Consuming
724	8	23	-111	4	1	0	137.10554	WP: Consuming
725	8	24	-109	-5	9.219544	282.52881	137.10554	WP: Rim
726	8	25	-111	-14	9.219544	257.47119	137.10554	BP: Movement
727	8	26	-111	-24	10	270	137.10554	BP: Movement
728	8	27	-108	-33	9.486833	288.43495	137.10554	BP: Movement
729	8	28	-103	-41	9.433981	302.00538	137.10554	BP: Movement
730	8	29	-99	-50	9.848858	293.96249	137.10554	BP: Movement
731	8	30	-92	-56	9.219544	319.39871	137.10554	BP: Movement
732	8	31	-86	-63	9.219544	310.6013	137.10554	BP: Movement
733	8	32	-84	-72	9.219544	282.52881	137.10554	BP: Movement
734	8	33	-84	-82	10	270	137.10554	BP: Movement
735	8	34	-86	-91	9.219544	257.47119	137.10554	BP: Movement
736	8	35	-83	-100	9.486833	288.43495	137.39126	BP: Vision
737	8	36	-87	-102	4.472136	206.56505	137.67697	BP: Vision
738	8	37	-84	-103	3.162278	341.56505	137.96269	WP: Seeking
739	8	38	-84	-103	0	0	138.2191	WP: Consuming
740	8	39	-84	-104	1	270	138.50481	WP: Consuming
741	8	40	-85	-104	1	180	138.79053	WP: Consuming
742	8	41	-85	-103	1	90	139.07624	WP: Consuming
743	8	42	-85	-103	0	0	139.33265	WP: Consuming
744	8	43	-85	-102	1	90	139.61837	WP: Consuming
745	8	44	-85	-101	1	90	139.90408	WP: Consuming
746	8	45	-85	-101	0	0	140.16049	WP: Consuming
747	8	46	-85	-100	1	90	140.4462	WP: Consuming
748	8	47	-85	-99	1	90	140.73192	WP: Consuming
749	8	48	-85	-99	0	0	140.98833	WP: Consuming
750	8	49	-85	-99	0	0	141.20201	WP: Consuming
751	8	50	-85	-100	1	270	141.45875	WP: Consuming
752	8	51	-84	-100	1	0	141.74446	WP: Consuming

10-point Segments

Pure

Mixed

Figure C.1: The 8 columns in the csv data output file (e.g., see the file `Two_Kernel_Movement.csv`, Supplementary Data) that can be saved at the end of the simulation as as follows: `Day`, `Delta` (within-day step), `X` (x-location), `Y` (y-location), `Distance` (distance moved), `Theta` (angle of heading in degrees), `Resources` (agent-state), `Kernel` (BP or WP). We note that one computes the turning angle $\Delta\theta$ at time t by subtracting the angle of headings at time t from the angle of heading at time $t-1$ taking into account that angles are specified modulo 360 degrees (e.g., if $\theta_{t-1} = 350$ and $\theta_t = 10$, then the turning angle is $\Delta\theta = 10 - (350 - 360) = 20$ degrees). Thus angles have heading range over $[-\pi, \pi]$. In addition, the third argument in our StaME is the average of the absolute values of the angles of heading rather than just the angles themselves. Also note that the BP movement mode (green entries) has two states: `Movement` (movement within the kernel rim which rim exists whenever $r_{\text{wp}}^{\text{min}} > 0$) or `Vision` (movement within the kernel sector, possibly between the inner rim radius and the current location). The WP movement mode has three states: `Consuming` (when moving to a resource-rich location), `Seeking` (an interim step in looking for a resource rich location when the kernel contains insufficient resources), or `Rim` moving the maximum step length ($r_{\text{bp}}^{\text{max}}$ rather than $r_{\text{wp}}^{\text{max}}$ when the seeking state fails to provide a suitable resource location). The indicted 10-step segmentation of the data shows how both pure and mixed movement segments arise during segmentation.

C.2 Downloading and Running the App

ANIMOVER_1 and the most recent release of Numerus Studio can be downloaded without cost from the Numerus webpage https://www.numerusinc.com/studio/. Installers for Numerus Studio are provided for Mac and Windows platforms. Instructions for using Numerus Studio are contained in the RAMP Users Guide at https://wiki.numerusinc.com/index.php/Ramp_User_Guide

ANIMOVER_1 is deployed in the RAMP file `Ani1Cr3.nms`. After installing Numerus studio, open this file and launch it from the Studio launchpad. Documentation for this RAMP can be found at https://wiki.numerusinc.com/index.php/Animover_1.

D ODD Protocol for ANIMOVER_1

The element numbering scheme used in the subsections below is that of the second ODD revision present in the Grimm et al. [64].

D.1 Purpose and Patterns

The reason for developing an animal movement simulator is articulated under goal c.) in the Introduction of the main text. More broadly, we construct a user friendly, highly flexible movement track simulator that can be used to test ideas and concepts. This includes testing hypotheses about underlying mechanisms that may lead to particular emergent patterns of movement behavior [112]. In addition, simulated data can be used, as we did to a limited extent in this paper and now do more extensively in our ongoing research, to evaluate methods for movement track analysis, and forecasting animal movement patterns in changing and novel environments. Although our simulator is built at two scales—1.) next-step decisions of where to move next using ideas from step-selection function analysis [55], 2.) time-already-spent-in-current-movement variables (e.g., mimicking satiation, increasing thirst, or hunger effects) and time-within-diel-cycle variables (e.g., when to head home)—patterns emerge at a third scale (different kinds of diel activity routines [43]), with higher scale seasonal patterns emergent as well.

D.2 Entities, state variables,and scales

The relevant entities and state variables are listed in Table 3. They are cells (as represent by their position and associated euclidean location in the cellular array $\mathcal{A}$), the resource states $c_{ab,t}$ of cells at time t, the individual agent's location $(x_t^{\rm id}, x_t^{\rm id})$, movement mode α_t, angle of heading θ_t, time in current movement mode t_t^s, and internal state value h_t (e.g., level of resource satiation or its inverse, hunger).

D.3 Process overview and scheduling

The process overview and scheduling are illustrated in Fig 3, which depicts the computational flow sequence of the movement decision algorithm at the core of ANIMOVER_1. In short, after selecting parameter values and setting up the initial patch structure of the landscape (details next), the algorithm loops through a next-step process. This involves 1.) computing the location to which the individual moves from its current location based on the individual's current movement mode and the summed resource state of the cells in its current neighborhood, 2.) computing the amount of resources the individual extracts from the location to which it moves, thereby updating its current state to include these new resources and the cost of movement, 3.) updating the resource state of the cell to which the agent moves and from which it extracts resources, 4.) updating the current movement mode based on the state of the neighborhood of the agent's current location, 5.) implementing the STOP rule when either the end of the simulation has been reached ($t = T$) or the individuals internal state has hit 0 ($h_t = 0$; the individual is now dead).

D.3.1 Patch setup

The process for setting up the initial patch structure of the landscape is implemented by the runtime alterable module $\texttt{RAM}_{\#}^{\rm patch}$, where the details of the different possible preselected versions $\# = 0$ (default), $\# = 1$ (selectable alternative), #2 (for use to supply customized code) are elaborated in Section 4.2 of the main text.

D.3.2 Resource extraction

The process for computing the amount of resource extracted from the current cell in which the agent is located and the new state of the agent as a function of the amount of resource it extracts is implemented by the runtime alterable module $\texttt{RAM}_{\#}^{\rm val}$, where the default version $\texttt{RAM}_{0}^{\rm val}$ uses the resource density independent equations, Eq 6, and the alternative version $\texttt{RAM}_{1}^{\rm val}$ uses the resource density-dependent equations, Eq 7. It

is also possible for the user to insert a customized set of equations by coding their own version $\texttt{RAM}_2^{\text{val}}$ of this RAM.

D.3.3 Next step computation

The next-step computations are implemented by the within-patch and between-patch step selection procedures $\mathcal{R}_{\text{wp}}(\hat{t}^{\text{s}_{\text{wp}}}, \hat{c}^{\text{nbh}}_{\text{wp}})$ and $\mathcal{R}_{\text{bp}}(\hat{t}^{\text{s}_{\text{wp}}}, \hat{c}^{\text{nbh}}_{\text{bp}})$ provided in Appendix A.3 above.

D.4 Design concepts

In the ODD protocol, this sections describes how the following 11 concepts were considered in the model.

D.4.1 Basic Principles

The basic principle behind our movement algorithm is that an individual chooses were to move next based on its current mode of movement (e.g., is it searching for resources or commuting to a new location), the statistics of the step-size and turning angles associated with its current movement mode, and the best location to land on its "next-step" given the state of the landscape within a radius of the largest value associated with the distribution of step sizes for its current movement mode.

D.4.2 Emergence

Emergence relates to patterns produced by a sequence of steps associated with the current movement mode, and by switching several times among movement modes to produce patterns at the scale of BAMs (e.g.: resting that emerges from a sequence of small, directionally random StaMEs; foraging that emerges from a mixture of medium and short StaMEs with relatively high turning angles; commuting that emerges from a sequence of large StaMEs with relatively low turning angles), DARS (e.g., commuting to a known distant location where feeding occurs and then returning versus interspersed searching and resting close to a home location), LiMPs (e.g., migration, ranging, or territoriality phases) and LiTs (a central place forager, versus a ranger, or an annual migrator; also see Fig 1 in main text).

D.4.3 Adaptation

Adaptation of movement is implicit in the model, first in the way movement kernels are impacted by the current landscape structure (see Fig 2C) and second in the fact that the state equations, depending on the particular $\texttt{RAM}_{\#}^{\text{val}}$ used (see above), contain feedback structures that result in density-dependent resource extraction. Additionally, we note that as the simulation progress, resource consumption by the agent during the early phases of the simulation reshapes the landscape and hence my lead to shift in the agent's emergent movement patterns over time.

D.4.4 Objectives

From the agents point of view, the objective is to maximize resource intake at each within patch movement step. This is realized through rule $\mathcal{R}_{\text{wp}}.2$ (Appendix A.3) where the maximum value $c_{ab}^{\max}$ is computed and decisions where to move next are based upon the location of the cell that has this value.

D.4.5 Learning

An ability to adapt through learning is not included in ANIMOVER_1.

D.4.6 Prediction

As mentioned under element 1 (D.1 above), "forecasting animal movement patterns in changing and novel environments" is one of the purposes of the model. The ability of the model to reliably predict where an animal moves next, as well as forecast patterns of movement at larger scales requires that the parameters of the model first be fitted to the data using an estimation procedure suitable for the task in the context the type of model and data available [113]. This has not been done here since the third goal of this paper is both make available the ANIMOVER_1 simulation for the purpose elaborated in element 1 (D1) above and also, as articulated in the Introduction of the main text. Model parameter estimation is data specific and so will need to be undertaken in any studies that involve using ANIMOVER_1 to predict the movement of individuals by fitting kernel and state updating parameters to empirical data on both the movement and state of those individuals.

D.4.7 Sensing

An agent sensing the state of the environment is a very important component of ANIMOVER_1. This is done through the definition of movement-mode specific full and rim circle-sector kernels (Fig 2). Looking closely out the output in Fig C.1, in the last column ("Kernel") 5 types of kernels are identified in the context of between-patch (BP) and within-patch (WP) movement. Specifically we see BP:Movement, BP:Vision, WP:Seeking, WP:Consuming, and WP:Rim. The BP:Movement and WP:Seeking are just the next-step between-patch and within-patch selection kernels. The BP:Vision kernel is invoked when an individual hits a landscape boundary during between-patch movement and thus needs to look all around (including behind itself) to make its next move (see $\mathcal{R}_{\rm bp}$.2 in Section A.3). The WP:seeking kernel is invoked with an individual within a patch does not find a cell to move to that has resources that exceeds its threshold requirements in which case it enlarges and fills out its current movement rim to a full movement sector (see $\mathcal{R}_{\rm bp}.4c$ in Section A.3. If this kernel then fails to find a suitable cell for its next step, it moves at random to the best cell available in the kernel WP:Rim.

D.4.8 Interaction

In ANIMOVER_1 is a single-agent simulator and interactions are confined to a resource-consumption process involving the resources within the cell where the agent is currently located, according to Eqs 6 and 7. Clearly a multi-agent simulator can be developed where interactions among individuals with respect to both movement (either attraction or repulsion elements can be included) and resource exploitation (competition for resources among individuals in the same cell can be introduced into Eqs 6 and 7 using approaches such as those discussed in [54]

D.4.9 Stochasticity

Stochasticity enters into several different places in the simulation. First, the process of setting up patches in $\texttt{RAM}^{\rm patch}$ involves laying down a set of patch seeds and then randomly adding neighbors to these seeds with a certain probability, as articulated in Section 2.2 of the main text. Second, movement to a particular cell in the within and between patch movement procedures $\mathcal{R}_{\rm wp}$ and $\mathcal{R}_{\rm bp}$ involves a multinomial drawing in which cells higher resource cells falling within the movement kernel in operation at time are more likely to be selected than lower resource cells.

D.4.10 Collectives

Not applicable to ANIMOVER_1.

D.4.11 Observation

It is assume in ANIMOVER_1 that the simulate locations of the agent as it moves over the landscape are observed without error. If observation errors need to be introduced into this process, then noise can be

added to the output in a form desired for the analysis hand.

D.5 Initialization

Initialization of the landscape patch structure is dealt with in Section 2.2 of the main text. The process for initializing other model parameters is discussed in Section 4.3, P6 and P7.

D.6 Input data

The process for initializing the landscape structure by reading in an input file is discussed in Section 4.3, as is the process for setting all the parameter values for the run.

D.7 Submodels

D.7.1 Landscape construction

This is handled by $\texttt{RAM}^{\text{patch}}$, as discussed in D3.1 above.

D.7.2 Resource extraction

This is handled by $\texttt{RAM}^{\text{val}}$, as discussed in D3.2 above.

D.7.3 Next step computation

This is handled by algorithms (step selection rules/procedures) $\mathcal{R}_{\text{wp}}(\hat{t}^{\text{s}_{\text{wp}}}, \hat{c}^{\text{nbh}}_{\text{wp}})$ and $\mathcal{R}_{\text{bp}}(\hat{t}^{\text{s}_{\text{wp}}}, \hat{c}^{\text{nbh}}_{\text{bp}})$ provided in Appendix A.3.
